# Revisiting the evolution of mouse LINE-1 in the genomic era

**DOI:** 10.1186/1759-8753-4-3

**Published:** 2013-01-03

**Authors:** Akash Sookdeo, Crystal M Hepp, Marcella A McClure, Stéphane Boissinot

**Affiliations:** 1 Department of Biology, Queens College, the City University of New York, 65-30 Kissena Boulevard, Flushing, NY 11367-1597, USA; 2 School of Life Sciences, Arizona State University, Tempe, AZ, USA; 3 Center for Evolutionary Medicine and Informatics, Biodesign Institute, Arizona State University, Tempe, AZ, USA; 4 Department of Microbiology, Montana State University, Bozeman, MT, USA; 5 The Graduate Center, the City University of New York, New York, NY, USA

**Keywords:** Retroposon, Retrotransposon, LINE-1, L1, *Mus musculus*, Recombination

## Abstract

**Background:**

LINE-1 (L1) is the dominant category of transposable elements in placental mammals. L1 has significantly affected the size and structure of all mammalian genomes and understanding the nature of the interactions between L1 and its mammalian host remains a question of crucial importance in comparative genomics. For this reason, much attention has been dedicated to the evolution of L1. Among the most studied elements is the mouse L1 which has been the subject of a number of studies in the 1980s and 1990s. These seminal studies, performed in the pre-genomic era when only a limited number of L1 sequences were available, have significantly improved our understanding of L1 evolution. Yet, no comprehensive study on the evolution of L1 in mouse has been performed since the completion of this genome sequence.

**Results:**

Using the Genome Parsing Suite we performed the first evolutionary analysis of mouse L1 over the entire length of the element. This analysis indicates that the mouse L1 has recruited novel 5’UTR sequences more frequently than previously thought and that the simultaneous activity of non-homologous promoters seems to be one of the conditions for the co-existence of multiple L1 families or lineages. In addition the exchange of genetic information between L1 families is not limited to the 5’UTR as evidence of inter-family recombination was observed in ORF1, ORF2, and the 3’UTR. In contrast to the human L1, there was little evidence of rapid amino-acid replacement in the coiled-coil of ORF1, although this region is structurally unstable. We propose that the structural instability of the coiled-coil domain might be adaptive and that structural changes in this region are selectively equivalent to the rapid evolution at the amino-acid level reported in the human lineage.

**Conclusions:**

The pattern of evolution of L1 in mouse shows some similarity with human suggesting that the nature of the interactions between L1 and its host might be similar in these two species. Yet, some notable differences, particularly in the evolution of ORF1, suggest that the molecular mechanisms involved in host-L1 interactions might be different in these two species.

## Background

Long interspersed nuclear element-1 (LINE-1 or L1) constitutes the dominant category of transposable elements in mammalian genomes. L1s have accumulated in the genomes of their mammalian hosts in extremely large numbers and contribute to more than 20% of genome size in human and mouse [1,2]. L1s have been a rich source of evolutionary novelties by providing motifs that can be recruited by the host either for the regulation of its own genes or within its coding sequences [3-6]. However, L1 activity can also be detrimental to the fitness of the host [7,8], either by inserting within genes [9,10] or by mediating chromosomal rearrangements through ectopic (non-allelic) recombination [11,12]. L1 elements replicate using a copy-and-paste mechanism that involves the reverse-transcription of the L1 RNA at the insertion site [13-15]. L1 encodes the replicative machinery necessary for the retrotransposition reaction. It contains two open-reading frames (ORFs) that are both indispensable for L1 retrotransposition. ORF1 encodes a trimeric protein with RNA-binding properties and nucleic-acid chaperone activity [16-20]. ORF2 encodes an endonuclease that makes the first nick at the insertion site and a reverse-transcriptase that copies L1 RNA into DNA at the site of insertion [21,22]. L1 has a 5’ untranslated region (UTR) that acts as an internal promoter [23,24] and a 3’ UTR with a conserved poly-G tract of unknown function [25]. The L1 retrotransposition reaction produces mostly 5’ truncated elements that are transpositionally inactive [26,27]. As the vast majority of L1 insertions do not serve a function for the host, they accumulate mutations at the neutral rate so that young families of L1 elements are less divergent than older ones [28-32].

The pattern of L1 element evolution in mammals is very unusual. In most species analyzed so far, L1 evolves as a single lineage: a family of elements emerges, amplifies to hundreds or thousands of copies and then becomes extinct, being replaced by a more recently evolved family [30,33-35]. This process is exemplified in human where a single lineage of replicatively dominant families has evolved over the last 40 MY [30]. The reason(s) why L1 evolves as a single lineage remains unclear but the similarity between L1 and H3N2 influenza A virus evolution [36-38] suggests that the single lineage mode of evolution could result from a co-evolutionary arms race between L1 and its host. This hypothesis is supported by the observation that the coiled-coil domain of ORF1 harbors the signature of adaptive evolution, possibly in response to host repression [39], and that adaptive evolution apparently correlates with the replicative success of L1 families [30]. However, in early primate evolution (from 70 to 40MY), multiple L1 lineages have co-existed in the human genome [30]. Interestingly, co-existing lineages always had non-homologous 5’UTRs suggesting that their co-existence could be due to their reliance on different host factors for their transcription.

The patterns described above result mostly from the analysis of the human genome and it is unclear how patterns of evolution in human recapitulate L1 evolution in other species. It is thus important to examine in greater detail the evolution of L1 lineages in other mammals. Pre-genomics studies in the house mouse (*Mus musculus*) have demonstrated the presence of multiple concurrently active L1 families with non-homologous promoters [33,40-48]. Recently active families are classified into two groups based on their promoter types (A or F types), whereas ancestral L1 families carry a third promoter, the V type. The co-existence of multiple L1 families with different promoters in extant mice recapitulates the situation in early primate evolution and provides a unique opportunity to investigate the interactions between concurrent L1 families and the molecular properties that would allow for such co-existence.

Previous L1 studies in mice were limited to sequence analysis performed on a few L1 loci, the majority of which were fragments of L1 inserts. No detailed study of L1 evolution in mouse has been performed since the completion of the mouse genome sequence [2]. With the availability of this genome, we decided to perform a comprehensive analysis of full-length L1 elements to investigate the evolutionary dynamics of L1 in mouse. We present evidence that the diversification of mouse L1 has been influenced by frequent events of recombination across the entire length of the element, rapid structural changes in ORF1, as well as lateral transfer by inter-specific hybridization.

## Results

A total of 20,459 L1 inserts with complete reverse transcriptase (RT) domains were identified using the Genome Parsing Suite (GPS). L1 elements were first grouped based on their 5’UTR. This was done by comparing the 5’ end of all elements with a library of previously described mouse 5’UTR using the Repeatmasker program [49]. The A, F, V, and Lx 5’UTR types have long been characterized [33,50,51] and the majority of elements could be assigned to one of these 5’UTR sequences. A number of elements however carried 5’UTRs distinct from these four types. These elements were aligned to each other and grouped into three novel types of 5’UTR: (1) a 5’UTR with similarity to the F type but with distinctive features, named F_anc_ (for F ancestral); (2) a 5’UTR that was not characterized before, named Mus (because it is absent from the rat genome); and (3) a 5’UTR that shows no similarity with any others, named N (for novel).

Once elements were sorted based on their 5’UTRs, they were further categorized into families using a phylogenetic analysis of the 3’ terminus. A family is defined as a collection of elements that result from the activity of a highly homogenous group of progenitors, which are characterized by a unique combination of characters. In the first step of the phylogenetic analysis, neighbor joining trees [52] of elements sharing similar 5’UTRs were built. Distinct clusters of sequences were provisionally considered families and were validated by a second round of phylogenetic analysis based on the principle that elements belonging to the same family should yield a star phylogeny (that is, a phylogenetic tree devoid of structure) because these elements result from the activity of very similar progenitors. These families were further confirmed by phylogenetic analysis performed on other regions of L1 to ensure that the homogeneity of the families extend over the entire length of the element.

Using this approach we identified 29 families and consensus sequences were derived for each of them (Table 1, Additional file 1, and Additional file 2). The number of variable sites in ORF1, ORF2, and the 3’UTR is 1,441 (25.1% of the total number of sites), 991 (17.2%) of which are parsimony-informative. The number of variable sites differs among regions, ORF2 having the largest number (785 out of 3,835 sites) followed by the 3’UTR (324 out of 652) and ORF1 (318 out of 1,218). However, ORF2 has the least number of variable and parsimony-informative sites relative to its length (20.5% and 13.9%, respectively) and the 3’UTR the most (49.7% and 32.5%), ORF1 having an intermediate number (26.1% and 19.2%). The length of the consensus varies between 6,000 and 8,000 bp, depending on the number of monomer repeats in the promoter region. The number of full-length (FL) elements varied greatly between families as FL elements belonging to older families tend to be less numerous in comparisons to younger families. This is expected as L1 inserts decay over time because of internal deletions. The copy number of a few older families was too low (<10 copies) to derive accurate FL consensus sequences. Such families were removed from the dataset as we maintained a strict rule of using only FL elements, that is elements with intact 5’UTR, ORF1, ORF2, and 3’UTR. Thus our dataset represents relatively high copy number families which have inserted in the mouse genome since the split between mouse and rat, about 13 MY ago [53]. It is very likely that additional ancient, small copy number families exist but were missed by our approach.

**Table 1:** **Copy number**, **divergence, and age of mouse L1 families**

**Family**^**a**^	**Repeat masker classification**	**Promoter type**	**LPR structure**	**Genomic copy number**^**b**^	**Number of FL elements**	**Average pairwise divergence (% ± S**.**E.)**^**c**^	**Age (Myr)**^**d**^
L1MdA_I	L1MdA	A	66-42	4,249	1,620	0.376 ± 0.073	0.21 (0.17-0.25)
L1MdA_ II	L1MdA	A	66-42-42	5,156	1,240	2.939 ± 0.294	1.62 (1.45-1.78)
L1MdA_ III	L1MdA	A	66-42-42	4,337	606	3.916 ± 0.304	2.15 (1.99-2.32)
L1MdA_IV	L1MdF2	A	66-42-42	1,209	645	4.346 ± 0.414	2.39 (2.16-2.62)
L1MdA_V	L1MdF3	A	66-42-42	945	299	5.167 ± 0.341	2.84 (2.65-3.03)
L1MdA_VI	L1MdF3	A	66-66	5,497	219	8.554 ± 0.434	4.70 (4.47-4.94)
L1MdA_VII	L1MdF2	A	66-66	5,684	759	8.346 ± 0.414	4.59 (4.36-4.82)
Tf_I	L1Md_T	F	66-42-42	5,601	1,593	0.462 ± 0.095	0.25 (0.20-0.31)
Tf_II	L1Md_T	F	66-42-42		1,282	0.496 ± 0.087	0.27 (0.22-0.32)
Tf_III	L1Md_T	F	66-42-42	4,678	1,892	2.233 ± 0.196	1.23 (1.12-1.34)
Gf_I	L1Md_F, L1Md_T	F	66-42-42-42	2,177	615	1.356 ± 0.250	0.75 (0.61-0.88)
Gf_II	L1Md_T	F	66-66-66	770	368	3.929 ± 0.421	2.16 (1.93-2.39)
L1MdF_I	L1MdF2	F	66-42-42	5,112	1,209	3.853 ± 0.278	2.12 (1.97-2.27)
L1MdF_II	L1MdF2	F	66-42-42		609	4.537 ± 0.271	2.50 (2.35-2.64)
L1MdF_III	L1MdF2	F	66-66		548	8.040 ± 0.400	4.42 (4.20-4.64)
L1MdF_IV	L1MdF2	F	66-42-42	6,179	964	11.627 ± 0.503	6.39 (6.12-6.67)
L1MdF_V	L1VL1, L1MdF2	F	66-42	3,936	884	11.683 ± 0.487	6.43 (6.16-6.69)
L1MdF_anc__I	L1Md_F, L1_Mus1	F_anc_	66-42	4,398	418	12.366 ± 0.610	6.80 (6.47-7.14)
L1MdF_anc__II	L1_Mus2	F_anc_	66-66-66	16,491	460	16.795 ± 0.821	9.24 (8.79-9.69)
L1MdN_I	L1VL1, L1Md_F, L1Md_F3	N	66-42-42	2,237	367	3.447 ± 0.212	1.90 (1.78-2.01)
L1MdV_I	L1VL1, L1_Mus1	V	45-66	5,777	318	15.257 ± 0.647	8.39 (8.04-8.75)
L1MdV_II	L1_Mus3	V	66	3,848	470	18.318 ± 0.855	10.07 (9.60-10.55)
L1MdV_III	Lx	V	66-66	NA	N/A	17.575 ± 0.968	9.67 (9.13-10.20)
L1MdMus_I	L1_Mus1	Mus	66-66-42-56	4,947	535	12.068 ± 0.590	6.64 (6.31-6.96)
L1MdMus_II	L1_Mus2	Mus	66-66	1,924	304	14.971 ± 0.521	8.23 (7.95-8.52)
L1Lx_I	L1_Mus3	Lx	66-66	1,649	384	19.864 ± 0.846	10.93 (10.46-11.39)
L1Lx_II	L1_Mus4	Lx	66-66	3,546	186	23.907 ± 0.998	13.15 (12.60-13.70)
L1Lx_III	L1_Mus4	Lx	66-66	3,667	193	18.595 ± 0.841	10.23 (9.76-10.69)
L1Lx_IV	Lx	Lx	66-66	NA	N/A	25.642 ± 1.237	14.10 (13.42-14.78)

^a^Family names based on Repeat Masker database.

^b^The genomic copy number of Tf_I and II and F_I, II, and III were combined due to the small number of diagnostic characters at the 3' end.

^c^Average pairwise divergences were calculated using the maximum composite likelihood method (MEGA 4.0 package).

^d^Dates were calculated assuming a substitution rate of 1.1% / Myr.

### Phylogenetic analysis of L1 families based on ORF2

**Figure 1:**
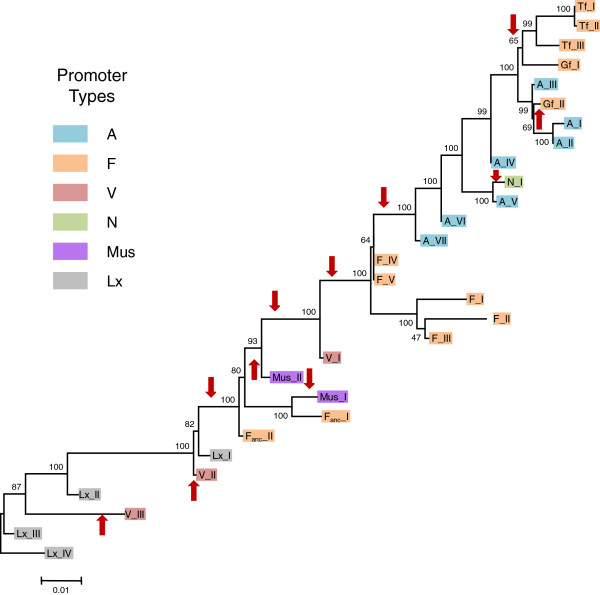
**Phylogenetic tree of mouse L1 families based on the longest non**-**recombining region of ORF2**, **including the reverse transcriptase domain.** This segment corresponds to the region between nucleotide 2095 and 4489 on the alignment provided as supplementary material. The tree was built using the maximum-likelihood method with the HKY+G model. The numbers indicate the percentage of time the labeled node was present in 1,000 bootstrap replicates of the data. Red arrows indicate the acquisition of a new 5’UTR.

As L1 families have extensively recombined with each other (see below), various regions of L1 yield different evolutionary histories and it is impossible to build a single phylogenetic tree based on the entire length of the element. Figure 1 shows the tree built using the longest non-recombining segment of ORF2 (2.5Kb). This segment recapitulates the evolutionary history of L1 lineages more faithfully than other regions because it has not recruited older sequences that would have distorted its evolution. In addition, the branching order on this tree is generally consistent with the age of the families (Table 1), so that older families are closer to the base of the tree and younger families appear more derived. The most recently active families, the L1MdA lineage (characterized by an A promoter) and the L1MdTf lineage (characterized by an F promoter), cluster into well supported paraphyletic and monophyletic lineages, respectively. Each of these lineages contains three families, namely L1MdA_I, II, and III and L1MdTf_I, II, and III. We also identified two families that could be classified as L1MdGf, based on similarity with a previously described family [43]. However, these two families (provisionally named L1MdGf_I and II) do not form a monophyletic group as L1MdGf_I appears more related to L1MdTf and L1MdGf_II groups with L1MdA families. The branch leading to this group of active and recently active families is composed of four families with an A promoter (L1MdA_IV to VII) and the only family carrying the N promoter (L1MdN_I). These families evolved from a group of sequences carrying an F promoter (L1MdF_IV and V). Families L1MdF_I, II, and III constitute a lineage that evolved independently and in parallel with the main A lineage. The F lineage possibly evolved from a family which was carrying a V promoter and which appears to be the last active family with this promoter type. This family in turn evolved from a family carrying the Mus promoter, which apparently evolved from a family carrying the F_anc_ promoter (L1MdF_anc__II). At the same time two families branched independently from the main lineage, one carrying a Mus promoter (L1MdMus_I) the other one the F_anc_ promoter (L1MdF_anc__I). Preceding the L1MdF_anc__II family a lineage made of four families with an Lx promoter was active. At two points in time the Lx promoter was replaced by the V promoter (yielding L1MdV_II and III) but these families did not persist or produce novel lineages.

One of the most striking features visible on the tree is that families with similar 5’UTRs do not form monophyletic groups indicating that L1 families have frequently recruited novel 5’UTR, either from unknown sources or from ancient families. The oldest families in our study carried an Lx promoter, which was replaced three times: once by the F_anc_ promoter (L1MdF_anc__II) and by the V promoter twice (L1MdV_II and III). The F_anc_ promoter was replaced independently twice by the Mus promoter as L1MdMus_I and L1MdMus_II do not form a monophyletic group. The Mus promoter was eventually replaced by the V promoter (L1MdV_I) and went extinct. The F promoter was then resuscitated approximately 6.4 MY ago and gave rise to families L1MdF_I to V. Approximately 4.6 MY ago the A promoter was recruited yielding the modern A lineage which extend from families L1MdA_VII to I. Within this lineage, an additional recruitment occurred resulting in the L1MdN_I family. Finally the F promoter was recently recruited twice, approximately 2.2 MY by the L1MdGf_II family and approximately 1.2 MY by the Tf/Gf_I lineage. Thus we estimate that L1 in mouse has experienced 11 replacements of 5’UTR.

The topology of the ORF2 tree indicates that mouse L1 families evolved mostly as a single lineage. This does not mean that a single family or single lineage was active at a time. In fact, the co-existence of multiple active families characterizes the evolution of L1 for the last 13MY of mouse evolution. For instance between 1 and 2.5 MY ago, six families (L1MdTf_III, L1MdA_II, L1MdA_III, L1MdGf_II, LMdN_I, and L1MdF_I) were active in the mouse genome as attested by the overlap in their average pairwise divergence (Table 1). In some cases, several families evolved into lineages that diversified and co-existed with the dominant lineage for several MY. The lineage composed of L1MdF_I, II, and III is the one that co-existed the longest with the lineage that yielded the currently active families. L1MdF_I was active 2.12 MY ago, at about the same time as families L1MdA_III and L1MdN_I. These families, however, are all descendants of family L1MdF_IV which was active 6.4 MY ago (Figure 1 and Table 1). Thus the lineage consisting of L1MdF_I, II, and III co-existed with the lineage that produced L1MdA_III and L1MdN_I for more than 4 MY. Eventually the L1MdF lineage became extinct. Thus the cascade structure of the ORF2 tree, typical of the single lineage mode of evolution reported in other mammals, is consistent with a model in which multiple families are concurrently active until one of them attains replicative supremacy, coinciding with the extinction of its competitors.

### Detection of recombination among murine L1 families

Because L1 families have frequently recruited novel promoters we decided to examine if L1 lineages have exchanged genetic information in other regions of the element. To this end, several methods implemented in the RDP 3.0 software were used: two substitution-based approaches, MaxChi [54] and Chimera [55], and two phylogenetic approaches, Bootscan [56] and RDP [57]. Breakpoints and statistically significant events of genetic recombination detected by RDP were verified by visual inspection of the FL consensus alignment (see Additional file 3) and phylogenetic analyses. A minimum of six recombination events was detected.

**Figure 2:**
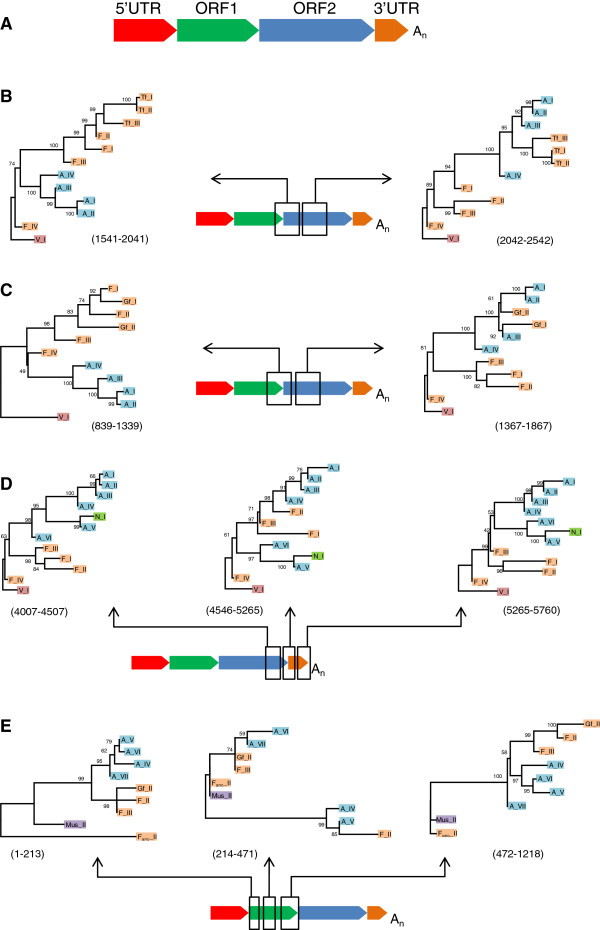
**Evidence for recombination between mouse L1 families.** (**A**) Schematic structure of an L1 element; (**B**) Recombinant origin of the Tf families; (**C**) Independent recombinant origin of the Gf_I and Gf_II families; (**D**) Evidence for recombination at the ORF2-3’UTR junction; (**E**) Evidence for the transfer of the coiled coil domain from Mus_II to A_VI, A_VII, F_III, and Gf_II. The numbers in parentheses correspond to the position of the fragments used to build the tree relative to the alignment provided as supplementary material and beginning at position 1 of ORF1.

Starting with the most recent events, the L1MdTf and L1MdGf families were the result of three independent recombination events between L1MdA_III and L1MdF families. Analyses of non-recombinant segments spanning ORF1 and the 5’ end of ORF2 indicate that both Tf (Figure 2B) and Gf (Figure 2C) families are nested within the more ancestral L1MdF lineage. However, the topology derived from the region spanning the central section of ORF2 suggests that Tf and Gf are decendants from the L1MdA family. The recombination events that produced these families occurred independently as the recombination breakpoints are different. The breakpoint for the two Gf families lies towards the 5’ end of ORF2, but are approximately 30 bp apart (see Additional files), reflecting two independent events of recombination supported by the considerable number of differences between L1MdGf_I and L1MdGf_II in ORF1 (see below). Based on differences in ORF1 we determined that L1MdGf_II could result from a recombination event between L1MdF_III and L1MdA_III and L1MdGf_I from recombination between L1MdF_I or II and L1MdA_III. The three L1MdTf families result from recombination between L1MdF_II and L1MdA_III, but the breakpoint for the Tf families is located approximately 700 bp downstream from the breakpoints detected in the Gf families. This breakpoint is shared among the three Tf families suggesting the recombination event occurred at the origin of the Tf lineage.

The next oldest recombination event is between the ancestor of L1MdA_IV (which is the ancestor of L1MdA_I, II, and III) and L1MdF_II, near the 3’ end of the element (Figure 2D). A 666 bp region was transferred from L1MdF_II to the L1MdA_IV family. This fragment is also found in all L1MdA sequences derived from L1MdA_IV as well as the Gf and Tf families since they also acquired their ORF2 and 3’UTR from an ancestral L1MdA family. Similarly, a segment located in the coiled-coil domain of ORF1 was transferred from L1MdMus_II to L1MdA_VII and L1MdA_VI (Figure 2E). Subsequently an overlapping region was transferred from L1MdA_VII or L1MdA_VI to L1MdF_III. This segment is also found in L1MdGf_II as this family got its ORF1 from L1MdF_III.

It should be noted that our criteria for identifying recombination events were stringent, as we only considered the recombination of large segments to be significant. Thus it is plausible that exchanges of sequences of shorter length have occurred between L1 families but were not detected due to the small number of defining characters in some conserved regions of L1, such as ORF2. The number of recombination events reported here suggests that recombination has played a significant role in the evolution of novel L1 families in mouse and can occur across the entire length of L1.

**Figure 3:**
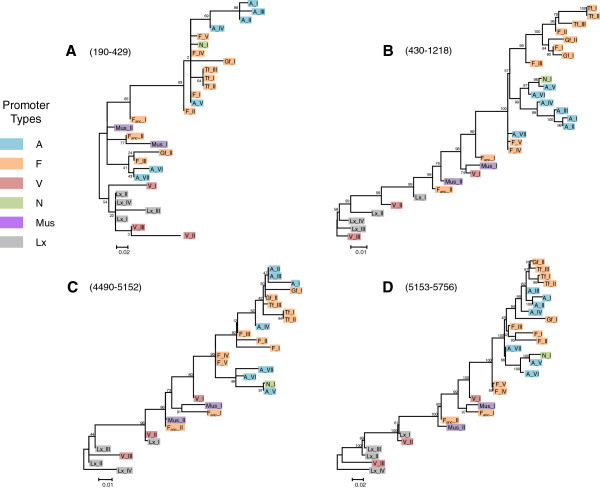
**Phylogenetic trees of mouse L1 families based on (A) the coiled coil domain, (B) the 3’ end of ORF1 and the 5’ terminus of ORF2, (C) the 3’ terminus of ORF2 and the 5’ end of the 3’UTR and (D) the 3’ terminus of the 3’UTR.** The trees were built with the maximum-likelihood method using the JC (tree **A**), TN93+G (**B**), HKY+G (tree **C**) or T92 (tree **D**) models. The numbers indicate the percentage of time the labeled node was present in 1,000 bootstrap replicates of the data. The numbers in parentheses correspond to the position of the fragments used to build the tree relative to the alignment provided as supplementary material and beginning at position 1 of ORF1.

The exchange of genetic information between families constitutes a significant challenge for evolutionary analyses as most phylogenetic algorithms do not allow for recombination. Thus we performed phylogenetic analyses using regions of L1 delimited by recombination breakpoints to fully assess the impact of recombination on the evolutionary history of FL L1 elements (Figure 3). Trees A and B are based on the coiled coil domain of ORF1 and the 3’ half of ORF1 through the 5’ end of ORF2, respectively. The main difference between the ORF2 tree and tree B is that recently active families with similar 5’UTRs form monophyletic groups: families L1MdA_I to VI cluster together and families L1MdF_I, II, and III, Tf_I, II, and III, and Gf_I and II group together (tree B on Figure 3). Further upstream in the coiled coil domain (tree A on Figure 3) this monophyly vanishes because of the transfer of the coiled-coil motif from L1MdMus_II to L1MdA_VI, L1MdA_VII, L1MdGf_II, and L1MdF_III. Tree C is based on the 3’ terminus of ORF2 and the 5’ end of the 3’ UTR. The main difference with the ORF2 tree is the position of all families that are descendant of families L1MdA_IV (that is L1MdA_I to III, the Tf, and the Gf families). These families appear closer to families L1MdF_I to III than to families L1MdA_V to VII because of the transfer of this segment from L1MdF_II to L1MdA_IV. Further downstream, the tree based on the 3’ terminus of L1 (tree D) lacks resolution because of the length of the sequence analyzed and the small number of characters differentiating the families. The main difference with tree C is the position of family L1MdGf_II which branch outside a monophyletic group composed of families L1MdTf, L1MdGf_I, and L1MdA_I to IV, consistent with the independent origin of this recombining family.

### Evolution of the ORFs

We then examined the evolution of the protein coding sequences encoded in L1, ORF1, and ORF2. ORF2 is the most conserved region of L1. There are very few amino acid changes, in particular in the endonuclease and reverse transcriptase domains which are functionally indispensable [21,58]. All the methods we used to assess the impact of selection on ORF2 indicate that this region is evolving under strong purifying selection, that is selection against amino acid changes (Table 2). We analyzed separately the 5’ and 3’ termini of ORF2 because of the presence of recombination. In both regions, the PARRIS methods found no evidence that a subset of amino-acid is evolving under positive selection and estimated a mean dN/dS of 0.308 and 0.229, for the 5’ and 3’ termini, respectively. Similarly, the values of dN/dS estimated by the GABranch method were all significantly lower than 1. In addition, two of the three methods used to detect selection at specific amino acid (SLAC and REL) failed to find evidence of positive selection, although they identified a large number of amino acid under negative selection (not shown). The FEL method identified two amino acids that could have evolved under positive selection but as these two residues have not been recovered by the two other methods, it is likely they constitute false-positives.

**Table 2:** Summary of selection detection tests

		**PARRIS**	**GABranch**	**Positively selected sites**		
**ORF**	**Regions**	**Mean dN**/**dS**	**Number of branches with positive selection**	**SLAC**	**FEL**	**REL**
ORF1	5' terminus	0.494 ± 0.275	0	0	0	0
	Coiled coil	0.608 ± 0.401	0	0	0	8,089
	3' terminus	0.354 ± 0.371	0	0	0	348,351
ORF2	5' terminus (1–1,170)	0.308 ± 0.411	0	0	0	0
	3' terminus (1171-end)	0.229 ± 0.353	0	0	445,945	0

We examined the level of conservation of domains of ORF1 that are known to be functionally important [19,59,60]. Three domains have been identified: a coiled coil (CC) domain that mediate the formation of ORF1p trimers, a RNA-recognition motif (RRM), and a C-terminal domain (CTD). The 3’ half of ORF1, which contains the RRM and CTD domains, as well as approximately the first 50 amino acids of ORF1 are very conserved across families, in contrast with the CC domain that shows a high level of structural variation. We analyzed independently the 5’ terminus, the CC domain, and the 3’ half of ORF1 for evidence of selection using recombination breakpoints as boundaries. All the methods used strongly indicated that the 5’ terminus and the 3’ half of ORF1 are evolving under purifying selection. The PARRIS method rejected the hypothesis that a subset of amino acid is evolving under positive selection and the GABranch method showed that dN/dS has remained significantly lower than 1 in these regions during the entire evolutionary span covered by the analysis. This is not surprising, especially for the 3’ half of ORF1, as the RRM and CTD motifs were shown to be conserved across mammals [60]. The SLAC, FEL, and REL programs failed to identify a single amino acid under positive selection at the 5’ end. In 3’, the REL method identified two amino acids under positive selection but these residues are likely to be false-positive as the changes in amino acid result from independent events of mutation at CpG nucleotides, which are known for their unusually high mutation rate.

More surprising is the degree of conservation at the amino acid level of the CC domain. Previous studies have shown that the CC domain of ORF1 has evolved under positive selection in primates [30,39]. In the case of the mouse, surprisingly, the PARRIS method rejected the hypothesis that some amino acid evolved under positive selection, although a moderately high dN/dS ratio was obtained (0.608), and the GA Branch method failed to identify a single branch in the evolution of the coiled coil with a dN/dS >1. Out of the three methods (SLAC, FEL, and REL) used to detect selection at specific amino acids, only one (REL) identified two amino acids that could have evolved under positive selection. It is thus plausible that these two sites are false-positive as they have been identified by a single method. Even if these sites are evolving under positive selection, it remains true that the signature of positive selection in the mouse CC is much weaker than it is in human [30,39].

**Figure 4:**
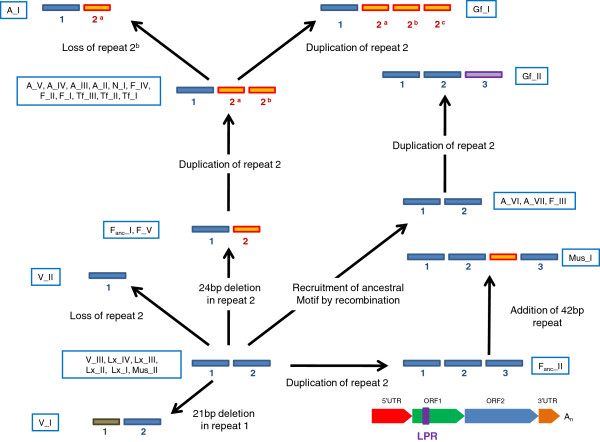
**Evolution of the length polymorphic region of ORF1 in mouse.** The blue boxes correspond to the 66 bp motifs and the orange box, the 42 bp motifs. The position of the polymorphic region on a full-length L1 element is displayed in the bottom right of the figure.

Although the CC domain is relatively conserved at the amino acid level, it shows a high level of structural variation. Previous studies have identified a region called length polymorphism region (LPR) [33,61]. Using our FL consensus alignments we were able to reconstruct the complex history of this region (depicted on Figure 4). The ancestral state is found in the oldest families (Lx_I, Lx_II, Lx_III, Lx_IV, and L1MdMus_II) and contains two 66 bp repeats. From this ancestral motif, four independent modifications have occurred: the loss of the second 66 bp repeat in L1MdV_II, a 21 bp deletion in the first 66 bp repeat found in the L1MdV_I family, a duplication of the second repeat resulting in three 66 bp repeats in L1MdF_anc__II and a 24 bp deletion in the second repeat found in L1MdF_anc__I and L1MdF_IV. The 66–42 bp motif was followed by a duplication of the 42 bp unit resulting in a 66-42-42 bp structure which is found in families L1MdA_V to II, L1MdN_I, L1MdTf_III to I, and L1MdF_I, II, and V. This motif further evolved by the loss of the second 42 bp repeats in L1MdA_I and L1MdF_IV and by the addition of a third 42 bp unit in family L1MdGf_I. The ancestral 66–66 bp motif was recruited by recombination in families L1MdF_III, L1MdA_VI, and VII, and acquired a third 66 bp unit in family L1MdGf_II. These structural changes in the LPR resulted in changes in the length and structure of the CC. Coiled coils are formed from two or more α-helical peptide chains that contain a distinct arrangement of non-polar side chains. Domains that can form CC consist of heptads (or seven residue repeats) with non-polar or hydrophobic residues in the first and fourth positions [62]. The CC in L1 plays an important role in holding together the dumbbell-shape ORF1p trimers [18]. The shortest CC domain is 66 amino acids long and contains seven heptads (based on predictions using the program COILS) in family L1MdV_I. The longest CC is 111 amino acids long and contains 12 heptads in family L1MdGf_I. Between these two extremes, families with 8, 10, and 11 heptads were found.

### Evidence for the lateral transfer of L1 families

**Figure 5:**
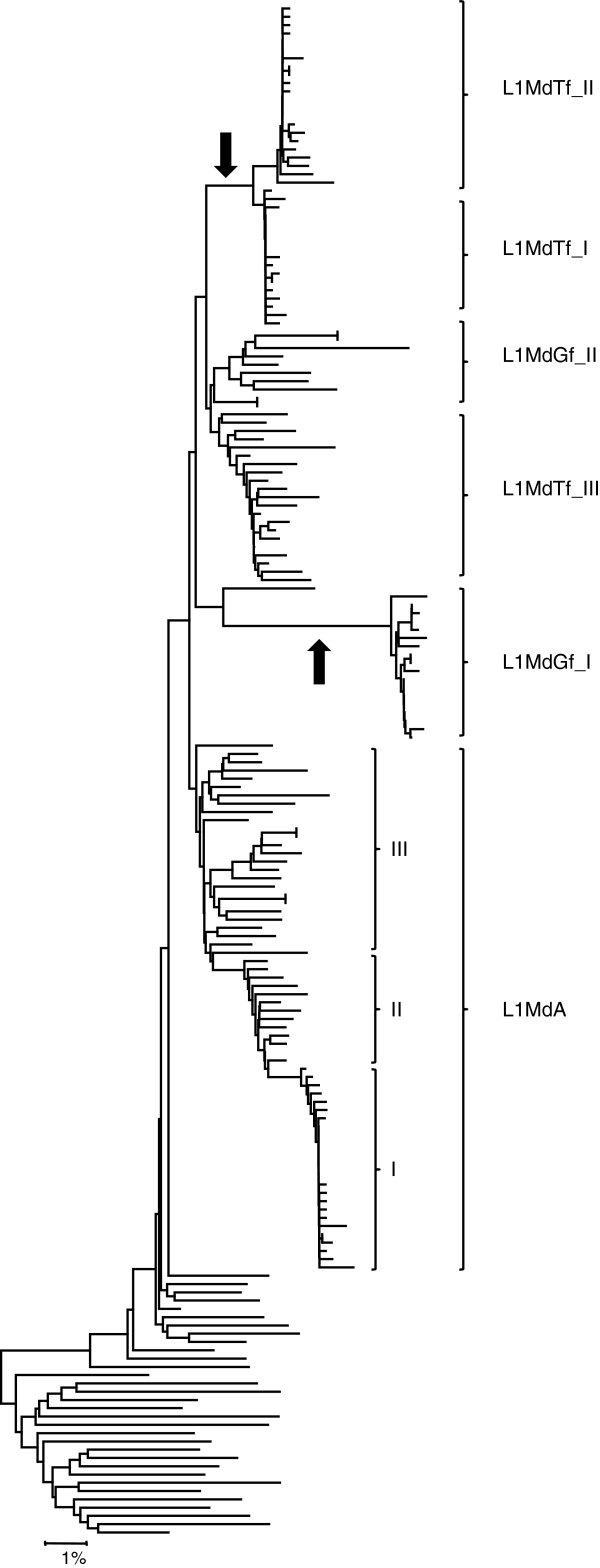
**Phylogeny of genomic copies showing lateral transfer of the L1MdTf**_**I**, **L1MdTf**_**II, and L1MdGf**_**I families.** The tree was built using the neighbor joining method based on Kimura 2-parameters distance. The long branches suggestive of lateral transfers are indicated with black arrows. In contrast the L1MdGf_II and L1MdTf_III families, as well as the three L1MdA families, are not separated from other sequences by long branches indicating they have evolved from older families within the mouse genome. The sequences used to build this tree were randomly chosen within each of the recently active families in the mouse genome. When other sequences are selected, the topology of the tree remains the same.

Finally, we examined the possibility of lateral transfer in the evolution of murine L1. In mammals, L1 is transmitted vertically and there is no evidence of lateral transfer [63], except in case of inter-specific hybridization. Inter-specific hybridization had previously been described among mice of the genus *Mus* and it has been proposed that some L1 families in the house mouse genome were acquired by hybridization [44,64,65]. In order to detect hybridization we used a phylogenetic approach: if a L1 family is invading a genome through hybridization, long branches might be expected with a lack of intermediate sequence on a tree built using genomic copies. In contrast, under the strict vertical mode of transmission, intermediate sequences would be expected between all families. We built a tree using the 3’ UTR of a large number of genomic copies representative of the most recently active families (Figure 5). Two cases of long branches with no intermediate sequences were found: one leading to the L1MdTf_I and II families, and the other leading to L1MdGf_I. This analysis suggests that the L1MdGf_II and L1MdTf_III families evolved within the house mouse genome but that the L1MdTf_I and II and the L1MdGf_I families were acquired through inter-specific hybridization. We can also infer that these transfers resulted from two independent hybridization events since the two Tf families amplified approximately 0.25 MY ago whereas L1MdGf_I amplified approximately 0.75 MY ago.

## Discussion

We performed the first comprehensive analysis of L1 evolution since the completion of the mouse genome [2]. The analysis is limited to the most recently active L1 families and covers approximately the last 13 MY of mouse evolution. As murine rodents evolve approximately eight times faster than hominoids, the amount of evolutionary change investigated here is similar to previous studies in humans that covered more than 80 MY of primate evolution [30,35]. The results are consistent with the large number of analyses performed in the pre-genomic era [32,33,41-45,50,65-68] but, by focusing solely on intact FL elements, we were able to provide for the first time a complete picture of the evolution of mouse L1 families over the entire length of the element.

### Evolution of L1 as a single lineage

The evolution of L1 in mouse fits the single lineage mode of evolution described previously in other mammals and particularly in human [30,35,63,69]. This is exemplified by the similarity between the tree in Figure 1 and the tree based on the human ORF2 (Figure 2 in [30]). This model is based on the observation that L1 phylogenies have a typical cascade structure that is best explained by the successive activity of L1 families: a single family, or a group of closely related families, is active at a given point in time until a new family emerges and replaces the pre-existing family, which usually becomes extinct. In some instances, however, several lineages may co-exist until one eventually becomes extinct. This is the case of the L1MdF_I, II, and III lineage which co-existed with the dominant lineage for approximately 4 MY and of the Tf and L1MdA_I, II, and III lineages that co-existed for about 2 MY and are still active in the mouse genome. In ancestral primates a similar situation occurred but on a much longer period of evolutionary time as the L1PB and L1PA lineages co-existed for 30 MY [30]. We previously observed that, in human, L1 lineages that co-exist for extended periods always have different promoter sequences. We proposed that families with different promoter sequences rely on different host-factors for their transcription and are consequently not relying on the same host-encoded resources [30]. This situation allows them to co-exist as they are not using the same genomic ‘niche’. In mouse the same observation can be made. The lineage composed of L1MdF_I, II, and III co-existed with the main lineage when this one was dominated by families carrying the A promoter (L1MdA_III to VI). Similarly, the two lineages that are currently active, the L1MdA_I, II, and III and the L1MdTf/Gf, carry different, non-homologous 5’UTRs. Thus, it is possible that the conditions that allow for multiple lineages to co-exist are the same in mouse and in human. Unlike in modern human where a single family is currently active (the Ta family) [28], the modern house mouse genome harbors several families with different 5’ UTR and consequently present an excellent model to test experimentally the hypothesis that the activity of different 5’UTR is one of the conditions for the co-existence of families and lineages.

### Acquisition and exchange of sequence during L1 evolution

The analysis of FL elements has revealed the extraordinary ability of L1 families to acquire novel motifs and to exchange sequences (Figures 2 and 3). The recruitment of novel 5’UTR sequences [30,33] as well as the recombinant nature of some L1 families in mouse [45,46] and rat [34,69,70] have long been described. Three mechanisms have been proposed to account for the mosaic nature of some families. First, recombination between genomic copies, that is at the level of DNA templates, could result in the formation of a novel transpositionally competent family. This hypothesis has been discounted on the basis that it is highly unlikely that a chance recombination event between two replicatively competent elements occurred while recombination between any of the hundreds of thousands L1 pseudogenes, the majority of which have suffered the effect of inactivating mutations, is much more likely to produce an inactive element [69]. Second, recombination could occur at the time the L1 RNA is reverse-transcribed and could result from the formation of a RNA/DNA heteroduplex between the L1 RNA and a genomic copy at the insertion site [71]. This model is supported by the observation that the recruitment of novel motifs seems to be directional as it is always a chronologically young 3’ end that recruits an older 5’ terminus [69]. Third, mosaic elements could be produced if the L1 encoded reverse transcriptase switches RNA strand at the time of insertion. Polymerase strand-switching is a well-known feature of RNA viruses [72,73]. This mechanism insures that recombination occurs between replicatively competent elements, that is elements that carry a 5’UTR capable of driving their transcription. The third model predicts that recombination occurs only between families that are simultaneously active whereas the first and second models do not have such a requirement. We found that the exchange of genetic information occurs both between simultaneously active families and by resuscitation of motifs from extinct families. For instance, the coiled-coil domain of L1MdMus_II has been recruited by L1MdA_VII about 4.6 MY ago, long after the extinction of L1MdMus_II which was active 8.23 MY ago. The L1MdGf_II family is also the product of a recombination between two families that were not active simultaneously, the L1MdF_III and the L1MdA_III families (which amplified 4.42 and 2.15 MY ago, respectively). All other instances of recombination occurred between families that were simultaneously active, which is consistent with the polymerase strand-switching model. Similarly, the acquisition of novel 5’UTRs tend to result from the transfer of 5’ termini between families that were active at the same time. This is exemplified by the evolution of the F-type which was transferred from L1MdF_anc__I (active 6.80 MY ago) to the ancestor of L1MdF_V (at 6.43 MY) and subsequently transferred from L1MdF_I (active 2.12 MY ago) to the recently active L1MdTf and L1MdGf families.

### Evolution of ORF1

The first ORF is arguably the least understood region of L1, although it has been the subject of much attention in the past few years [17-20,59,60,74-78]. Its secondary structure has been resolved as a dumbbell shape resulting from the formation of a trimeric structure mediated by the coiled coil domain [18]. It is established that it has RNA-binding abilities, mediated by the RRM, can act as a nucleic acid chaperone [19,20] and form multimers in the presence of nucleic acids [78]. Previous studies have shown that the 3’ half of ORF1 is very conserved [60] and our analysis confirms this is the case in mouse. In contrast, studies in human have demonstrated that the coiled-coil domain is evolving under strong positive selection as indicated by the high values of dN/dS reported in the evolution of this region [30,39]. Such a rapid evolution at the amino-acid level is certainly adaptive and it was proposed that this was the result of an arms-race between L1 and its human host. This hypothesis was further supported by the fact that periods of adaptive evolution in the coiled coil coincide with period of intense L1 activity [30]. However, we failed to find strong evidence of adaptive evolution in the mouse coiled coil. In contrast we found an extraordinary level of structural instability in this region (Figure 4), unexpected in a protein coding region critical for the multimeric structure of the functional protein. Instability in this region has also been described in the rat L1 suggesting a common role for these structural changes in these two species [34,69]. Structural changes in the coiled coil occur so frequently that it is tempting to speculate that they are adaptive, and are evolutionarily equivalent to periods of intense amino acid replacement in humans.

## Conclusions

We performed a comprehensive analysis of L1 evolution in mouse. This analysis covered the last 13 MY of mouse evolution, since the split between mouse and rat. The mouse L1 has evolved as a single lineage for most of its evolution, although co-existence between families carrying different promoter sequences was observed. L1 families have frequently acquired novel 5’UTR and have exchanged sequences over the entire length of the element. No evidence of rapid amino acid replacement in the ORF1 was detected, although it is likely that the structural instability of the CC domain is adaptive. The general pattern of evolution of mouse L1 is similar to the one in human suggesting that the nature of the interactions between L1 and its host might be similar in these two species. There are however some intriguing differences between mouse and human, particularly in the evolution of ORF1. These differences suggest that the molecular mechanisms involved in host-L1 interactions might be different in these two species.

## Methods

### Collection and classification of full-length L1 elements

Full-length (FL) elements were collected from the *Mus musculus* 2006 (mm8) genome built using the GPS [79]. GPS conducted a BLAST type-search (WU-tBLASTn) of the genome using the conserved Reverse Transcriptase (RT) domain of ORF2 as a query. GPS then cut 7,000 bp upstream and downstream of the RT domain yielding a 14,000 bp fragment. A second WU-tBLASTn was then performed on the 14,000 bp cutouts to identify regions characteristic of L1 (ORF1, the endonuclease domain of ORF2, the RT domain, and the 3’UTR). In this analysis, GPS did not search for sequence identity at the 5’ end since L1 is known to frequently recruit novel sequences as 5’UTR [30,33]. Thus, a file containing 3,000 bp upstream of ORF1 was generated for further analyses. The FL sequences were first sorted based on their 5’UTRs. Once elements were sorted based on their 5’UTRs, they were further categorized into families using a phylogenetic analysis of the 3’ terminus. A family is defined as a collection of elements that result from the activity of a highly homogenous group of progenitors, which are characterized by a unique combination of characters. In the first step of the phylogenetic analysis, neighbor joining trees [52] of elements sharing similar 5’UTRs were built. Distinct clusters were provisionally considered families and were validated by a second round of phylogenetic analysis based on the principle that elements belonging to the same family should yield a star phylogeny because they result from the activity of similar progenitors. These families were further confirmed by phylogenetic analysis performed on other regions of L1 to verify that the homogeneity of the families extend over the entire length of the element. Full-length consensus sequences were derived for each family and are available on Repbase. Phylogenetic analyses were performed using the neighbor joining (NJ) method [52] based on the maximum composite likelihood parameters distance included in the MEGA 5.01 software package [80]. The model that best fits the data was determined for each alignment using MEGA. The robustness of each phylogenetic tree was assessed using a bootstrap procedure with 1,000 replicates. Families were named by the name of the 5’ promoter (A, F, F_anc_, V, Lx, Mus, or N; see result) followed by a roman number. The smaller the roman number, the younger the family is. For instance families L1MdA_I, L1MdA_II, and L1MdA_III are subsets of the previously described L1MdA family; family L1MdA_I is younger than family L1MdA_II and family L1MdA_III is the oldest of the three. We kept the Gf [43] and Tf [42] names for the recently active Tf and Gf families because these names have been widely used in the literature.

### Analysis of FL elements

NJ, maximum parsimony (MP), and maximum likelihood (ML) trees were calculated for each region of L1. Phylogenetic trees were reconstructed using the MEGA 5.01 package [80]. The RDP3.0 program (Recombination Detection Program 3.0, available at http://darwin.uvigo.es/rdp/rdp.html) was used to search for evidence of recombination among families. RDP allows for the use of several recombination detection methods including substitution and phylogeny-based methods. Two substitution-based methods, MaxChi [54] and Chimaera [55], as well as a phylogenetic method, bootscan [56], were used to analyze the datasets. The RDP software also includes its own unique algorithm termed ‘RDP’ [57] which is also a phylogenetic approach to detecting recombination. A window size of 50 bp was used to detect breakpoints between consensus sequences. Statistically significant events of recombination were verified by comparing phylogenetic trees on each side of the putative breakpoint.

To test for evidence of selection in the evolution of L1 several methods implemented in the web server http://www.datamonkey.com[81] of the HyPhy program [82] were used. The first method uses a maximum likelihood approach (PARRIS) to determine if a proportion of site in an alignment evolves with a ratio dN/dS>1 [83]. A ratio significantly >1 is indicative of positive selection whereas a ratio <1 is indicative of purifying selection. The second method, GABranch [84] can detect lineage-specific variation in selective pressure and requires no *a priori* specification of branches in a phylogeny that may have evolved under different values of dN/dS. The dN/dS test is however not very sensitive, particularly if selection acts on a few codons. For this reason we used three methods designed to detect the action of positive or negative selection at specific sites in an alignment: Single Likelihood Ancestor Counting (SLAC), a Random Effects Likelihood (REL), and Fixed Effects Likelihood (FEL) [85]. For each dataset, the model that best fits the data was determined using the tool available at datamonkey.com. As selection detection methods are sensitive to recombination, we performed our analyses independently for each segment of L1 flanked by recombination breakpoint. Previous studies on human L1 have documented positive selection in the coiled-coil (CC) domain of ORF1 [30,39]. CC structures are formed from two or more α-helical peptide chains that contain a distinct arrangement of non-polar side chains [62]. Domains that can form CC consist of heptads (or seven residue repeats) with non-polar or hydrophobic residues in the first and fourth positions. The program COILS [62] was used to identify the position of the CC domain in each consensus sequence as well as the number of constitutive heptads.

### Age and copy number of L1 families

The age of each subfamily was estimated by calculating the average pairwise divergence based on the 3’UTR. CpG dinucleotides and the highly mutable polypurine tract located in the 3’UTR were removed from alignment. The average divergence between copies as well as the standard error was calculated using the maximum likelihood parameter distance (using the MEGA 5.01 software). Divergences were converted to time assuming a neutral rodent genomic substitution rate of 1.1%/MY (calculated using the data presented on Table 5 of [86] and assuming a divergence *Mus*/*Rattus* at 13MY [53]).

### Availability of supporting data

The consensus sequences are available in Repbase (http://www.girinst.org/repbase/).

## Abbreviations

CC: Coiled coil; FL: Full length; LINE-1 L1: Long Interspersed Nuclear Elements-1; MY: Million of year; ORF: Open reading frame; UTR: Untranslated region.

## Competing interests

The authors declare they have no competing interests.

## Authors’ contributions

AS collected data, performed alignments, analyzed the sequences evolutionarily, and wrote an early draft of the manuscript. CH and MM collected the data using GPS and provided editorial suggestions. SB designed the research and wrote the paper. All authors read and approved the final manuscript.

## Supplementary Material

Additional file 1 **Alignment of mouse L1 consensus sequences starting at the beginning of ORF1.** ORF1 spans positions 1 to 1,218 and ORF2 spans positions 1,262 to 5,096. Click here for file

Additional file 2 **Matrix of pairwise divergence based on the longest non-recombining fragment of ORF2 (from position 2,085 to 4,489 in Additional file 1).** Click here for file

Additional file 3 **Alignments showing recombination break-points among L1 families.** Only the parsimony-informative sites are shown. Click here for file
