# Supplementary material for: Revisiting the evolution of mouse LINE-1 in the genomic era
Source: Mob DNA. 2013 Jan 3;4:3. doi: 10.1186/1759-8753-4-3 (PMC3600994; doi:10.1186/1759-8753-4-3)
Supplement: Additional file 1 — Alignment of mouse L1 consensus sequences starting at the beginning of ORF1. ORF1 spans positions 1 to 1,218 and ORF2 spans positions 1,262 to 5,096. [file 1759-8753-4-3-S1.pdf]

```
#L1MdA_I       ATGGCGAAAG GCAAACGTAA GAATCCTACT AACAGAAATC AAGACCACTC ACCATCATCA GAACGCAGCA CTCCCACCCC  [  80]
#L1MdA_II      .......... .......... .......... .......... .......... .......... .......... ..........  [  80]
#L1MdA_III     .......... .......... .......... .......... .......... .......... .......... ..........  [  80]
#L1MdA_IV      .......... .......... .......... .......... .......... .......... .......... ..........  [  80]
#L1MdGf_I      .......... .T........ .......... .......GC. .G........ .......... ....C...A. .G.....TT.  [  80]
#L1MdGf_II     .......... .T........ .....T.... ........C. .G...T.... .......... ....C..... .......TT.  [  80]
#L1MdTf_I      .......... .T.....G.G .....T.... .....G..C. .......... .......C.. ....C..... .A.....TT.  [  80]
#L1MdTf_II     .......... .T.....G.G .....T.... .....G..C. .......... .......C.. ....C..... .A.....TT.  [  80]
#L1MdTf_III    .......... .T.......G .......... .....G..C. .......... .......... ....C..... .......TT.  [  80]
#L1MdA_V       .....A.... .......... .......... .......... ......G... .......... .......... .......G..  [  80]
#L1MdA_VI      .......... .......... .....T.... ........C. .......... .......... .......... ..........  [  80]
#L1MdF_V       .......... .......... .....T.... ........C. .......... .......... .......... ........T.  [  80]
#L1MdF_IV      .......... .......... .....T.... ........C. .......... .......... ....C..... ........T.  [  80]
#L1MdF_I       .......... .T........ .......... ........C. .G........ .......... ....C...A. .G.....TT.  [  80]
#L1MdF_II      .......... .T.......G .....T.... .....G..C. .......... .......... ....C..... .......TT.  [  80]
#L1MdF_III     ....Y..... .T........ .....T.... ........C. .......... .......... ....C..... .......TT.  [  80]
#L1MdN_I       .......... .......... .......... .......... .......... .......... .......... .......G..  [  80]
#L1MdA_VII     .......... .......... .......... .......... .......... .......... .......... ..........  [  80]
#L1MdMus_I     .......... .......... .....T.... ........C. ....A....G GG........ ....C...T. .G......A.  [  80]
#L1MdFanc_I    .......... .......... .....T.... ........C. .......... .......... ....C..... ........T.  [  80]
#L1MdV_I       .....AGG.. ....G..... ...CAGA.GC ........C. ...GTT...T GG........ ....CA.A.T ........AT  [  80]
#L1MdFanc_II   .......... .......... .....T.... ........C. .......... .G........ ....C...T. .D......A.  [  80]
#L1MdMus_II    ..R....... .......W.. .....T.... ........C. .......... GG........ ....C...T. .G..Y...A.  [  80]
#L1MdV_II      .....AGG.. ....G.A... ...CAGA.GC ........C. ...GTT...T GG........ ....CA.A.T ........AT  [  80]
#L1Lx_I        .....D.... .......Y.. .....T...C ........C. ....HY...T GG........ ....C...T. ........A.  [  80]
#L1Lx_III      .......... ....G.AC.. ...CATA.GC ........C. .C.V.T...T GG........ ....H...TT ........A.  [  80]
#L1Lx_II       .......... ....G..C.. ...CYT...C ........C. ...GHT..WT GG.G...... ...VC...TT .N.....MAN  [  80]
#L1Lx_IV       .......R.. ....GG.C.. ...CATA.GC ........C. ...GNT...T GG........ ....C...TT .....G..A.  [  80]
#L1MdV_III     ....T.GG.. ....G.A... ...CATA.GC ........C. ...GTT...T GG........ ....C..ATT ........AT  [  80]

#L1MdA_I       ACCTAGTCCT GGGCACCCCA ACACAACCGA AAATCTAGAC CCAGATTTAA AAACATTTCT CATGATGATG ATAGAGGACA  [ 160]
#L1MdA_II      .......... .......... .......... .......... .......... .......... .......... ..........  [ 160]
#L1MdA_III     .......... .......... .......... .......... .......... .......... .......... ..........  [ 160]
#L1MdA_IV      .......... .......... .......... .......... .......... .......... .......... G.........  [ 160]
#L1MdGf_I      G..C.A...A ..A.....T. .....C.T.. ...GG..... .TG....... ..G...A... .......... G.........  [ 160]
#L1MdGf_II     G..C.....A ........Y. .....C.T.. ...G...... .TG....... ..G...A... .......... G.........  [ 160]
#L1MdTf_I      G..C.....A ...A...... .....C.T.. G..C...... .T........ ..G...A... .......... G.........  [ 160]
#L1MdTf_II     G..C.....A ...A...... .....C.T.. G..C...... .T........ ..G...A... .......... G.........  [ 160]
#L1MdTf_III    G..C.....A .......... .....C.... ...C...... .T........ ..G...A... .......... G.........  [ 160]
#L1MdA_V       .......... .......... .......... ...G...... ..G....... ..G...A... .......... G.........  [ 160]
#L1MdA_VI      ...C...... .......... .....C.... ...GA...T. ..G....... ..G...A... .......... G.........  [ 160]
#L1MdF_V       G..C...... .......... .....C.YR. ...G...... ..G....... ..G...A... .......... G.........  [ 160]
#L1MdF_IV      .G.C...... ...Y...... .....C.... ...G...... .GG....... ..G...A... .......... G.........  [ 160]
#L1MdF_I       G..C.....A ........T. .....C.T.. ...GG..... .TG....... ..G...A... .......... G.........  [ 160]
#L1MdF_II      G..C.....A .......... .....C.... ...C...... .......... ..G...A... .......... G.........  [ 160]
#L1MdF_III     G..C.....A .......... .....C.... ...G...... ..G....... ..G...A... .......... G.........  [ 160]
#L1MdN_I       .......... .......... .......... ...G...... ..G....... ..G...A... .......... G.........  [ 160]
#L1MdA_VII     .......... .......... .....C.... ...G.W.... ..G....... ..G...A... .......... G.........  [ 160]
#L1MdMus_I     .A.G...... ..AT...... .....C.... ...G.A.... G.G....... ..T...A... ......G... G....A..TT  [ 160]
#L1MdFanc_I    .G.C...... ..AT...... .....C.... ...G.A.... T.G....... ..T...A... ......C... G.........  [ 160]
#L1MdV_I       .G.A...... ..AT..A... T....C.A.. ...G.A.... ATG...C... .GT..C.... .......... ..G......T  [ 160]
#L1MdFanc_II   .G.G...... ..AT...... .....C..A. ...G.A...T T.G....... ..T...A... ......C... G.......TT  [ 160]
#L1MdMus_II    .G.G...... ..AT...... .....C.... ...G.A...Y T.G....... ..T...A... ......C... G.......TT  [ 160]
#L1MdV_II      .G.A...... ..AT..A... T....C.A.. ...G.A...T ATG...C... .GT..C.... .......... ..G......T  [ 160]
#L1Lx_I        .G.G...... ..AT.H.... .....C.G.. ...G.A.V.T T.G....... ..T...A... ......C... G.......TT  [ 160]
#L1Lx_III      .G.G.AC... ..TT...... .....C.A.. ...G.A.... T.T....... ..T..CA... .......... ........VT  [ 160]
#L1Lx_II       .G.A.D.... ..AT.H.... .N...C.G.. ...G.A...T T.G....... ..T..YA... H.....V... ........YT  [ 160]
#L1Lx_IV       .GTG..C... ..NT...... .....C.A.. ...G.A.... T.T....... ..T..CA... .......... .........T  [ 160]
#L1MdV_III     .G.A...... ..AT..A... T....C.G.. ...GBA...Y T.G...C... .RT..C.... .....C.... ..V......T  [ 160]

#L1MdA_I       TCAAGAAGGA CTTTCATAAG TCACTTAAAG ATTTACAGGA GAGCACTGCT AAAGAGTTA- ---------- ----------  [ 240]
#L1MdA_II      .......... .......... .......... .A........ .......... .........- ---------- ----------  [ 240]
#L1MdA_III     .......... .......... .......... .A........ .......... .........- ---------- ----------  [ 240]
#L1MdA_IV      .......... .........C .......... .AA....... .......... .........- ---------- ----------  [ 240]
#L1MdGf_I      .A........ A..CA.Y..C .......... .AA....... ..A....... .........- ---------- ----------  [ 240]
#L1MdGf_II     .......... ...AA....C .......... .AA....... ..A....... ...C.AG..G AAGACATTAA AGAGGAAACA  [ 240]
#L1MdTf_I      .......... ....A....A .......... .AA....... ..A....... .........- ---------- ----------  [ 240]
#L1MdTf_II     .......... ....A....A .......... .AA....... ..A....... .........- ---------- ----------  [ 240]
#L1MdTf_III    .......... ....A....A .......... .AA....... ..A....... .........- ---------- ----------  [ 240]
#L1MdA_V       .......... ....A....C .......... .AA....... ..A....... .........- ---------- ----------  [ 240]
#L1MdA_VI      .......... ....A....C .......... .AA....... ..A....... ...C..G..G AAGACCTTAA AGAGGAAGCA  [ 240]
#L1MdF_V       .......... ....A....C .......... .AA....... ..A....... .........- ---------- ----------  [ 240]
#L1MdF_IV      .......... ....A....C .......... .AA....... ..A....... .........- ---------- ----------  [ 240]
#L1MdF_I       .A........ A...A....C .......... .AA....... ..A....... .........- ---------- ----------  [ 240]
#L1MdF_II      .......... ....A....C .......... .AA....... ..A....... .........- ---------- ----------  [ 240]
#L1MdF_III     .......... ....A....C .......... .AA....... ..A....... ...C..G..G AAGACATTAA AGAGGAAGCA  [ 240]
#L1MdN_I       ........A. ....A....C .......... .AA....... ..A....... .........- ---------- ----------  [ 240]
#L1MdA_VII     .......... ....A....C .......... .AA....... ..A...A... ...C..G..G AAGACCTTAA AGAGGAAACA  [ 240]
#L1MdMus_I     .T.......G .A..A....C .......... .AA....... ..A..N.... ...S..G..G AAGCCCTTAA AGAGGAAGCA  [ 240]
#L1MdFanc_I    .........G .W..A....C .......... .AA....... ..A....... ......G..- ---------- ----------  [ 240]
#L1MdV_I       .T......-- ---------- ---------. .AA....... A.A...A.G. ...C..C..G AAGCCTTTAA AGAGGAAACA  [ 240]
#L1MdFanc_II   .T.......G .A..A....C .......... .AA....... ..A....... ...C..G..G AAGNCCTTAA AGAGGAAGCA  [ 240]
#L1MdMus_II    .T.......G .A..A....C ...Y...... .AA.D..R.. D.A....... ...C..G..G AAGTCCTTAA AGAGRAAACA  [ 240]
#L1MdV_II      DT........ AA.AA....C ..C....... .AA....... D.A...ATMC ..WY..G.-- ---------- ----------  [ 240]
#L1Lx_I        .T........ .A..A....Y ..C....... .AA....... ..A...A.N. ...C..G..G AAGYCCTTAA AGAGGAAACA  [ 240]
#L1Lx_III      .T...R.... .A.AA....C ..C....... .AA....A.. ..A...A.G. ...CG.G..G AAGACCTTAA AGAGGAAACA  [ 240]
#L1Lx_II       .T........ .A.AA....C ..C....... .AA....... ..A...A.G. ...C..G..G AAGCCCTTAA AGAGGAAACA  [ 240]
#L1Lx_IV       .T........ .A.AA....C ..C....... .AA....D.. ..A...A.G. ...C..G..G AAGCCCTTAA AGAGGAAACA  [ 240]
#L1MdV_III     .T........ .A.AA....C ..C....... .AA....... ..A...A.G. ...C..M..G AAGCCCTTAA AGAGGAAACA  [ 240]
```

```
#L1MdA_I      --CAGGCTC- TTAAAGAAAA GCAGGAAAAC ACAGCCAAAC AGGT------ ---------- ---------- ----------  [ 320]
#L1MdA_II     --.....C.- .......... .......... .......... ....------ ---------- --------AG AAATCATTAA  [ 320]
#L1MdA_III    --.....C.- .......... .......... .......... ....------ ---------- --------AG AAGTCCTTAA  [ 320]
#L1MdA_IV     --....YC.- .......... A......... ...A...... ....------ ---------- --------AG AAGTCCTTAA  [ 320]
#L1MdGf_I     --..A.TC.- .......... A......... ..T..T...G ..T.------ ---------- --------AC AAGTCCTTAA  [ 320]
#L1MdGf_II    CAA.AATC.C ........TT A......... .T.A...... ....AGAAGA CATTAAAGAG GAAACACAAA AATCCCTTAA  [ 320]
#L1MdTf_I     --..A.TC.- .......... A......... ...AT..... ....------ ---------- --------AG AAGTCCTTAC  [ 320]
#L1MdTf_II    --..A.TC.- .......... A......... ...AT..... ....------ ---------- --------AG AAGTCCTTAC  [ 320]
#L1MdTf_III   --..A.TC.- .......... A......... ...AT..... ....------ ---------- --------AG AAGTCCTTAC  [ 320]
#L1MdA_V      --..A.TC.- .......... A......... ...A...... ....------ ---------- --------AG AAGTCCTTAT  [ 320]
#L1MdA_VI     CAA.AATC.C ........TT .......... ..GA...... ....------ ---------- ---------- ----------  [ 320]
#L1MdF_V      --..A.TC.- .......... A......... ...T...... ....------ ---------- ---------- ----------  [ 320]
#L1MdF_IV     --..A.TC.- .......... A......... ...A.....S ....------ ---------- --------AG AAGTCCTTAA  [ 320]
#L1MdF_I      --..A.TC.- .......... A......... ...A...... ....------ ---------- --------AG AAGTCCTTAT  [ 320]
#L1MdF_II     --..A.TC.- .......... A......... ...AT..... ....------ ---------- --------AG AAGTCCTTAC  [ 320]
#L1MdF_III    CAA.AATC.C ........TT .......... ...A...... ....------ ---------- ---------- ----------  [ 320]
#L1MdN_I      --..A.TC.- .......... .......... ...A...... ....------ ---------- --------AG AAGTCCTTAT  [ 320]
#L1MdA_VII    CAA.AAT..C ........TT .......... ..GA...... ....------ ---------- ---------- ----------  [ 320]
#L1MdMus_I    CAA.AATC.C .C..G...TT A.....G... ..T..T.... ....AGAAG- ---------- ---------- ---TCCTTAA  [ 320]
#L1MdFanc_I   --G.A.TC.- ........DT A......... ...A...... ....------ ---------- ---------- ----------  [ 320]
#L1MdV_I      CAA.AATB.C ...G...GTT A......... ..TA...... ....------ ---------- ---------- ----------  [ 320]
#L1MdFanc_II  CAA.AATC.C .C......TT A......... ..T..T.... ....AGAAGA CCTTAAAGAG GAAACACAAA AATCCCTTAA  [ 320]
#L1MdMus_II   CAA.AATC.C ..D..R..TT A......... ...A...... ....------ ---------- ---------- ----------  [ 320]
#L1MdV_II     ---------- ---------- ---------- ---------- ---------- ---------- ---------- ----------  [ 320]
#L1Lx_I       CAA.AATC.C ........TT AY........ ...A...... ....------ ---------- ---------- ----------  [ 320]
#L1Lx_III     CAA.AATC.C ........TT A......... ...A...... ....------ ---------- ---------- ----------  [ 320]
#L1Lx_II      CAA.AATC.C ........TT AB........ ...AH..... ....------ ---------- ---------- ----------  [ 320]
#L1Lx_IV      VAA.AATC.C ........TT A......... ...AH..... ....------ ---------- ---------- ----------  [ 320]
#L1MdV_III    CAA.AATC.C ........TT A......... ...AT..... ....------ ---------- ---------- ----------  [ 320]

#L1MdA_I      ---------- ---------- ---------- ---------- ---------- ---------- ---------- ----------  [ 400]
#L1MdA_II     AGAAAAACAG GAAAACACAT CCAAACAGGT ---------- ---------- ---------- ---------- ----------  [ 400]
#L1MdA_III    AGAAAAACAG GAAAACACAT CCAAACAGGT ---------- ---------- ---------- ---------- ----------  [ 400]
#L1MdA_IV     AGAAAAACAG GAAAACACAT CCAAACAGGT ---------- ---------- ---------- ---------- ----------  [ 400]
#L1MdGf_I     AGAAAAACAG GAAAACACAA CCAAACAGGT AGAAG----- -------TCC TTATAGAAAA ACAGGAAAAC ACATCCAAAC  [ 400]
#L1MdGf_II    AGAATTGCAG GAAAACACAA CCAAACAGGT ---------- ---------- ---------- ---------- ----------  [ 400]
#L1MdTf_I     AGAAAAAGAG GAAAAAACAT ACAAACAGGT ---------- ---------- ---------- ---------- ----------  [ 400]
#L1MdTf_II    AGAAAAAGAG GAAAAAACAT ACAAACAGGT ---------- ---------- ---------- ---------- ----------  [ 400]
#L1MdTf_III   AGAAAAAGAG GAAAAAACAT ACAAACAGGT ---------- ---------- ---------- ---------- ----------  [ 400]
#L1MdA_V      AGAAAAACAG GAAAACACAT CCAAACAGGT ---------- ---------- ---------- ---------- ----------  [ 400]
#L1MdA_VI     ---------- ---------- ---------- ---------- ---------- ---------- ---------- ----------  [ 400]
#L1MdF_V      ---------- ---------- ---------- ---------- ---------- ---------- ---------- ----------  [ 400]
#L1MdF_IV     AGAAAAACAG GAAAACACAT CCAAACAGGT ---------- ---------- ---------- ---------- ----------  [ 400]
#L1MdF_I      AGAAAAACAG GAAAACACAT CCAAACAGGT ---------- ---------- ---------- ---------- ----------  [ 400]
#L1MdF_II     AGAAAAAGAG GAAAAAACAT CCAAACAGGT ---------- ---------- ---------- ---------- ----------  [ 400]
#L1MdF_III    ---------- ---------- ---------- ---------- ---------- ---------- ---------- ----------  [ 400]
#L1MdN_I      AGAAAAACAG GAAAACACAT CCAAACAGGT ---------- ---------- ---------- ---------- ----------  [ 400]
#L1MdA_VII    ---------- ---------- ---------- ---------- ---------- ---------- ---------- ----------  [ 400]
#L1MdMus_I    AGAACTACAG GCAAACACTG CTAAACAGGT AGAAGAAACA CAAAAATCCC TTAAAGAATT ACAGGAAAAC ACAACCAAAC  [ 400]
#L1MdFanc_I   ---------- ---------- ---------- ---------- ---------- ---------- ---------- ----------  [ 400]
#L1MdV_I      ---------- ---------- ---------- ---------- ---------- ---------- ---------- ----------  [ 400]
#L1MdFanc_II  AGAATTACAG GAAAACACAA CCAAACAGGT ---------- ---------- ---------- ---------- ----------  [ 400]
#L1MdMus_II   ---------- ---------- ---------- ---------- ---------- ---------- ---------- ----------  [ 400]
#L1MdV_II     ---------- ---------- ---------- ---------- ---------- ---------- ---------- ----------  [ 400]
#L1Lx_I       ---------- ---------- ---------- ---------- ---------- ---------- ---------- ----------  [ 400]
#L1Lx_III     ---------- ---------- ---------- ---------- ---------- ---------- ---------- ----------  [ 400]
#L1Lx_II      ---------- ---------- ---------- ---------- ---------- ---------- ---------- ----------  [ 400]
#L1Lx_IV      ---------- ---------- ---------- ---------- ---------- ---------- ---------- ----------  [ 400]
#L1MdV_III    ---------- ---------- ---------- ---------- ---------- ---------- ---------- ----------  [ 400]

#L1MdA_I      ----GATGGA AATGAACAAA ACCATACTAG AACTAAAAGG GGAAGTAGAC ACAATAAAGA AAACCCAAAG CGAGGCAACG  [ 480]
#L1MdA_II     ----...... .......... .......... .......... .......... .......... .......... ..........  [ 480]
#L1MdA_III    ----A..... .......... .......... ........A. .......... ...G...... .......... ..........  [ 480]
#L1MdA_IV     ----...... .......... .......... ........A. .......... .......... .......... ..........  [ 480]
#L1MdGf_I     AGGT...... .......... .......... .C......A. .......... .......... .......... T.....G...  [ 480]
#L1MdGf_II    ----...... .T....T... .....C.A.. .C......A. .......... .......... .......... T........A  [ 480]
#L1MdTf_I     ----...... .......... .......... .C......A. .......... .......... ....T..... .........A  [ 480]
#L1MdTf_II    ----...... .......... .......... .C......A. .......... .......... ....T..... .........A  [ 480]
#L1MdTf_III   ----...... .......... .......... .C......A. .........A .......... ....T..... T........A  [ 480]
#L1MdA_V      ----...... .......... .......... .C......A. .......... .......... .......... ..........  [ 480]
#L1MdA_VI     ----...... .T....T... .....C.A.. .C......A. .......... .......... .......... T.........  [ 480]
#L1MdF_V      ----...... .......... .......... .C......A. .......... .......... .......... T.........  [ 480]
#L1MdF_IV     ----...... .......... .......... .C......A. .......... .......... .......... T.........  [ 480]
#L1MdF_I      ----...... .......... .......... .C......A. .......... .......... .......... T.........  [ 480]
#L1MdF_II     ----...... .......... .......... .C......A. .......... .......... ....T..... T........A  [ 480]
#L1MdF_III    ----...... .T....T... .....C.A.. .C......A. .......... .......... .......... T.........  [ 480]
#L1MdN_I      ----...... .......... .......... .C......A. .......... .......... .......... T.........  [ 480]
#L1MdA_VII    ----...... .T....T... .....C.A.. .CA.....A. .......... .......... .......... T.........  [ 480]
#L1MdMus_I    AGGCA..... .T........ .....C.A.. .T......AT .........A .....G.... .......... G...A....T  [ 480]
#L1MdFanc_I   ----...... .T........ .....C.A.. .C......A. .........A .......... .......... T...A.....  [ 480]
#L1MdV_I      ----...... .T........ .....C.AG. .T......AT .........A .......... .......... G...A....T  [ 480]
#L1MdFanc_II  ----...... .T........ .....C.A.. .T......AT .........A .......... ....H..... G...A....T  [ 480]
#L1MdMus_II   ----...... .T........ .....C.A.. .T......AT D........A .....R.... .......... G...A....T  [ 480]
#L1MdV_II     ----..AA.. .T........ .....CYAG. .T......AT .........A .......... ..T.A..... G...A.H..T  [ 480]
#L1Lx_I       ----..A... .T........ .....C.AR. .T......AT .........A .......... ..W.A..... G...A....T  [ 480]
#L1Lx_III     ----..A... .C........ .H.G.C.AG. .T......AT ....A....A .......... ..T.A..... G...A....A  [ 480]
#L1Lx_II      ----..A... .N........ .....C.AGD .T......AT ....V....A .......... ..T.A..... G...A....T  [ 480]
#L1Lx_IV      ----..A... .T........ .....C.AG. .T......NT ....A..D.A .......... ..T.A..... G...A....C  [ 480]
#L1MdV_III    ----..A... .......... .....C.A.. .T......AT ....A....A .......... ..T.A..... G...A....C  [ 480]
```

```
#L1MdA_I       CTGGAGATAG AAACCCTAGG AAAGAGATCT GGAACCATAG ATGCGAGCAT CAGCAACAGA ATACAAGAAA TGGAAGAGAG  [ 560]
#L1MdA_II      .......... .......... .......... .......... .......... .......... .......... ..........  [ 560]
#L1MdA_III     .......... .......... .......... .......... .......... .......... .......... ..........  [ 560]
#L1MdA_IV      .......... .......... .......... .......... .......... .......... .......... ..........  [ 560]
#L1MdGf_I      .......... .......... .....A.... .......... ....C..... .......... ........G. ..........  [ 560]
#L1MdGf_II     G......... ....A..... .....A.... .......... .......... .......... .......... ..........  [ 560]
#L1MdTf_I      ..A....... .......... .....A.... .......... ..TT...... .......... ........G. ..........  [ 560]
#L1MdTf_II     ..A....... .......... .....A.... .......... ..TT...... .......... ........G. ..........  [ 560]
#L1MdTf_III    .......... .......... .....A.... .......... ..TT...... .......... ........G. ..........  [ 560]
#L1MdA_V       .......... .......... .....A.... .......... .C........ .......... ........G. ..........  [ 560]
#L1MdA_VI      .......... ...A...... .....A.... .......... .......... .......... ........G. ..........  [ 560]
#L1MdF_V       .......... .......... .....A.... .......... .......... .......... ........G. ..........  [ 560]
#L1MdF_IV      .......... .......... .....A.... .......... .......... .......... ........G. ..........  [ 560]
#L1MdF_I       .......... .......... .....A.... .......... ....C..... .......... ........G. ..........  [ 560]
#L1MdF_II      .......... .......... .....A.... .......... ..TY...... .......... ........G. ..........  [ 560]
#L1MdF_III     .......... .......... .....A.... .......... .......... .......... ........G. ..........  [ 560]
#L1MdN_I       .......... .......... .....A.... .......... .C........ .......... ........G. ..........  [ 560]
#L1MdA_VII     .......... .......... .....A.... .......... .......... .......... ........G. ..........  [ 560]
#L1MdMus_I     .......... .......... .....A...A .......... ...T...... .......... ........G. ..........  [ 560]
#L1MdFanc_I    .......... .......... .....A.... .......... .......... .......... ........G. ..........  [ 560]
#L1MdV_I       .......... .......... .....A...A .......... .......... .......... ........G. ..........  [ 560]
#L1MdFanc_II   .......... ...T...... .....A...A .......... .......... .......... ........G. ..........  [ 560]
#L1MdMus_II    .......... ...Y...... .....A...A .......... .......... .......... ........G. ..........  [ 560]
#L1MdV_II      .....R.... ...A...... .....AG..A ...R....D. ....A..... A.C....... ........G. .R........  [ 560]
#L1Lx_I        .......... ...T...... .....A...A ...R...... ....A..... ..B....... ........G. .R........  [ 560]
#L1Lx_III      .......... ...A...... .......Y.A ...GT..... ....A..... ..C....... ........G. .A........  [ 560]
#L1Lx_II       .......... ...AN..... .........A ...GT..... .......... ..C....... ........G. .A.......D  [ 560]
#L1Lx_IV       .......... ...A...... .........A ...GT..... ....A..... ..C....... ........G. .A........  [ 560]
#L1MdV_III     ......T... ...A...... .........A ...GT..... ..R.A..... ..C....... ........G. .A........  [ 560]

#L1MdA_I       AATCTCAGGT GCAGAAGATT CCATAGAGAA CATCGACACA ACAGTCAAAG AAAATACAAA ATGCAAAAGG ATCCTAACTC  [ 640]
#L1MdA_II      .......... .......... .......... .......... .......... .......... .......... ..........  [ 640]
#L1MdA_III     .......... .......... .......... .......... .......... .......... .......... ..........  [ 640]
#L1MdA_IV      .......... .......... .......... .....G.... ...A...... .......... .......... ..........  [ 640]
#L1MdGf_I      .......... ........C. .......... .....G.... ...A...... .....GG... ........A. ..........  [ 640]
#L1MdGf_II     .......... .......... .......... .....G.... ...A...... .....GG... ........A. ..........  [ 640]
#L1MdTf_I      .......... .......... .......... .....G.... ...A...... .....GG... ........A. ..........  [ 640]
#L1MdTf_II     .......... .......... .......... .....G.... ...A...... .....GG... ........A. ..........  [ 640]
#L1MdTf_III    .......... .......... .......... .....G.... ...A...... .....GG... ........A. ..........  [ 640]
#L1MdA_V       .......... .......... .......... .....G.... ...A...... .T........ .C......A. ..........  [ 640]
#L1MdA_VI      .......... .......... .......... .....G.... ...A...... .......... ........A. ..........  [ 640]
#L1MdF_V       .......... .......... .......... .....G.... ...A...... .....G.... ........A. ..........  [ 640]
#L1MdF_IV      .......... .......... .......... .....G.... ...A...... .....G.... ........A. ..........  [ 640]
#L1MdF_I       .......... .......... .......... .....G.... ...A...... .....GG... ........A. ..........  [ 640]
#L1MdF_II      .......... .......... .......... .....G.... ...A...... .....GG... ........A. ..........  [ 640]
#L1MdF_III     .......... .......... .......... .....G.... ...A...... .....GV... ........A. ..........  [ 640]
#L1MdN_I       .......... .......... .......... .....G.... ...A...... .T........ .C......A. ..........  [ 640]
#L1MdA_VII     .......... .......... .......... .....G.... ...A...... .......... .C......A. ..........  [ 640]
#L1MdMus_I     .......... .......... ......G... ...G...... ...A...... .....G.... ........A. ..........  [ 640]
#L1MdFanc_I    .......... .......... .......... ...G.G.... ...A...... .....G.... ........A. ..........  [ 640]
#L1MdV_I       .......... .......... ......R... ...G...... ...A...... .....G.... ........A. ..........  [ 640]
#L1MdFanc_II   .......... .......... .......A.. ...G...... ...A...... .....G.... ........A. ..........  [ 640]
#L1MdMus_II    .......... .......... .......R.. ...G...... ...A...... .....G.... ........A. ........Y.  [ 640]
#L1MdV_II      .......... .........A .......A.. .......... ...A...... .....G.G.. ........A. ........Y.  [ 640]
#L1Lx_I        .......... .......... .......A.. .......... ...A...... .....G.... ........A. ........H.  [ 640]
#L1Lx_III      .......... D........A .......A.. ...T...... .B........ .....G.... ..V.....A. N.......C.  [ 640]
#L1Lx_II       .......... .........A ..G....A.. ...T...... ...DB....V .....G.... ........AN N.......C.  [ 640]
#L1Lx_IV       .......... .........A .......A.. ...T...... .......... .....G.... ........A. C.......C.  [ 640]
#L1MdV_III     .........G .........A .......A.. ...T...... .......... .....G.... ........A. C.......C.  [ 640]

#L1MdA_I       AAAACATCCA GGTAATCCAG GACACAATGA GAAGACCAAA CCTACGGATA ATAGGAATTG ATGAGAATGA AGATTTTCAA  [ 720]
#L1MdA_II      .......... .......... .......... .......... .......... .......... .......... ..........  [ 720]
#L1MdA_III     .......... ..A....... .......... .......... .......... .......... .......... ..........  [ 720]
#L1MdA_IV      .......... ..A....... .......... .......... .......... ......G... .......... ..........  [ 720]
#L1MdGf_I      .......... ..A....... .......... .......... .......... ......G.G. .......... ..........  [ 720]
#L1MdGf_II     .......... ..A....... .......... .......G.. .......... ......G.G. .......... ..........  [ 720]
#L1MdTf_I      ....T..... ..A....... ........A. .......... .G........ ......G.G. .......... ..........  [ 720]
#L1MdTf_II     ....T..... ..A....... ........A. .......... .G........ ......G.G. .......... ..........  [ 720]
#L1MdTf_III    ....T..... ..A....... .......... .......... .......... ......G.G. .......... ..........  [ 720]
#L1MdA_V       .......... ..A....... .......... .......... .......... ......G... .......... ..........  [ 720]
#L1MdA_VI      .......... ..A....... .......... .......... .......... ......G.A. .......... ..........  [ 720]
#L1MdF_V       .......... ..A....... .......... .......... .......... ......G.A. .......... ..........  [ 720]
#L1MdF_IV      .......... ..A....... .......... .......... .......... ......G.A. .......... ..........  [ 720]
#L1MdF_I       .......... ..A....... .......... .......... .......... ......G.G. .......... ..........  [ 720]
#L1MdF_II      ....T..... ..A....... .......... .......... .......... ......G.G. .......... ..........  [ 720]
#L1MdF_III     .......... ..A....... .......... .......... .......... ......G.A. .......... ..........  [ 720]
#L1MdN_I       .......... ..A....... .......... .......... .......... ......G... .......... ..........  [ 720]
#L1MdA_VII     .......... ..A....... .......... .......... .......... ......G... .......... ..........  [ 720]
#L1MdMus_I     .......... ..A....... .......... .......... .......... ......G.A. .......... ..........  [ 720]
#L1MdFanc_I    .......... ..A....... .......... .......... .......... ......G.A. .......... ..........  [ 720]
#L1MdV_I       .......... ..A....... .......... .......... .......... ......G.A. .......... ..........  [ 720]
#L1MdFanc_II   .......... ..A....... .......... .......... ....A..... .....T..A. .......... ..........  [ 720]
#L1MdMus_II    .......... ..A....... ..H....... .......... ....A..... ...D.T..A. .......... ..........  [ 720]
#L1MdV_II      ....H..... ..A....... .......... .......... ....A..... .....T..A. .C.....D.. ......Y...  [ 720]
#L1Lx_I        ....N..... ..A....... .......... .......... ....A..... .....T..A. .......... ..........  [ 720]
#L1Lx_III      .......... ..A....... .......... .......... ....A..... .....T..A. .A....G... .....CH...  [ 720]
#L1Lx_II       .....G.... ..A...H... .......... .......... ....A..... .....T..A. .A....GC.. .....HC...  [ 720]
#L1Lx_IV       .......... ..A....... .......... .......... ....A..... .....T..A. .A....G... .....CC...  [ 720]
#L1MdV_III     .......... ..A....... .......... .......... ....A..... .....T..A. .A....G... D....CC...  [ 720]
```

```
#L1MdA_I      CTTAAAGGGC CAGCTAATAT CTTCAACAAA ATAATAGAAG AAAACTTCCC AAACATAAAA AAAGAGATGC CCATGATCAT  [ 800]
#L1MdA_II     .......... .......... .......... .......... .......... .......... .......... ..........  [ 800]
#L1MdA_III    .......... .......... .......... .......... .......... .........G .......... ..........  [ 800]
#L1MdA_IV     .......... ....A..... .......... .......... .......... .........G .......... ..........  [ 800]
#L1MdGf_I     ..C.....A. ....A..C.. .......... ..T..T.... .......... ...TC....G .......... ......A...  [ 800]
#L1MdGf_II    ..C.....A. ....A..C.. .........G ..T..T.... .......... ...TC....G ........A. ......A...  [ 800]
#L1MdTf_I     ..C.....T. ....A..C.. .......... ..T..T.... .......... ...TC....G ..T....... AT....A...  [ 800]
#L1MdTf_II    ..C.....T. ....A..C.. .......... ..T..T.... .......... ...TC....G ..T....... AT....A...  [ 800]
#L1MdTf_III   ..C.....A. ....A..C.. .......... ..T..T.... .......... ...T.....G ........A. .T....A...  [ 800]
#L1MdA_V      .......... .G..A..... .......... ..T....... .......... ....C....G ..T..C.... ......A...  [ 800]
#L1MdA_VI     .......... ....A..... .......... ..T....... .......... ....C....G .......... ......A...  [ 800]
#L1MdF_V      .......... ....A..... .......... ..T....... .......... ....C....G .......... ......A...  [ 800]
#L1MdF_IV     .......... ....A..... .......... ..T....... .......... ....C....G .......... ......A...  [ 800]
#L1MdF_I      ..C.....A. ....A..C.. .......... ..T..T.... .......... ...TC....G .......... ......A...  [ 800]
#L1MdF_II     ..C.....A. ....A..C.. .......... ..T..T.... .......... ...TC....G .......... ......A...  [ 800]
#L1MdF_III    ..C.....A. .G..A..... .......... ..T....... .......... ....C....G .......... ......A...  [ 800]
#L1MdN_I      .......... ....A..... A......... ..T....... .......... ....C....G ..T..C.... ......A...  [ 800]
#L1MdA_VII    .......... ....A..... .......... ..T....... .......... ....C....G ..T..C.... ......A...  [ 800]
#L1MdMus_I    .......... ....A..... .......... ..T....... .......... .T..C....G .......... ......A...  [ 800]
#L1MdFanc_I   .......... ....A..... .......... ..T....... .......... ....C....G .......... ......A...  [ 800]
#L1MdV_I      .......... ....A..... .......... ..T....... .......... .W..C....G .......... ......A...  [ 800]
#L1MdFanc_II  .......... ...TA..... .......... ..T....... .......... T...C....G .......... ......A...  [ 800]
#L1MdMus_II   .......... ...TA..... .......... ..T....... .......... Y...C....G .........Y ......A...  [ 800]
#L1MdV_II     .......... ...TA..... .......... ..T....... .........Y Y...C....G .......... ......A...  [ 800]
#L1Lx_I       .......... ...TA..... .......... ..T....... .......... T...H....G .......... ......A...  [ 800]
#L1Lx_III     .......... ...TA..... .......... ..T....... .......... T...C....G ...N...... ......A...  [ 800]
#L1Lx_II      ......D... ...TA..... .......... ..T....... .......... T...C....G .......... ......A...  [ 800]
#L1Lx_IV      .......... ...TA..... .......... ..T....... .......... T...C....G .......... Y...R.A...  [ 800]
#L1MdV_III    .......... ...TA..... .......... ..T....... .......... T...C....G .......... ......A...  [ 800]

#L1MdA_I      ACAAGAAGCA TACAGAACTC CAAATAGACT GGACCAGAAA AGAAATTCCT CCCGACACAT AATAATCAGA ACAACAAATG  [ 880]
#L1MdA_II     .........C .......... .......... .......... .......... .......... .......... ..........  [ 880]
#L1MdA_III    .........C .......... .......... .......... .......... .......... .......... ..........  [ 880]
#L1MdA_IV     .........C .......... .......... .......... .......... .......... .......... ..........  [ 880]
#L1MdGf_I     .........C .......... .......... .......... .......... .......... .......... ...C......  [ 880]
#L1MdGf_II    .........C .......... .......... .......... .......... .......... .......... ..........  [ 880]
#L1MdTf_I     .........C .......... .......... .......... .......... .......... .......... ...T......  [ 880]
#L1MdTf_II    .........C .......... .......... .......... .......... .......... .......... ...T......  [ 880]
#L1MdTf_III   .........C .......... .......... .......... .......... .......... .......... ..........  [ 880]
#L1MdA_V      .........C .......... .......... .......... .......... .......... .......... ..........  [ 880]
#L1MdA_VI     .........C .......... .......... .......... .......... .......... .......... ..........  [ 880]
#L1MdF_V      .........C .......... .......... .......... .......... .......... .......... ..........  [ 880]
#L1MdF_IV     .........C .......... .......... .......... .......... .......... .......... ..........  [ 880]
#L1MdF_I      .........C .......... .......... .......... .......... .......... .......... ...T......  [ 880]
#L1MdF_II     .........C .......... .......... .......... .......... .......... .......... ..........  [ 880]
#L1MdF_III    .........C .......... .......... .......... .......... .......... .......... ..........  [ 880]
#L1MdN_I      .........C .......... .......... .......... .......... .......... .......... ..........  [ 880]
#L1MdA_VII    .........C .......... .......... .......... .......... ..Y....... .......... ..........  [ 880]
#L1MdMus_I    .........C .......... .......... .......... .......... .......... .......... ..........  [ 880]
#L1MdFanc_I   .........C .......... .......... .......... .......... .......... .......... ..........  [ 880]
#L1MdV_I      .........C .......... .......... .......... .......... .......... .......... ..........  [ 880]
#L1MdFanc_II  .........C .......... .......... .......... .......... .......... .......... ..........  [ 880]
#L1MdMus_II   .........C .......... .......... .......... .......... .......... .......... ..........  [ 880]
#L1MdV_II     .........C .......... .......NT. .......... .......... ....W..... ........A. ...C......  [ 880]
#L1Lx_I       ........YC .......... .......D.. .......... .......... ....H..... ........R. ...C......  [ 880]
#L1Lx_III     .........C .......... ........T. .......... .......... ..T.T..... ........A. ...C....C.  [ 880]
#L1Lx_II      .H.......C ........G. ........N. .......... .......H.. .Y..T.G... ........A. ...C......  [ 880]
#L1Lx_IV      .V.......C .......... ........T. .......... .......... ...AT..... ....G...A. ...C......  [ 880]
#L1MdV_III    .........C .......... .......... .......... .......... ....T..... ........A. ...C......  [ 880]

#L1MdA_I      CACTAAATAA AGATAGAATA TTAAAAGCAG TAAGGGAGAA AGGTCAAGTA ACATATAAAG GAAGGCCTAT CAGAATTACA  [ 960]
#L1MdA_II     .......... .......... .......... .......... .......... .......... .......... ..........  [ 960]
#L1MdA_III    .......... .......... .......... .......... .......... .......... .......... ..........  [ 960]
#L1MdA_IV     .......... .......... .......... .......... .......... .......... .C........ ..........  [ 960]
#L1MdGf_I     .......... .......... C......... .......A.. .......... .......... .C.A...... ..........  [ 960]
#L1MdGf_II    .......... .......... C......... .......A.. .......... .......... .C.A...... ..........  [ 960]
#L1MdTf_I     .......... .......... C......... .......A.. .......... .......... .C.A...... ..........  [ 960]
#L1MdTf_II    .......... .......... C......... .......A.. .......... .......... .C.A...... ..........  [ 960]
#L1MdTf_III   .......... .......... C......... .......A.. .......... .....C.... .C.A...... ..........  [ 960]
#L1MdA_V      .......... ...G...... .......... .......... .......... .......... .C..A..... ..........  [ 960]
#L1MdA_VI     .......... .......... .......... .......A.. .......... .......... .C........ ..........  [ 960]
#L1MdF_V      .......... .......... .......... .......A.. R......... .......... .C........ ..........  [ 960]
#L1MdF_IV     .......... .......... .......... .......A.. .......... .......... .C........ ..........  [ 960]
#L1MdF_I      .......... .......... C......... .......A.. .......... .......... .C.A...... ..........  [ 960]
#L1MdF_II     .......... .......... C......... .......A.. .......... .......... .C.A...... ..........  [ 960]
#L1MdF_III    .......... .......... C......... .......A.. .......... .......... .C.A...... ..........  [ 960]
#L1MdN_I      .......... ...D...... .......... .......... .......... .......... .C..A..... ..........  [ 960]
#L1MdA_VII    .......... ...G...... .......... .......... .......... .......... .C........ ..........  [ 960]
#L1MdMus_I    .......... ...C...... .......... .......A.. .......... .......... .C........ T........T  [ 960]
#L1MdFanc_I   .......... .......... .......... .......A.. .......... .......... .C........ T.........  [ 960]
#L1MdV_I      .......... ...C.D.... .......... .......A.. .......... .......... .C........ T........T  [ 960]
#L1MdFanc_II  .......... .......... .......... .......A.. .......... .......... .C..A..... T.........  [ 960]
#L1MdMus_II   .......... ...CG..... .......... .......A.. .......... .......... .C..R..... T.........  [ 960]
#L1MdV_II     ...A...C.. ...A...... .......... .......A.. D......... .......... .C..A..... ..........  [ 960]
#L1Lx_I       .......Y.. ...H...... .......... .......A.. N......... .......... .C..A..... T.........  [ 960]
#L1Lx_III     .......C.. ...A...... .......... .......A.. .......... .......... .C..A..... ..........  [ 960]
#L1Lx_II      ...A...C.. ...A...... .......... .......A.. .......... .......... .C..A..... ..........  [ 960]
#L1Lx_IV      ...A...C.. ...A...... .......... .......A.. .D........ .......... .C..A..... ..........  [ 960]
#L1MdV_III    ..Y....Y.. ...A...... .......... .......A.. ....B..... .........D .C..AB.... ..........  [ 960]
```

```
#L1MdA_I      CCAGACTTTT CACCAGAGAC TATGAAAGCC AGAAGAGCCT GGACAGATGT TATACAGACA CTAAGAGAAC ACAAATGCCA  [1040]
#L1MdA_II     .......... .......... .......... .......... .......... .......... .......... ..........  [1040]
#L1MdA_III    .......... .......... .......... .......... .......... .......... .......... ..........  [1040]
#L1MdA_IV     .......... .......... .......... .......... .......... .......... .......... ..........  [1040]
#L1MdGf_I     .....T.... .......... .......... .......... ..W....... .......... .......... .Y........  [1040]
#L1MdGf_II    .....T.... .......... .......... .......... .......... .......... .......... ......T...  [1040]
#L1MdTf_I     .....T.... .......... .......... .......... .......... .......... .......... .....CTG..  [1040]
#L1MdTf_II    .....T.... .......... .......... .......... .......... .......... .......... .....CTG..  [1040]
#L1MdTf_III   .....T.... .......... .......... .......... .......... .......... .......... ......T...  [1040]
#L1MdA_V      .......... .......... .......... .......... .......... .......... .......... ..........  [1040]
#L1MdA_VI     .......... .......... .......... ......T... .......... .........M .......... ..........  [1040]
#L1MdF_V      .......... .......... .......... ......T... .......... .......... .......... ..........  [1040]
#L1MdF_IV     .....Y.... .......... .......... ......T... .......... .......... .......... ..........  [1040]
#L1MdF_I      .....T.... .......... .......... .......... .......... .......... .......... ......T...  [1040]
#L1MdF_II     .....T.... .......... .......... .......... .......... .......... .......... ......T...  [1040]
#L1MdF_III    .....T.... .......... .......... .......... .......... .......... .......... ......T...  [1040]
#L1MdN_I      .......... .......... .......... .......... .......... .......... .......... ..........  [1040]
#L1MdA_VII    .......... .......... .......... .......... .......... .......... .......... ..........  [1040]
#L1MdMus_I    ........C. .......... .......... ......T... .......... .......... .......... ..........  [1040]
#L1MdFanc_I   ........C. .......... .......... ......T... .......... ...D.....C .......... ..........  [1040]
#L1MdV_I      ........C. .......... .......... ......T... .......... .......... .......... ..........  [1040]
#L1MdFanc_II  ........C. .......... .......... ......T... .......... .........C .......... ..........  [1040]
#L1MdMus_II   ........C. .......... .......... ......T... .....R.... .........C .......... .Y........  [1040]
#L1MdV_II     ..W.....C. .M........ .......... ......T... ..G....... C........C .......... ..........  [1040]
#L1Lx_I       ........C. .......... .......... ......T... .......... .........C .......... ..........  [1040]
#L1Lx_III     ........C. ..N....... .........T ......T... ..G.....V. C...B....C .......... ..........  [1040]
#L1Lx_II      ........C. .G........ ..C....... ......T... ..G....C.. C........H ......B... ..........  [1040]
#L1Lx_IV      ........C. .......... .........T ......T... ..G.....C. C........C .......... ..........  [1040]
#L1MdV_III    ........C. .......... .........Y ......T... ..G.....C. C........C .......... ..........  [1040]

#L1MdA_I      GCCCAGGCTA CTATACCCGG CCAAACTCTC AATTACCATA GATGGAGAAA CCAAAGTATT CCACGACAAA ACCAAGTTCA  [1120]
#L1MdA_II     .......... .......... .......... .......... .......... .......... .......... .....A....  [1120]
#L1MdA_III    .......... .......... .......... .......... .......... .......... .......... .....A....  [1120]
#L1MdA_IV     .......... .......... .......... .......... .......... .......... .......... .....A....  [1120]
#L1MdGf_I     .......... ........A. .......... .......... .......... .......... .......... ..T..A....  [1120]
#L1MdGf_II    .......... ........A. .......... ...Y...... .......... .......... .......... ..T..A....  [1120]
#L1MdTf_I     .......... ........A. .......... .....T.... ..G....... .......... .......... .....A....  [1120]
#L1MdTf_II    .......... ........A. .......... .....T.... ..G....... .......... .......... .....A....  [1120]
#L1MdTf_III   .......... ........A. .......... .......... .......... .......... .......... .....A....  [1120]
#L1MdA_V      .......... .......... .......... .......... .......... .......... .......... .....A....  [1120]
#L1MdA_VI     .......... ........A. .......... .......... .......... .......... .......... .....A....  [1120]
#L1MdF_V      .......... ........A. .......T.. .......... .......... .......... ...Y...... .....A....  [1120]
#L1MdF_IV     .......... ........A. .......Y.. .......... .......... .......... .......... .....A....  [1120]
#L1MdF_I      .......... ........A. .......... .......... .......... .......... .......... ..T..A....  [1120]
#L1MdF_II     .......... ........A. .......... .......... .......... .......... .......... .....A....  [1120]
#L1MdF_III    .......... ........A. .......... .......... .......... .......... .......... .....A....  [1120]
#L1MdN_I      .......... .......... .......... .......... .......... .......... .......... .....A....  [1120]
#L1MdA_VII    .......... .......... .......... .......... .......... .......... .......... .....A....  [1120]
#L1MdMus_I    .......... ........A. .A........ .......... .......... .......... ...T...... .....A....  [1120]
#L1MdFanc_I   .......... ........A. .A........ .......... .......... .......... ...T..Y... .....A....  [1120]
#L1MdV_I      .......... ........A. .H........ .......... .......... .......... ...T...... .....A....  [1120]
#L1MdFanc_II  .......... ........A. .A........ .......... .......... .......... ...T...... .....A....  [1120]
#L1MdMus_II   .......... ........A. .A........ .......... .......... .......... ...T...... .....A....  [1120]
#L1MdV_II     .......... ........A. .A........ .......... .......... ....G..... ...T...... .....A..T.  [1120]
#L1Lx_I       D.....R... .......HA. .A........ ...H...... .......... .......... ...T...... .....A..Y.  [1120]
#L1Lx_III     D.....A..D ........C. .A........ ........G. .......... ....GA.... ...T...... .....A..T.  [1120]
#L1Lx_II      .......N.. ........A. .A........ .......... .......... ....GA.... ...T...... .MN..A..T.  [1120]
#L1Lx_IV      ......R... ........A. .A........ .......... .......... ....GA.... ...T...... .AH..A..T.  [1120]
#L1MdV_III    .......... ........A. .A........ ......H... .......... .....A.... ...T...... .....A..T.  [1120]

#L1MdA_I      CACAATATCT TTCCACGAAT CCAGCCCTTC AAAGGATAAT AACAGAAAAG AAGCAATACA AGGACGGAAA TCACGCCCTA  [1200]
#L1MdA_II     .......... .......... .......... .......... .......... .......... .......... ..........  [1200]
#L1MdA_III    .......... .......... .......... .......... .......... .......... .......... ..........  [1200]
#L1MdA_IV     ....T..... .......... .......... .......... .........A ..A....... .....A.... ..........  [1200]
#L1MdGf_I     .C..T..... C......... .......... .......... .........A ..M....... .......G.. C.........  [1200]
#L1MdGf_II    .C..T..... C......... .......... .......... .........A ..C....... .......G.. C.........  [1200]
#L1MdTf_I     .G..T..... C......... .......... .......... .........A ..C....... ..A....G.. CA........  [1200]
#L1MdTf_II    .G..T..... C......... .......... .......... .........A ..C....... ..A....G.. CA........  [1200]
#L1MdTf_III   ....T..... C......... .......... .....T.... .........A ..C....... ..A....G.. CA.T......  [1200]
#L1MdA_V      ....T..... .......... .......... .......... .......... .......... .......... ..........  [1200]
#L1MdA_VI     ....T..... .......... .......... .......... .........A ..C....... .......... C.........  [1200]
#L1MdF_V      ....T..... .......... .......... .......... .........A ..AA...... .......... C....Y....  [1200]
#L1MdF_IV     ....T..... .......... .......... .......... .........A ..A....... .......... C....Y....  [1200]
#L1MdF_I      .C..T..... C......... .......... .......... .........A ..C....... .......G.. C.........  [1200]
#L1MdF_II     ....T..... C......... .......... .......... .........A ..C....... ..R....G.. CM........  [1200]
#L1MdF_III    ....T..... .......... .......... .......... .........A ..C....... .......... C....T....  [1200]
#L1MdN_I      ....T..... .......... .......... .......... .......... .......... .......... ..........  [1200]
#L1MdA_VII    ....T..... .......... .......... .......... .........A ..M....... .......... ..........  [1200]
#L1MdMus_I    .......... .......... .......... .......... ..AG.G...A C.C...C... .A..T..... BT..A.....  [1200]
#L1MdFanc_I   .......... .......... .......... .......... ..AG.G...A C.C...C... ....G..... CT..A.....  [1200]
#L1MdV_I      .......... .......... .......... .......... ...G.R...A C.C....... .......... CT........  [1200]
#L1MdFanc_II  .......... .......... .......... .......... ..AG.G...A CTC...C... ....G..... .T.T......  [1200]
#L1MdMus_II   .......... .......... .......... .......... ..AG.G...A CGC...C... .......... .T........  [1200]
#L1MdV_II     .......... ......A... .......... .......... ..AG.G...A CTY...C... .R..G..... CT....Y.G.  [1200]
#L1Lx_I       .......... ......A... .Y........ .......... ..AG.G...A CTC...C... ....G..... CT........  [1200]
#L1Lx_III     ..H.N..... ......A... ........A. .......... .GAT.G...A CGC...C... ....G..... CT..A.....  [1200]
#L1Lx_II      ....N..... ......A... ........W. .......... ..AT.G...A CTC...N... ....N..... CT..A.....  [1200]
#L1Lx_IV      .......... ......A... ........A. .......... .GAT.G...A CGC...Y... ....G..... CT..A.....  [1200]
#L1MdV_III    .......... .....BA... ........A. .......... .GAT.G...A C.C...C... ....G..... CT..A.....  [1200]
```

```
#L1MdA_I      GAACAACCAA GAAAGTAATC ---ATTCAAC AAACCAAAAA GAAGACAGCC ACAAGAACAG AATGCCAACT CTAACAACAA  [1280]
#L1MdA_II     .......... .......... ---....... .......... .......... .......... .......... ..........  [1280]
#L1MdA_III    ......G... .......... ---CC..... .......... .......... .......... .......... ..........  [1280]
#L1MdA_IV     ...A..G... .......... ---CC..... G......... ......W... .......A.. .......... ..........  [1280]
#L1MdGf_I     ...A..A... ...GA..... ---CM..... .....T.... .......A.. .......... .......... T.........  [1280]
#L1MdGf_II    ...A..A... ...GA..... ---CC..... ...A.T.... .......... .......... .......... T.........  [1280]
#L1MdTf_I     ...A..A... ...G...... ---CC..... .....T.... .......... .......... .......C.. T.......T.  [1280]
#L1MdTf_II    ...A..A... ...G...... ---CC..... .....T.... .......... .......... .......C.. T.......T.  [1280]
#L1MdTf_III   ...A..A... ...G...... ---CC..... .....T.... .......... .......... .......... T.........  [1280]
#L1MdA_V      ......G... .......... ---CC..... .......... .......... .......... .......... S.........  [1280]
#L1MdA_VI     ...A..G... .......... ---CC..... .......... .......... .......... .......... ....M.....  [1280]
#L1MdF_V      ...A..G... .......... ---C...... .....T.... .......... .......... .......... ..........  [1280]
#L1MdF_IV     ...A..G... .......... ---C...... .....T.... .......... .......... .......... ..........  [1280]
#L1MdF_I      ...A..A... ...GA..... ---CC..... ...A.T.... .......... .......... .......... T.........  [1280]
#L1MdF_II     ...A..A... ...G...... ---CC..... .....T.... .......... .......... .......... T.........  [1280]
#L1MdF_III    ...A..G... ...G...... ---CC..... .....T.... .......... .......... .......... T.........  [1280]
#L1MdN_I      ......G... .......... ---CC..... .......... .......... .......... .......... ..........  [1280]
#L1MdA_VII    ...A..G... .......... ---CC..... .......... .......... .......... .......... ..........  [1280]
#L1MdMus_I    ...A..G... .......... ---C..T... ...A.T.... .......... .......... .T.C...... T.........  [1280]
#L1MdFanc_I   ...A..G... .......... ---C...... .....T.... .......... G......... ...C...... T.........  [1280]
#L1MdV_I      ...A..G... .......... ---C...... .....T.... .......... .......... ...V...... ..........  [1280]
#L1MdFanc_II  ...A..G... .......... ---C...... .....T.... .....B.... G......... ...C...... ..........  [1280]
#L1MdMus_II   ...A..G... .......... ---C...... .....T.... .......... G......... ...C...... ..........  [1280]
#L1MdV_II     ...A..G... .......... ---T...... .....T.... .....T.... ...T...... ...T...... ...V......  [1280]
#L1Lx_I       ...A..G... .......... ---Y...... ...B.H.... .....N.... ...C...... ...C...... ..........  [1280]
#L1Lx_III     ...A..G... .......... TTCT...... .....CG... .....T.A.. ...CA....T ..AAA----- -.....T...  [1280]
#L1Lx_II      ...A..G... D...R..... TTCT..T... ...N...... .....T..N. ...TA....T ...T.H.CH. .......V..  [1280]
#L1Lx_IV      ...A..G... .......... TTCT...... .....C.... .....T.R.. ...CA....T ..AAV----- -.....T...  [1280]
#L1MdV_III    ...A..G... .......... ---T...... .....C.... .....T.... ...CA....T ...T...C.. ..........  [1280]

#L1MdA_I      AAATAAAAGG GAGCAACAAT TACTTTTCCT TAATATCTCT TAATATCAAT GGACTCAATT CCCCAATAAA AAGACATAGA  [1360]
#L1MdA_II     .......... A......... .......... .......... .......... .......... .......... ..........  [1360]
#L1MdA_III    .......... A......... .......... .......... .......... .......... .......... ..........  [1360]
#L1MdA_IV     .......... A......... .......... .......... .......... .......... .......... ..........  [1360]
#L1MdGf_I     ......C... A......... ...C...... .......... .......... .......... .......... ..........  [1360]
#L1MdGf_II    ...C..C... A......... .......... .......... .......... ..T.....C. .......... ..........  [1360]
#L1MdTf_I     ......C... A......... .......... .......... ...C...... ..T.....C. .G........ ..........  [1360]
#L1MdTf_II    ......C... A......... .......... .......... ...C...... ..T.....C. .G........ ..........  [1360]
#L1MdTf_III   ......C... A......... .......... .......... ...C...... ..T.....C. .......... ..........  [1360]
#L1MdA_V      .......... A......... .......... .......... .......... .......... .......... ..........  [1360]
#L1MdA_VI     ......T... A......... .......... .......... .......... .......... .......... ..........  [1360]
#L1MdF_V      ......T... A......... .......... .......... .......... .......... .......... ..........  [1360]
#L1MdF_IV     ......T... A......... .......... .......... .......... .......... .......... ..........  [1360]
#L1MdF_I      ......C... A......... .......... .......... .......... ..T.....C. .......... ..........  [1360]
#L1MdF_II     ......C... A......... .......... .......... .......... ..T.....C. .......... ..........  [1360]
#L1MdF_III    ......Y... A......... .......... .......... .......... ........C. .......... ..........  [1360]
#L1MdN_I      .......... A......... .......... .......... .......... .......... .......... ..........  [1360]
#L1MdA_VII    .......... A......... .......... .......... .......... .......... .......... ..........  [1360]
#L1MdMus_I    ....G.C... A......... ........T. .G..T..... ...C...... .......... .......... ..........  [1360]
#L1MdFanc_I   ......C... A......... ........T. ....T..... ...C...... .......... .......... ..........  [1360]
#L1MdV_I      ......Y... A......... .......... .......... .......... .......... .......... ..........  [1360]
#L1MdFanc_II  ......C... A......... .......... .......... .......... .......... .......... ..........  [1360]
#L1MdMus_II   ......C... A.....Y... .......... .......... ......H... .......... ...M...... .....G....  [1360]
#L1MdV_II     ......C... A........C G......... .......... .......... .D........ .......... ..........  [1360]
#L1Lx_I       ......C... A......... .......... .......... .......... .......... .......... ....Y.....  [1360]
#L1Lx_III     H.....C... R......... C...A..... .......... ...C...... .......... .......... ..........  [1360]
#L1Lx_II      ......N... A.N....... C......... .......... .......... .......... Y......... ..........  [1360]
#L1Lx_IV      ......C... A......... C...A..... .......... ...C...... .......... .......... ..........  [1360]
#L1MdV_III    ......C... A..T...... C......... .......... ...C...... .......... ...G...... ..........  [1360]

#L1MdA_I      CTAACAGACT GGCTACACAA ACAGGACCCA ACATTCTGCT GCTTACAGGA AACCCATCTC AGGGAAAAAG ACAGACACTA  [1440]
#L1MdA_II     .......... .......... .......... .......... .......... .......... .......... ..........  [1440]
#L1MdA_III    .......... .......... .......... .......... .......... .......... .......... ..........  [1440]
#L1MdA_IV     ........M. .......... .......... .......... .......... .......... .......... ..........  [1440]
#L1MdGf_I     .......... .......... .......... .......... .......... .......... .......... ..........  [1440]
#L1MdGf_II    .......... .......... .......... .......... .......... .......... .......... ..........  [1440]
#L1MdTf_I     ......A... .......... ...A...... .....T.... .......... ...T...... ..A....... .T........  [1440]
#L1MdTf_II    ......A... .......... ...A...... .....T.... .......... ...T...... ..A....... .T........  [1440]
#L1MdTf_III   ......A... .......... ...A...... .....T.... .T........ G..A...... ..A....... .T........  [1440]
#L1MdA_V      .......... .......... .......... .......... .......... .......... .......... ..........  [1440]
#L1MdA_VI     .......... .......... .......... .....Y.... .......... .......... .......... ..........  [1440]
#L1MdF_V      .......... .......... .......... .....T.... .......... .......... .......... ..........  [1440]
#L1MdF_IV     .......... .......... .......... .....T.... .......... .......... .......... ..........  [1440]
#L1MdF_I      ......A... .......... ...A...... .....T.... .......... ...T...... ..A....... .T........  [1440]
#L1MdF_II     ......A... .......... ...A...... .....T.... .......... ...T...... ..A....... .T........  [1440]
#L1MdF_III    .......... .......... .......... .....T.... .......... ...T...... ..A....... .T........  [1440]
#L1MdN_I      .......... .......... .......... .......... .......... .......... .......... ..........  [1440]
#L1MdA_VII    .......... .......... .......... .......... .......... .......... .......... ..........  [1440]
#L1MdMus_I    .......... .......... .......... .....T.... .......... ......C... .....T.... ..........  [1440]
#L1MdFanc_I   ....T..... .......... .......... .....T..B. .......... ......C... ........G. ..........  [1440]
#L1MdV_I      .......... .......... .......... .....T.... .......... .......... .......... ..........  [1440]
#L1MdFanc_II  .......... .......T.. .......... .....T.... ..A....... ......C... .....C.... ..........  [1440]
#L1MdMus_II   .......... .....Y.T.. ......Y... ..T..T.... V......... ..Y...C... .......... .......H..  [1440]
#L1MdV_II     .......... ..T...GT.. .......... ..W..T.... ..A....... ......C... ..K..C.... ..........  [1440]
#L1Lx_I       .......... ..Y....H.. .......... .....T.... ..A....... ......C... .....C.... ..........  [1440]
#L1Lx_III     H....G.... ..A...GT.. .......... D....T.... ..A....... ...G..CH.. ..T.TC.... ...A......  [1440]
#L1Lx_II      .......... R.A...GT.. .N........ .....T.... ..A....... ...G..C... ..T..N.... .......N..  [1440]
#L1Lx_IV      .......... .DA...GT.. .......... G....T.... ..A.....V. ...G..C... ..T.TC.... ...R......  [1440]
#L1MdV_III    .......... ..A..GGT.. .......... G....T.... ..A....... ...G..C... ..T.DC.... ..........  [1440]
```

```
#L1MdA_I      CCTCAGAGTG AAAGGCTGGA AAACAATTTT CCAAGCAAAT GGACTGAAGA AACAAGCTGG AGTAGCCATT TTAATATCGG  [1520]
#L1MdA_II     .......... .......... .......... .......... .......... .......... .......... ..........  [1520]
#L1MdA_III    .......... .......... .......... .......... ..T....... .......... .......... C.........  [1520]
#L1MdA_IV     .......... .......... .......... .......... ..T....... .......... .......... C.G......A  [1520]
#L1MdGf_I     .......... .......... .......... .......... ..T....... ....G..... .......... C.........  [1520]
#L1MdGf_II    .......... .......... .......... .......... ..T....... .......... .......... ..........  [1520]
#L1MdTf_I     .......A.. .......... .......... .......... ..TA...... .......A.. .........C C.......T.  [1520]
#L1MdTf_II    .......A.. .......... .......... .......... ..TA...... .......A.. .........C C.......T.  [1520]
#L1MdTf_III   .......A.A .......... .......... .......... ..TA...... .......... .........C C.......T.  [1520]
#L1MdA_V      .......... .......... .......... .......... ..T....... .......... .......... C.........  [1520]
#L1MdA_VI     .......... .......... .......... .......... ..T....... .......... .......... C........A  [1520]
#L1MdF_V      .......A.. .......... .......... .......... ..T....... .......... .......... C.......KA  [1520]
#L1MdF_IV     .......R.. .......... .......... .......... ..T....... .......... .......... C........A  [1520]
#L1MdF_I      .......A.. .......... .......... .......... ..TA...... .......... .........Y C.......T.  [1520]
#L1MdF_II     .......A.. .......... .......... .......... ..TA...... .......... .........C C.......T.  [1520]
#L1MdF_III    .......A.. .......... .......... .......... ..TA...... .......... .......... C.......TA  [1520]
#L1MdN_I      .......... .......... .......... .......... ..T....... .......... .......... C.........  [1520]
#L1MdA_VII    .......... .......... .......... .......... ..T....... .......... .......... C........A  [1520]
#L1MdMus_I    .........A .......... .......... .......... ..T.C..... .......... .......... C........A  [1520]
#L1MdFanc_I   .......... .......... .......... .......... ..T.C..... .......... .......... C........A  [1520]
#L1MdV_I      .......... .......... .......... .........C ..T....... .......... .......... C........A  [1520]
#L1MdFanc_II  .........A .......... .......... .......... ..T.C..... .......... .......... C........A  [1520]
#L1MdMus_II   ......RD.A .......... .......... .......... ..T.Y..... ......B... .....H.... C........A  [1520]
#L1MdV_II     ...Y.....A .......... ......W... .......... R.T.CC.... ..N....... .......... C........A  [1520]
#L1Lx_I       .......V.D .......... .......... .......... ..T.CC.... .......... .......... C........A  [1520]
#L1Lx_III     .........A ..G....... .......... .......... ..T.CC.... .......G.. .......... C........R  [1520]
#L1Lx_II      N........A .......... ...H....Y. .......... ..T.CN.... .......... .......... C........A  [1520]
#L1Lx_IV      .........A ..G....... .......... .......... ..T.CC.... .......... .......... C........A  [1520]
#L1MdV_III    .........A .....T.... ......H... Y......... ..T.CC.... .......... .......... C.......AA  [1520]

#L1MdA_I      ATAAAATCGA CTTCCAACCC AAAGTTATCA AAAAAGACAA GGAGGGACAC TTCATACTCA TCAAAGGTAA AATCCTCCAA  [1600]
#L1MdA_II     .......... .......... .......... .......... .......... .......... .......... ..........  [1600]
#L1MdA_III    .......... .......... .......... .......... .......... .......... .......... ..........  [1600]
#L1MdA_IV     .......... .......... .......... .......... .......... .......... .......... ..........  [1600]
#L1MdGf_I     .......... .......... .......... .......... .......... .......... .......... ..........  [1600]
#L1MdGf_II    .......... .......... ........T. .......... .......... .......... .......... ..........  [1600]
#L1MdTf_I     ....G..T.. .......... .....C.... .......... .......... .....T.... .......... ..........  [1600]
#L1MdTf_II    ....G..T.. .......... .....C.... .......... .......... .....T.... .......... ..........  [1600]
#L1MdTf_III   ....G..T.. .......... .....C.... .......... ......G... ..YG.T.... .......... ..........  [1600]
#L1MdA_V      .......... .......... .......... .......... .......... .......... .......... ..........  [1600]
#L1MdA_VI     .......... .......... .......... .......... .......... .......... .......... ..........  [1600]
#L1MdF_V      .......... .......... .......... .......... .......... .......... .......... ....Y.....  [1600]
#L1MdF_IV     .......... .......... .......... .......... ......G... .......... .......... ....T.....  [1600]
#L1MdF_I      ....G..T.. .......... .....H.... .......... ......G... .......... .......... ..........  [1600]
#L1MdF_II     ....G..T.. .......... .....C.... .......... ......G... .....T.... .......... ..........  [1600]
#L1MdF_III    .......... .......... .....C.... .......... ......G... .......... .......... ..........  [1600]
#L1MdN_I      .......... .......... .......... .......... .......... .......... .......... ..........  [1600]
#L1MdA_VII    .......... .......... .......... .......... .......... .......... .......... ..........  [1600]
#L1MdMus_I    .......... .......... .......... .......... ......G... .......... .......... ....TA....  [1600]
#L1MdFanc_I   .......T.. .......... .......... .......... ......G... .......... .......... ....TA....  [1600]
#L1MdV_I      .......... .......... .......... .......... ......G... .......... .......... ....T.....  [1600]
#L1MdFanc_II  .......... .......... .......... .......... ......G... .......... .......... ....T.....  [1600]
#L1MdMus_II   .......... .......... .......... .......M.. C.....G... .......... .......... ....T.....  [1600]
#L1MdV_II     .......... ...T.....T ......M... .......Y.. .......... ...W...... .......... ...BTA....  [1600]
#L1Lx_I       .......... ...T.....Y .......... .......... ......G... .......... .......... ....TA....  [1600]
#L1Lx_III     .......... ...T.....A .....C.... .....R.T.. ...AR..... ......Y... .......A.. ....TA....  [1600]
#L1Lx_II      .......... ...T....NT .......... ....D..T.. ...R.N.... .........G ......DA.. ....TA....  [1600]
#L1Lx_IV      .......... ...T.....A .....C.... .......T.. ...A...... .......... .......A.. ....TA....  [1600]
#L1MdV_III    .......... ...T.....A .......... ....G..T.. .R.A...... ........GG .......A.. ....TA....  [1600]

#L1MdA_I      GAGGAACTCT CAATTCTGAA TATCTACGCA CCAAATGCAA GGGCAGCCAC ATTCATTAGA GACACTTTAG TAAAGCTCAA  [1680]
#L1MdA_II     .......... .......... .......... .......... .......... .......... .......... ..........  [1680]
#L1MdA_III    .......... .......... .........T .......... .......... ........A. .......... ..........  [1680]
#L1MdA_IV     .......... .......... ......T..C .......... .......... ........A. .....Y.... ..........  [1680]
#L1MdGf_I     .......... .......... .........T .......... .......... ........A. .......... ..........  [1680]
#L1MdGf_II    .......... .......... .........T .......... .......... ........A. .......... ..........  [1680]
#L1MdTf_I     .......... .......... ......T..T ......A... .A........ .....C..A. ..A....... ..........  [1680]
#L1MdTf_II    .......... .......... ......T..T ......A... .A........ .....C..A. ..A....... ..........  [1680]
#L1MdTf_III   .......... .......... ......T..T ......A... .......... ........A. ..A....... ..........  [1680]
#L1MdA_V      .......... .......... .........T .......... .......... ........A. .......... ..........  [1680]
#L1MdA_VI     .......... .......... ......T..T .......... .......... ........A. ..A....... ..........  [1680]
#L1MdF_V      .......... .......... ......T..T .......... .......... ........A. ..A....... ..........  [1680]
#L1MdF_IV     .......... .......... ......T..T .......... .......... ........A. ..N....... ..........  [1680]
#L1MdF_I      .......... .......... ......T..T ......A... .......... ........A. ..A....... ..........  [1680]
#L1MdF_II     ........M. .......... ......T..T ......A... .......... ........A. ..A....... ....K.....  [1680]
#L1MdF_III    ........A. .......... ......T..T ......A... .......... ........A. ..A....... ..........  [1680]
#L1MdN_I      .......... .......... .........T .......... .......... ........A. .......... ..........  [1680]
#L1MdA_VII    .......... .......... ......T..T .......... .......... ........A. .......... ..........  [1680]
#L1MdMus_I    ..T....... .......A.. ......T..T .......... .......... ........A. ..A....... ..........  [1680]
#L1MdFanc_I   ..T....... .......... ......T..T ......A... .......... ........A. ..A....... ..........  [1680]
#L1MdV_I      ..K....... .......... ......T..T .......... .......... ........A. ..A....... ..........  [1680]
#L1MdFanc_II  ..C....... .......... ......T..T .......... .....T.... ........AG ..A...H... .....Y....  [1680]
#L1MdMus_II   ..T....... .......... ......T..T .......... .....K.... ........AR ..A...H... ..........  [1680]
#L1MdV_II     ..T....... .......... C.....T..T .......... .....T.... ........A. ..A.....RB ....N.....  [1680]
#L1Lx_I       ..T....... .......... C.....T..T .......... .....T.... ........A. ..A....... ..........  [1680]
#L1Lx_III     ..A....... .......... C.....T..T .......... ..VN.C.... ......A.A. ..A......C .....N.A..  [1680]
#L1Lx_II      ..W....... .......... C.....T..T .......... .....C.... ......A.A. ..A......C ..........  [1680]
#L1Lx_IV      ..A....... .......... C.....T..T .......... .....C.... ......A.A. ..A......C ..........  [1680]
#L1MdV_III    ..T....... .......... C.....T..T .......... .....C.... ......A.A. ..A......C ..........  [1680]
```

```
#L1MdA_I      AGCATACATT GCACCTCACA CAATAATAGT GGGAGACTTC AACACACCAC TTTCTTCAAA GGACAGATCG TGGAAACAGA  [1760]
#L1MdA_II     .......... .......... .......... .......... .......... .........D .......... ..........  [1760]
#L1MdA_III    ....C..... .......... .......... .......... .......... ....A....T .......... ..........  [1760]
#L1MdA_IV     ....C..... .......... .......... .......... .......... ....A....T R......... ..........  [1760]
#L1MdGf_I     ....C..... .......... .......... .......C.. .......... ....A....T .......... ..........  [1760]
#L1MdGf_II    ....C..G.. .......... .......... .......... .......... ....A....T .......... ..........  [1760]
#L1MdTf_I     ....C..... ..G....... .......... .......... .......... ....AC...T .........A ..........  [1760]
#L1MdTf_II    ....C..... ..G....... .......... .......... .......... ....AC...T .........A ..........  [1760]
#L1MdTf_III   ....C..... .......... .......... .......... .......... ....AC...T .........A ..........  [1760]
#L1MdA_V      ....C..... .......... .......... .......... .......... ....A....T .......... ..........  [1760]
#L1MdA_VI     ....C.Y... .......... .......... .......... .......... ....A....T .......... ..........  [1760]
#L1MdF_V      ....C..... .......... .......... .......... .......... ....A....T .......... ..........  [1760]
#L1MdF_IV     ....C..... .......... .......... .......... .......... ....A....T .......... ..........  [1760]
#L1MdF_I      ....C..... .......... .......... .......... .......... ....AC...T .........A ..........  [1760]
#L1MdF_II     ....C..... .......... .......... .......... .......... ....AC...T .........A ..........  [1760]
#L1MdF_III    ....C..... .......... .......... .......... .......... ....AC...T .........A ..........  [1760]
#L1MdN_I      ....C..... .......... .......... .......... .......... ....A....T .......... ..........  [1760]
#L1MdA_VII    ....C..... .......... .......... .......... .......... ....A....T .......... ..........  [1760]
#L1MdMus_I    ....C..... .......... .......... .......... ....C..... .C..AC...T .........C ..........  [1760]
#L1MdFanc_I   ....C..... .......... .......... .......... ....C..... .C..AC...T .........C ..........  [1760]
#L1MdV_I      ....C..... .......... .......... .......... ....C..... .Y..A....T .......... ..........  [1760]
#L1MdFanc_II  ....C..... .......... .......... .......... ....C..... .C..A....T .........C ..........  [1760]
#L1MdMus_II   ....C.Y... .Y........ .......... .........H ....C..... .C..A....T .........C ..........  [1760]
#L1MdV_II     ....C..... R......... .......... .......... ....C..... .C..A....T .........H ..........  [1760]
#L1Lx_I       .N..C..... .B...G.... .......... .......... ...NC..... .C..A....T .........H ..........  [1760]
#L1Lx_III     ....C..... .......... .......T.. ...G...... ...NC..... .C..A....T .......N.A G.......C.  [1760]
#L1Lx_II      ....C...N. D......... .......... .N..V.N... ..H.NC.H.. .C..AN...T .....D...A ..........  [1760]
#L1Lx_IV      ....C..... .......... .......... .......... ....C..... .C..A....T .........A ........C.  [1760]
#L1MdV_III    ....C..... R..Y.C.... .......... .........T ....C..... .C..AG...T .....R...A ..........  [1760]

#L1MdA_I      AACTAAACAG GGACACAGTG AAACTAACAG AAGTTATGAA ACAAATGGAC CTGACAGATA TCTACAGAAC ATTTTATCCT  [1840]
#L1MdA_II     .......... .......... .......... .......... .......... .......... .......... ..........  [1840]
#L1MdA_III    .......... .......... .......... .......... .......... .......... .......... ..........  [1840]
#L1MdA_IV     .......... .......... .......... .......... .......... T.A....... .......... ..........  [1840]
#L1MdGf_I     .......... .......... .......... .......... .......... .......... .........T ....A.....  [1840]
#L1MdGf_II    .......... .......... .......... .......... .......... .......... .......... ..........  [1840]
#L1MdTf_I     .......... .......C.. .......... ....G..... .........T .......... .......... .....WC...  [1840]
#L1MdTf_II    .......... .......C.. .......... ....G..... .........T .......... .......... ..........  [1840]
#L1MdTf_III   .......... .......C.. .......... ....G..... .........T .......... .......... ..........  [1840]
#L1MdA_V      .......... .......... .......... .......... .........T .......... .......... ..........  [1840]
#L1MdA_VI     .......... .......... .......... ....K..... .......... T.A....... .......... ..........  [1840]
#L1MdF_V      .......... .......... .......... .......... .........T T.A....... .......... ..........  [1840]
#L1MdF_IV     .......... .......... .......... .......... .........T T.A....... .......... ..........  [1840]
#L1MdF_I      .......... .......... .......... ....G..... .......... T.A....... .......... ..........  [1840]
#L1MdF_II     .......... .......... .......... ....K..... .........T .......... .......... ..........  [1840]
#L1MdF_III    .......... .......... .......... .......... .........T .......... .......... ..........  [1840]
#L1MdN_I      .......... .......... .......... .......... .........T .......... .......... ..........  [1840]
#L1MdA_VII    .......... .......... .......... .......... .......... T.A....... .......... ..........  [1840]
#L1MdMus_I    .......... A......TG. .C........ .......... .........T T.A....... .......... ..........  [1840]
#L1MdFanc_I   .......... A......TG. .C........ .......... .........T T.A.T..... .......... ..........  [1840]
#L1MdV_I      .......... D......... .......... .......... .........T T.A....... .......... ..........  [1840]
#L1MdFanc_II  .......... A......T.. .......... .......... .........T T.A....... .......... ..........  [1840]
#L1MdMus_II   .......... A......B.. .......... .......... .V.......T T.A....... .........H ..........  [1840]
#L1MdV_II     .......... A......... .......... .......... .........T T.A....... ....T..... ....Y.....  [1840]
#L1Lx_I       .......... A......... .......... .......... .........T T.A....... ....T..... ..........  [1840]
#L1Lx_III     .......... A......... .......... .....W..G. C........T T.A.H.B... ...VT..... ....C.....  [1840]
#L1Lx_II      .......... A......... .......... .......... C........T T.A....... ....B....H ..........  [1840]
#L1Lx_IV      .......... A......... .......... ........R. C........T T.A....... ....T..... ....C.....  [1840]
#L1MdV_III    .......... A......... .......... .......... C........T ..A...R... .T..T..... ....C.....  [1840]

#L1MdA_I      AAAACAAAAG GATATACCTT CTTCTCAGCA CCTCACGGGA CCTTCTCCAA AATTGACCAT ATAATTGGTC ACAAAACAGG  [1920]
#L1MdA_II     .......... .......... .......... .......... .......... .......... .......... ..........  [1920]
#L1MdA_III    .......... .......... .......... .......... .......... .......... .......... ..........  [1920]
#L1MdA_IV     .......... .......... .......... .......... .......... .......... .......... .Y........  [1920]
#L1MdGf_I     .......... .......... .......... .......... ...G...... .......... .......... ......T.A.  [1920]
#L1MdGf_II    .......... .......... .......... .......... .......... .......... .......... ..........  [1920]
#L1MdTf_I     .......... .......... .......... .....T..T. .......... .........C .....A.... .....T....  [1920]
#L1MdTf_II    .......... .......... .......... .....T..T. .......... .........C .....A.... .....T....  [1920]
#L1MdTf_III   .......... .......... .......... .....T..T. .......... .........C .....A.... ..........  [1920]
#L1MdA_V      .......... .......... .......... .......... .......... .......... .......... ..........  [1920]
#L1MdA_VI     .......... .......... .......... .....T..T. .......... .......... .......... ..........  [1920]
#L1MdF_V      .......... .......... .......... .....T..T. .......... ..Y....... .......... ..........  [1920]
#L1MdF_IV     .......... .......... .......... .....T..T. .......... .......... .......... ..........  [1920]
#L1MdF_I      .......... .......... .......... .....T..T. .......T.. .........C .......... ........A.  [1920]
#L1MdF_II     .......... .......... .......... .....T..T. .......... .........C .....A.... ..........  [1920]
#L1MdF_III    .......... .......... .......... .....T..T. .......... .........C .......... ........A.  [1920]
#L1MdN_I      .......... .......... .......... .......... .......... .......... ........Y. ..........  [1920]
#L1MdA_VII    .......... .......... .......... .......... .......... .......... .......... ..........  [1920]
#L1MdMus_I    .......... .......... .......... .....T..T. ...C...... .......... .......... ..........  [1920]
#L1MdFanc_I   .......... .......... .........G ..A..T..T. ...C...... .......... .......... ..........  [1920]
#L1MdV_I      .......... .......... .......... .....T..T. .......... .......... .......... ..........  [1920]
#L1MdFanc_II  .......... .......... .......... .....T..T. .......... .......... .......... ..........  [1920]
#L1MdMus_II   .......... .......... .......... .....T..Y. .......... ..YC...... .........H ..........  [1920]
#L1MdV_II     .......... .......... .......... .....T..T. .......... ..Y....... .....C.... ..H.......  [1920]
#L1Lx_I       .......... .......... .......... .....T..T. .......Y.. .......... ...HYC.... ......N...  [1920]
#L1Lx_III     ...T...... A......... .......... .....T..T. .......... ..Y.....G. .....C.... .....T...A  [1920]
#L1Lx_II      .......... A......... .......... .....T..T. .......... .......... .....C.... ..........  [1920]
#L1Lx_IV      ...D...... A......... .......... .....T..T. .......... ..Y....... .....C.... .........A  [1920]
#L1MdV_III    ...G...... A......Y.. .......... .....T..T. .......... ..Y....... ........B. ..........  [1920]
```

```
#L1MdA_I      CCTCAATAGA TACAAAAATA TTGAAATTGT CCCATGTATC CTATCAGACC ACCATGGCCT AAGACTGATC TTCAATAACA  [2000]
#L1MdA_II     ......H... .......... .......... .......... .......... .......... .......... ..........  [2000]
#L1MdA_III    ......C... .......... .......... .......... .......... .......... .......... ..........  [2000]
#L1MdA_IV     ......C... .......... .......... ......Y... .......... .......A.. ...G...... ..........  [2000]
#L1MdGf_I     ......C... .......... .......... .......... .......... .......... .......... ..........  [2000]
#L1MdGf_II    ......C... .......... .......... .......... .......... .......... .......... ..........  [2000]
#L1MdTf_I     ......C... .T........ .......... .......... ........T. ......CA.. ...G...... ..........  [2000]
#L1MdTf_II    ......C... .T........ .......... .......... ........T. ......CA.. ...G...... ..........  [2000]
#L1MdTf_III   ......C... .T........ .......... .......... ........T. ......CA.. ...G...... ..........  [2000]
#L1MdA_V      ......C... .......... .......... .......... .......... .......... ...G...... ..........  [2000]
#L1MdA_VI     ......C... .......... .......C.. ......C... .......... .......A.. ...G...... ..........  [2000]
#L1MdF_V      ......C... .......... .......... ......C... ........T. .......A.. ...G...... ..........  [2000]
#L1MdF_IV     ......C... .......... .......... ......C... ........T. .......A.. ...G...... ..........  [2000]
#L1MdF_I      ......C.T. .......... .......... ......C... ........Y. .......A.. ...G...... ..........  [2000]
#L1MdF_II     ......C... .W........ .......... ......C... ........T. ......CA.. ...G...... ..........  [2000]
#L1MdF_III    ......C.T. .......... .......... ......C... ........T. .......A.. ...G...... ..........  [2000]
#L1MdN_I      ......C... .......... .......... .......... .......... .......... ...G...... ..........  [2000]
#L1MdA_VII    ......C... .......... .......... ......C... .......... .......A.. ...G...... ..........  [2000]
#L1MdMus_I    ......C... .......... ........A. ......C... ........T. ....C..A.. ...G...... ..........  [2000]
#L1MdFanc_I   ....M.C... .......... ........A. ......C... .....T..T. ....C..A.. ...G...... ..........  [2000]
#L1MdV_I      ......C... .......... .......... ......C... ........T. .......A.. ...G...... ..........  [2000]
#L1MdFanc_II  ......C... .......... ........A. ......C... ........T. ....C..A.. ...G...... ..........  [2000]
#L1MdMus_II   ......C... .......... .......HA. ......C... ........T. ....C..A.. ...G...... ..........  [2000]
#L1MdV_II     .....VC... .......... .......AA. ......C.Y. ........T. ....C..A.. ...G...... ..........  [2000]
#L1Lx_I       ......C... .......... .......AA. ......C... ........T. ....C..A.. ...G...... ..........  [2000]
#L1Lx_III     ......C... ..T..G..G. .......AA. .....RCC.. ........T. ..T..R.AT. ...G..G... .......G..  [2000]
#L1Lx_II      ......C... .....G..G. .......AA. ......C... ........T. .N..C..A.. ...G..G... ..........  [2000]
#L1Lx_IV      ......C.N. ..T..G..G. .......AA. ......CM.. ........T. ..G....AT. ...G..G... .......G..  [2000]
#L1MdV_III    ......C... .....G..G. .......AA. ......C.H. ........T. .......A.. ...G..G... ..A...TC..  [2000]

#L1MdA_I      ACATAAATAA TGGAAAGCCA ACATTCACGT GGAAACTGAA TAACACTCTT CTCAATGATA CCTTGGTCAA GGAAGGAATA  [2080]
#L1MdA_II     .......... .......... .......... .......... .......... .......... .......... ..........  [2080]
#L1MdA_III    .......... .......... .......... .......... C......... .......... .......... ..........  [2080]
#L1MdA_IV     .......... .......... ..W....... .......... Y......... .......... .......... ...W......  [2080]
#L1MdGf_I     ....T..G.. .......... .......... .......... C......... .......... .......... ..........  [2080]
#L1MdGf_II    .......... .......... ........D. .......... C......... .......... .......... ..........  [2080]
#L1MdTf_I     .A........ CA........ ........A. .......... C......... .......... .......... ..........  [2080]
#L1MdTf_II    .A........ CA........ ........A. .......... C......... .......... .......... ..........  [2080]
#L1MdTf_III   .A.A...... CA........ ...C...... .......... C......... .......... .......... ..........  [2080]
#L1MdA_V      .......... .......... .......... .......... C......... .......... .......... ..........  [2080]
#L1MdA_VI     .......... .......... .......... .....A.... C......... .......... .......... ..........  [2080]
#L1MdF_V      .......... .A........ .......... .......... C......... .......... .......... ..........  [2080]
#L1MdF_IV     .......... .A........ .......... .......... C......... .......... .......... ..........  [2080]
#L1MdF_I      .A........ .A........ .......... .......... C......... .......... .......... ..........  [2080]
#L1MdF_II     .A........ .A........ .......... .......... C......... .......... .......... ..........  [2080]
#L1MdF_III    .A........ .A........ .......... .......... C......... .......... .......... ..........  [2080]
#L1MdN_I      .......... .......... .......... .......... C......... .......... .......... ..........  [2080]
#L1MdA_VII    .......... .......... .......... .......... C......... .......... .......... ..........  [2080]
#L1MdMus_I    G......... .A........ .......... .......... C........A .......... .......... .....A....  [2080]
#L1MdFanc_I   D......... .A........ .......... .......... C........A .........T .......... ...T.A....  [2080]
#L1MdV_I      .......... .AV....... .......... .......... C........C .......... .......... .....A....  [2080]
#L1MdFanc_II  .......... .A........ .......... ....G..... C........A .......... A......... .....A....  [2080]
#L1MdMus_II   ......R... .A........ .......... ....G..... S....N...A .......... M......... .....A....  [2080]
#L1MdV_II     .......... .A....R..C ....N..... ....G..... C....V...A .......... A......... .....A....  [2080]
#L1Lx_I       .......... .A........ ....A..T.. ....G..... C........A .......... A...C..... .....A....  [2080]
#L1Lx_III     ...A...C.. CA.......C .H..A...A. ....G..... C...G....A .......... A......N.. .....A..K.  [2080]
#L1Lx_II      ...W...C.. .A.......H ....A..... ....G.C... C...G....A .......... A....N.H.. .....A....  [2080]
#L1Lx_IV      ...A...C.. CA.......C ....A...A. ....G..... C...G....A .......... A......... .....A....  [2080]
#L1MdV_III    ...A...Y.. C.....D.AC ....AT..A. ....G..... C..TG....A .......... A......... .....A....  [2080]

#L1MdA_I      AAGAAAGAAA TTAAAGACTT TTTAGAGTTT AATGAAAATG AAGCCACAAC GTACCCAAAC CTATGGGACA CAATGAAAGC  [2160]
#L1MdA_II     .......... .......... .......... .......... .......... .......... .......... ..........  [2160]
#L1MdA_III    .......... .......... .......... .......... .......... .......... ..T....... ..........  [2160]
#L1MdA_IV     .......... .......... .......... .......... .......... A......... .......... ..........  [2160]
#L1MdGf_I     .......... .......... .......... .......... .......... .......... ..T....... ..........  [2160]
#L1MdGf_II    .......... .......... .......... .......... .......... .......... .......... ..........  [2160]
#L1MdTf_I     .......... .......... .......... .......... .......... .......... ..T....... ..........  [2160]
#L1MdTf_II    .......... .......... .......... .......... .......... .......... ..T....... ..........  [2160]
#L1MdTf_III   .......... .......... .......... .......... .......... .......... ..T....... ..........  [2160]
#L1MdA_V      .......... .......... .......... .......... .......... A......... .......... ..........  [2160]
#L1MdA_VI     .......... .......... .......... .......... .......... A......... T......... .W........  [2160]
#L1MdF_V      .......... .......... .......... .......... .......... A......... T......... ..........  [2160]
#L1MdF_IV     .......... .......... .......... .......... .......... A......... T......... ..........  [2160]
#L1MdF_I      .......... ....G..... ...G...... .......... .......... A......... T......... ..........  [2160]
#L1MdF_II     .......... ....G..... C........C .......... .......TT. A......... T......... ..........  [2160]
#L1MdF_III    .......... ....G..... ...G...... .......... .......... A......... T......... ..........  [2160]
#L1MdN_I      .......... .......... .......... .......... .......... A......... .......... ..........  [2160]
#L1MdA_VII    .......... .......... .......... .......... .......... A......... .......... ..........  [2160]
#L1MdMus_I    .......... .......... .......... .......... .......... A......... T......... ..........  [2160]
#L1MdFanc_I   .......... .......... .......... .......... .......... A......... T......... .......G..  [2160]
#L1MdV_I      .......... .......... .......... .......... .......... A......... T......... ..........  [2160]
#L1MdFanc_II  ........R. .......... .......... .......... .......... A......... T......... ..........  [2160]
#L1MdMus_II   .....C.... .......... .......... .......... .......... A......... T......... ..........  [2160]
#L1MdV_II     .......... .......... .......... .......... .......... A..N...... T......... ..........  [2160]
#L1Lx_I       .......... .......... .......... .......... .......... A......... T......... ..........  [2160]
#L1Lx_III     .......... .......... ......A... .......... ...RT..... A..Y...... T......... ..........  [2160]
#L1Lx_II      .......... .......... ......V... .......... .....GN... A......... T......... ..........  [2160]
#L1Lx_IV      .......... .....R.... ......A... .......... ...GY..... A......... T......... ..........  [2160]
#L1MdV_III    .......... .......... ......A... .......... ...A....T. A....A.... T......... ..........  [2160]
```

```
#L1MdA_I      ATTTCTAAGA GGGAAACTCA TAGCGCTGAG TGCCTCCAAG AAGAAACGGG AGACAGCACA TACTAGCAGC TTGACAACAC  [2240]
#L1MdA_II     .......... .......... ....T..... .......... .......... ...V...... .......... ..........  [2240]
#L1MdA_III    .......... .......... ....T..... .......... .......... ...G...... .......... ..........  [2240]
#L1MdA_IV     .......... .......... ....T..... .......... .......T.. ...G...... C......... ..........  [2240]
#L1MdGf_I     .......... .......... ....T..... .A........ .......... ...G...... .......... ..........  [2240]
#L1MdGf_II    .......... .......... ....T..... .......... .......... ...G...... .......... ..........  [2240]
#L1MdTf_I     .......... .......... ....TA.... .....T.... ..A....... ...G...... .......... ..........  [2240]
#L1MdTf_II    .......... .......... ....TA.... .....T.... ..A....... ...G...... .......... ..........  [2240]
#L1MdTf_III   .......... .......... ....T..... ........T. .......... ...G...... .......... ..........  [2240]
#L1MdA_V      .......... .......... ....T..... .......... .......... ...G...... .......... ..........  [2240]
#L1MdA_VI     .......... .......... ....T..... .........A .......TA. ...G...... C......... ..........  [2240]
#L1MdF_V      .......... ..A....... ....T..... .........A .......TA. ...G...... C......... ..........  [2240]
#L1MdF_IV     .......... ..A....... ....T..... .........A .......TA. ...G...... C......... ..........  [2240]
#L1MdF_I      ...C...... ..A....... ....T..... .........A .......TA. ...G...... C.T....... ..........  [2240]
#L1MdF_II     .......... ..A....... ....T..... .........A .......TA. ...G....T. C.T....... ..........  [2240]
#L1MdF_III    .......... ..A....... ....T..... .........A .......TA. ...G...... C.T....... ..........  [2240]
#L1MdN_I      .......... .......... ....T..... .......... .......... ...G...... .......... ..........  [2240]
#L1MdA_VII    .......... .......... ....T..... .........A .......TA. ...G...... C......... ..........  [2240]
#L1MdMus_I    .G.C...... ..A....... ....C..... .........A .......TA. ...G....T. C........T C.....G...  [2240]
#L1MdFanc_I   .G.C...... ..A....... ....C..... .........A .......TA. ...G....T. C......... C.....G...  [2240]
#L1MdV_I      ...Y...... ..A....... ....T..... .........A .......TA. ...G....H. C......... ......G...  [2240]
#L1MdFanc_II  .G.C...... ..A....... ....T..... ....G....A .......TA. ...G....T. C......... ......G...  [2240]
#L1MdMus_II   ...C...... ..A....... ....T..... .........A .......TA. ...G.R..T. C......... Y.....G...  [2240]
#L1MdV_II     .G.C...... ..A....... ....T..... .........A .....D.T.. ..NG....T. C......... ......G...  [2240]
#L1Lx_I       .B.C...... ..A....... ....T..... .........A .......T.. ...G.D..T. Y......... ....H.G...  [2240]
#L1Lx_III     .G.G...... ..A....... ....T..... .........A .......T.. ...G....T. C.......NT ..A...G...  [2240]
#L1Lx_II      .G.N...... ..A....... ....T..... .........A .......T.. ...G....T. C......... ......R.G.  [2240]
#L1Lx_IV      .G.G...... ..A....... ....T..... .........A .......T.. ...G....T. C......... ..A...G...  [2240]
#L1MdV_III    .G.GS..... ..A....... ....T..A.. .........A .......T.. .D.G.VYTT. C........H ......G.G.  [2240]

#L1MdA_I      ATCTAAAAGC CCTAGAAAAA AAGGAAGCAA ATTCACCCAA GAGGAGTAGA CGGCAGGAAA TAATCAAACT CAGGGGTGAA  [2320]
#L1MdA_II     .......... .......... .......... .......... .......... .......... .......... ..........  [2320]
#L1MdA_III    .......... T......... .......... .......... .......... .......... .......... ..........  [2320]
#L1MdA_IV     .......... T......... .......... .......... .......... .......... .......... ..........  [2320]
#L1MdGf_I     .......... T......... .......... .......... .......... .......... .......... ..........  [2320]
#L1MdGf_II    .......... T......... .......... .......... .......... .......... .......... ..........  [2320]
#L1MdTf_I     .......... T......... .......... .......... .......... .......... .......... ..........  [2320]
#L1MdTf_II    .......... T......... .......... .......... .......... .......... .......... ..........  [2320]
#L1MdTf_III   .......... T......... .......... .......... .......... .......... .......... ..........  [2320]
#L1MdA_V      .......... T......... .......... .......... .......... .......... .......... ..........  [2320]
#L1MdA_VI     .C........ T......... .......... .......... .......... .......... .......... ......C...  [2320]
#L1MdF_V      .C........ T......C.. .......... .......... .......... .......... .......... ......C...  [2320]
#L1MdF_IV     .C........ T......C.. .......... .......... .......... .......... .......... ......C...  [2320]
#L1MdF_I      .C.......G T......G.. .......... .......... .......... A......... .......... ..........  [2320]
#L1MdF_II     .C........ T......M.. .......... .......... .......... .......... .......... ......C...  [2320]
#L1MdF_III    .C........ T......C.. .......... .......... .......... .......... .......... ......Y...  [2320]
#L1MdN_I      .......... T......... .......... .......... .......... .......... .......... ..........  [2320]
#L1MdA_VII    .......W.. T......... .......M.. .......... .......... .......... .......... ..........  [2320]
#L1MdMus_I    .C...G.... T......CG. .......... .......... .......... .......... .......... T....CD...  [2320]
#L1MdFanc_I   .C...G.... T......CT. .......... .......... .......... .......... .......... .....C....  [2320]
#L1MdV_I      .C........ T......C.. .......... .......... .......... .......... .......... ....NC....  [2320]
#L1MdFanc_II  .C........ T......C.. .......... .......... .......... .......... ....H..... .....C....  [2320]
#L1MdMus_II   .C........ T......C.. ...R...... .......... .......... .......... .......... .....C....  [2320]
#L1MdV_II     .H..V..... T......C.. ......D... .......... .......... .......... .......... .....C....  [2320]
#L1Lx_I       .C........ T......C.. .......... .......... .......... .......... .......... .....C....  [2320]
#L1Lx_III     .C..G..... T......C.. ..A....... ..A....... .......... .......... .......... .....C....  [2320]
#L1Lx_II      .N..D..... T...R..C.. ..A....N.. ..AN...... .....D.... ...H...... .......... ....DC....  [2320]
#L1Lx_IV      .Y..G..... T......C.. ..A....... ..AB...... .......... .......... H......... .....H....  [2320]
#L1MdV_III    .C..G..... T......C.. ..A....... ..A....... .......... .......... .......... .....C....  [2320]

#L1MdA_I      ATCAACCAAG TGGAAACAAG AAGAACTATT CAAAGAATTA ACCAAACGAG GAGTTGGTTC TTTGAGAAAA TCAACAAGAT  [2400]
#L1MdA_II     .......... .......... .......... .......... .......... .......... .......... ..........  [2400]
#L1MdA_III    .......... .......... .......... .......... .......... .......... .......... ..........  [2400]
#L1MdA_IV     .......... .......... .......... .......... .......... .......... .......... ..........  [2400]
#L1MdGf_I     ..T....... .......... .......... .......... .......... .......... .......... ..........  [2400]
#L1MdGf_II    .......... .......... .......... .......... .......... .......... .....R.... ..........  [2400]
#L1MdTf_I     .......... .......... .......... .......... .......... .......... .......... ..........  [2400]
#L1MdTf_II    .......... .......... .......... .......... .......... .......... .......... ..........  [2400]
#L1MdTf_III   .......... .......... .......... .......... .......... .......... .......... ..........  [2400]
#L1MdA_V      .......... .......... .......... .......... .......... .......... .......... ..........  [2400]
#L1MdA_VI     .......... .......... .......... ........C. .......... ...C...... .......... ..........  [2400]
#L1MdF_V      .......... .......... .......... ........C. .......... ...C...... .......... ..........  [2400]
#L1MdF_IV     .......... .......... .......... ........C. .......... ...C...... .......... ..........  [2400]
#L1MdF_I      .......... .......... .......... ........H. .....W.D.. ...Y...... .......... ..........  [2400]
#L1MdF_II     .......... .......... .......... ..G.....C. .....T.... ...C...... .......... ..........  [2400]
#L1MdF_III    .......... .......... .......... ........C. .....T.... ...C...... .......... ..........  [2400]
#L1MdN_I      .......... .......... .......... .......... .......... .......... .......... ..........  [2400]
#L1MdA_VII    .......... .......... .......... .......... .......... .......... .......... ..........  [2400]
#L1MdMus_I    .......... .........A .......... ........C. .......C.. ...C...... .......... ..........  [2400]
#L1MdFanc_I   .......... .........A .......... ........C. .......C.. ...C...... .......... ..........  [2400]
#L1MdV_I      .......... .........A .......... ........C. .......C.. ...C...... .......... ..........  [2400]
#L1MdFanc_II  .......... .........A .........W ........C. .......C.. ...C...... .......... ..........  [2400]
#L1MdMus_II   .......... .........A .........W ........C. .......C.. ...C...... .......... ..........  [2400]
#L1MdV_II     .......... .D.......A .........A ........C. .......C.. ...Y...... .......... ..........  [2400]
#L1Lx_I       .......... .........A .........A ........C. ......YC.. ...C...... .......... ..........  [2400]
#L1Lx_III     .......... .A.......A .........A ........C. ..A....C.. ...C...... .......... ..........  [2400]
#L1Lx_II      .........R .A.......A ..D..N...A N.......C. .HN...HC.. ..NC....N. .....D.... ..........  [2400]
#L1Lx_IV      .......... .A.......A .........A ........C. ..A....C.. ...C...... .......... ..........  [2400]
#L1MdV_III    .........A .A.......A .........A ........C. ..A....C.. ...C...... .......... ..........  [2400]
```

```
#L1MdA_I     AGATAAACCC TTAGCTAGAC TCACTAAAGG GCACAGGGAC AAAATCCTAA TTAACAAAAT CAGAAATGAA AAGGGAGACA  [2480]
#L1MdA_II    .......... .......... .......... .......... .......... .......... .......... ..........  [2480]
#L1MdA_III   .......... .......... .......... .......... .......... .......... .......... ..........  [2480]
#L1MdA_IV    .......... .......... ......G... .......... .......... .......... .......... ..........  [2480]
#L1MdGf_I    .......... .......... ......G... ...A...... .......... .C........ .......... ..........  [2480]
#L1MdGf_II   .......... .......... ......R... .......... .......... .......... .......... ..........  [2480]
#L1MdTf_I    .......... .......... .......... .......... .......... .......... .......... ..........  [2480]
#L1MdTf_II   .......... .......... .......... .......... .......... .......... .......... ..........  [2480]
#L1MdTf_III  .......... .......... ......G... ......A... .......... .......... .....C.... ..........  [2480]
#L1MdA_V     .......... .......... ......G... .......... ..C....... .......... .......... ..........  [2480]
#L1MdA_VI    .......... .....C.... ......G... .......... .GC....... .......... .......... ..........  [2480]
#L1MdF_V     .......... .....C.... ......G... .......... .GC....... .......... .......... ..........  [2480]
#L1MdF_IV    .......... .....C.... ......G... .......... .GC....... .......... .......... ..........  [2480]
#L1MdF_I     .......... .....Y.... ......G... .........M .RM....... .......... .......... ..........  [2480]
#L1MdF_II    ...C...... .....C.... ......G... .........A .GC..T.... .......... .......... ..........  [2480]
#L1MdF_III   .......... .....C.... ......G... .........A .GC..Y.... .......... .......... ..........  [2480]
#L1MdN_I     .......... .......... ......G... .......... ..C....... .......... .......... ..........  [2480]
#L1MdA_VII   .......... .......... ......G... .......... .GC....... .......... .......... ..........  [2480]
#L1MdMus_I   .......... .....C.... .A....G... .......... .GT....... .......... .......... ..A.......  [2480]
#L1MdFanc_I  .......... .....C.... .A....G... .......... .GT....... .......... ..A....... ..A.......  [2480]
#L1MdV_I     .......... .....C.... ......G... .......... .GC....... .......... .......... ..........  [2480]
#L1MdFanc_II .......... .....C.... .A....G... .......... .GT....... .......... .......... ..........  [2480]
#L1MdMus_II  .......... .....C.... .M....G... .......... .GT....... .......... .......... ..........  [2480]
#L1MdV_II    .......... H....C.... .A....G... .......... .GT....... .......... .......... ..........  [2480]
#L1Lx_I      .......... .....C.... .A....G... .......... .GT....... ....Y..... .......... ..........  [2480]
#L1Lx_III    .......... .....C.... .A..C.G... ......AN.. .GT....A.. .......... .......... ........Y.  [2480]
#L1Lx_II     ...B...... .....C...Y .A..C.G... ......A... .GT..N.H.. .Y.......D .......... ..........  [2480]
#L1Lx_IV     .......... .....C.... .A..C.G... ......A... .GT....A.. .......... .......... ......D.T.  [2480]
#L1MdV_III   .......... .....C.... .A..C.G... R.....A... .GT....A.. ....T..... .......... ..........  [2480]

#L1MdA_I     TAACAACAGA TCCTGAAGAA ATCCAAAACA CCATCAGATC CTTCTACAAA AGGCTATACT CAACAAAACT GGAAAACCTG  [2560]
#L1MdA_II    .......... .......... .......... .......... .......... .......... .......... ..........  [2560]
#L1MdA_III   .......... .......... .......... .......... .......... ...Y...... .......... ..........  [2560]
#L1MdA_IV    .......... .......... .......... .......... .......... .......... .......... ..........  [2560]
#L1MdGf_I    .......... .......... .......... .......... .......... ..CT...... .......... ..........  [2560]
#L1MdGf_II   .......... .......... .......... .......... .......... .......... .......... ..........  [2560]
#L1MdTf_I    .......... .......... .......... .......... .......... .......... .......... ..........  [2560]
#L1MdTf_II   .......... .......... .......... .......... .......... .......... .......... ..........  [2560]
#L1MdTf_III  .......... .......... .......... .......... .......... .......... .......... ..........  [2560]
#L1MdA_V     .......... .......... .......... .......... .......... .......... .......... ..........  [2560]
#L1MdA_VI    .......... .......... .......... .......... .......... .......... .......... ..........  [2560]
#L1MdF_V     .......... .......... .......... .......... .......... .......... .......... ..........  [2560]
#L1MdF_IV    .......... .......... .......... .......... .......... .......... .......... ..........  [2560]
#L1MdF_I     .......... .......... .......... .......... .......... .......... .......... ..........  [2560]
#L1MdF_II    .......... ...Y...... .......... .......... .......... .......... .......... ...G......  [2560]
#L1MdF_III   .......... .......... .......... .......... .......... .......... .......... ...G......  [2560]
#L1MdN_I     .......... .......... .......... .......... .......... .......... .......... ..........  [2560]
#L1MdA_VII   .......... .......... .......... .......... .......... .......... .......... ..........  [2560]
#L1MdMus_I   .......... ......G... .......... T......... ..A....... .......... .......... ..........  [2560]
#L1MdFanc_I  .......... A.....G... .......... T......... ..A....... .......... .......... A.........  [2560]
#L1MdV_I     .......... ......D... .......... .......... .......... .......... .......... ..........  [2560]
#L1MdFanc_II .......... A.....G... .......... T......... ..A....... .......... .......... ..........  [2560]
#L1MdMus_II  ...S...... A.....G... .......... T......... ..A....... .......... .......... ..........  [2560]
#L1MdV_II    .......... AH....G... ........A. T......... ..A....... ..N....... .......... ..........  [2560]
#L1Lx_I      .......... AT....G... ........V. T......... ..A....... ..S....... .......... ..........  [2560]
#L1Lx_III    .......... AA....G... ..Y.....A. T......... ..A....... ..C....... .....M.... D.....T...  [2560]
#L1Lx_II     N.....N... AH...NG... ..NN.N..AD T......... ..A...B... ..CY...... ........Y. ......Y...  [2560]
#L1Lx_IV     H......... AAY...G... ..TA....AW HA........ ..A....... ..N.....Y. .....M.... ......T...  [2560]
#L1MdV_III   .......... AA.C.TG... ..TH....A. T......... ..AH...... ..TT...... ....T...T. ......T...  [2560]

#L1MdA_I     GACGAAATGG ACAAATTTCT GGACAGATAC CAGGTACCAA AGTTGAATCA GGATCAAGTT GACCATCTAA ACAGTCCCAT  [2640]
#L1MdA_II    .......... .......... .......... .......... .......... .......... .......... ..........  [2640]
#L1MdA_III   .......... .......... .......... .......... ......R... .......... .......... ..........  [2640]
#L1MdA_IV    .......... .......... .......... .......... ....A..... .......... A..G...... ..........  [2640]
#L1MdGf_I    .......... .......... .......... .......... .......... .......... ....T..... ..........  [2640]
#L1MdGf_II   .......... .......... .......... .......... .......... .......... .......... ..........  [2640]
#L1MdTf_I    .......... .......... .......... .......... .......... .......... ....T..... ..........  [2640]
#L1MdTf_II   .......... .......... .......... .......... .......... .......... ....T..... ..........  [2640]
#L1MdTf_III  .......... .......... .......... .......... .......... .......... ....T..... ..........  [2640]
#L1MdA_V     .......... .......... A......... .......... ....A..... .......... A..G...... ..........  [2640]
#L1MdA_VI    .......... .......... A......... .......... ....A..Y.. .......... A..G...... ..........  [2640]
#L1MdF_V     ..T....... .......... A......... .......... ....A..... ......G... A.TG...... .......T..  [2640]
#L1MdF_IV    ..T....... .......... A......... .......... ....A..... ......G... A.TG...... .......T..  [2640]
#L1MdF_I     ..Y....... .......... .......... .......... .......... .......... ..YS...... ..........  [2640]
#L1MdF_II    ..T....... .G..G..... A......... .......... .......... A.....G... A.TG...... ....C..G..  [2640]
#L1MdF_III   ..T....... ....G..... A......... .......... ....A..... A.....G... A.TG...... .......T..  [2640]
#L1MdN_I     ..T....... .......... A......... .......... ....A..... .......... A..G...... ..........  [2640]
#L1MdA_VII   .......... .......... A......... .......... ....A..... .......... A..G...... ..........  [2640]
#L1MdMus_I   ..T....... ....C..C.. A......... .......... ....A..... ......GA.. A..G....T. ..........  [2640]
#L1MdFanc_I  ..T....... ....C..C.. A......... .......... ....A..... ......GA.. A.TG...... ..........  [2640]
#L1MdV_I     ..T....... .......... A......... .......... ....A..... ......GA.. A.TG.C.... .......T..  [2640]
#L1MdFanc_II ..T....... ....T...Y. A......... .......... ....A..... ......GA.. A.TG...... ..........  [2640]
#L1MdMus_II  ..T....... ....H..Y.. A......... .......... ....A..... ......GA.. A.TG.C.... ......N...  [2640]
#L1MdV_II    ..T....... ....T..... A......... .......... ....A..... ......GA.. A..G...... ..........  [2640]
#L1Lx_I      ..T....... ....T..... A......... .......... .......... ......GA.. A..G...... ..........  [2640]
#L1Lx_III    ..T....... ....T..... A......... ..RR...... ....A..... ......GA.A A..G...... ..........  [2640]
#L1Lx_II     R.T......D ....T..... AD...V.... ...R...... ....A..... ......GA.A A..G...... ..........  [2640]
#L1Lx_IV     ..T...D... ....T..... A......... ..AA...... ....A..... ......GA.A A......... ..........  [2640]
#L1MdV_III   ..T....... ....T..... A....C.... G....G.... ....A..A.. ......GA.A A......... ..G......N  [2640]
```

```
#L1MdA_I       ATCACCTAAA GAAATAGAAG CAGTTATTAA TAGTCTCCCA ACCAAAAAAA GCCCAGGACC AGATGGGTTT AGTGCAGAGT  [2720]
#L1MdA_II      .......... .......... .......... .......... .......... .......... .......... ..........  [2720]
#L1MdA_III     .......... .......... .......... .......... G......... .......... ...C...... ..........  [2720]
#L1MdA_IV      ...C...... .......... .......... .......... G......... .......... ...C...... ..........  [2720]
#L1MdGf_I      ...C...... .......... .......... .......... .......... .......... .......... ..........  [2720]
#L1MdGf_II     .......... .......... .......C.. .......... G......... .......... ...C...... ..........  [2720]
#L1MdTf_I      ...C...... .......... .......... .......... G......... .......... ...C...... ..........  [2720]
#L1MdTf_II     ...C...... .......... .......... .......... G......... .......... ...C...... ..........  [2720]
#L1MdTf_III    ...C...... .......... .......A.. .......... G......... .......... ...C...... ..........  [2720]
#L1MdA_V       ...C...... .......... ....C..... .......... G......... .......... .......... ..........  [2720]
#L1MdA_VI      ...C...... .......... ....C..... .......... .......... .......... .......... ..........  [2720]
#L1MdF_V       ...C...... .......... ....C..... .......... .......... .......... .......... ..........  [2720]
#L1MdF_IV      ...C...... .......... ....C..... .......... .......... .......... .......... ..........  [2720]
#L1MdF_I       ...C...... .......... .......... .......... G......... .......... ...Y...... ..........  [2720]
#L1MdF_II      ...C..C... .......... ....C..... .......... .......... .......... .......... ..........  [2720]
#L1MdF_III     ...C...... .......... ....C..... .......... .......... .......... .......... ..........  [2720]
#L1MdN_I       ...C.....G .......... ....C..... .......... .......... .......... .......... ..........  [2720]
#L1MdA_VII     ...C...... .......... ....C..... .......... G......... .......... .......... ..........  [2720]
#L1MdMus_I     T..C...... .......... ....C..... .......... .......... .......... .......... ..........  [2720]
#L1MdFanc_I    T..C...... .......... ....C..C.. .......... .......... .......... .......... ..........  [2720]
#L1MdV_I       ...C...... .......... ....C..... .......... .......... AA........ .......... ..........  [2720]
#L1MdFanc_II   ...C...... .......... ....C..... .......... .......... D......... .......... ..........  [2720]
#L1MdMus_II    DN.CH..... .......... .GD.C..... ...Y...... .........D .......... .......... ..........  [2720]
#L1MdV_II      ...C...... .........A ....C..... .......... .......... .N.......Y .......... ..........  [2720]
#L1Lx_I        ...C...... .........A ....C..... .......... .......... .......... .......... ..........  [2720]
#L1Lx_III      .W.C..Y... .........D ....C..... H......... ..H....... ..N....... .N....D... ........R.  [2720]
#L1Lx_II       .A.C...... .........R B.V.C..... .......... ....H..... N.......H. .......... ........V.  [2720]
#L1Lx_IV       .D.C...... .........R ....C..Y.. A.....BY.. .H........ .......... .......... ........A.  [2720]
#L1MdV_III     .A.C...... .......... ....C..... .......... .......... .......... .......... ....G...A.  [2720]

#L1MdA_I       TCTATCAGAC CTTCAAAGAA GATCTAATTC CAATTCTGCA CAAACTATTT CACAAAATAG AAGTAGAAGG TACTCTACCC  [2800]
#L1MdA_II      .......... .......... .......... ..G....... .......... .......... .......... ..........  [2800]
#L1MdA_III     .......... .......... .......... ..G....... .......... .......... .......... ..........  [2800]
#L1MdA_IV      .......... .......... .......... ..G....... .......... .......... .......... ..........  [2800]
#L1MdGf_I      .......... .......... .......... ..G....... .......... .....G.... .......... ..........  [2800]
#L1MdGf_II     .......... .......... .......... ..G....... .......... .......... .......... ..........  [2800]
#L1MdTf_I      .......... .......... .......C.. ..G....... ......T... .....G.... .......... ..T.......  [2800]
#L1MdTf_II     .......... .......... .......C.. ..G....... ......T... .....G.... .......... ..T.......  [2800]
#L1MdTf_III    .......... .......... .......... ..G....... ......T... .....G.... ........A. ..........  [2800]
#L1MdA_V       ....C..... .......... ........C. ..G....... .........C .......... .......G.. A.........  [2800]
#L1MdA_VI      .......... .......... .......... ..G....... .........C .......... .......... ..........  [2800]
#L1MdF_V       .......... .......... .......... ..G....T.. .........C .......... ...C...... ..........  [2800]
#L1MdF_IV      .......... .......... .......... ..G....T.. .........C .......... ...C...... ..........  [2800]
#L1MdF_I       .......... .......... ..C....... ..G....T.. .........C .......... ..AYR..... ..........  [2800]
#L1MdF_II      .......... .......... ........C. ..G....T.. .........C .......... ..AC...... ..........  [2800]
#L1MdF_III     .......... .......... ........C. ..G....T.. .........C .......... ..AC...... ..........  [2800]
#L1MdN_I       ....C..... .......... ........C. ..G....... .........C .......... .......G.. A.........  [2800]
#L1MdA_VII     .......... .......... .......... ..G....... .........C .......... .......... ..........  [2800]
#L1MdMus_I     .......... .......... ..C....... ...C...C.T .........C .......... ..AC...... ..........  [2800]
#L1MdFanc_I    .......... .......... ..C....... ...C...Y.T .........C .......... ..AC...... ..........  [2800]
#L1MdV_I       .......... .......... ..H....... ..G....T.T .........C .......... ..AC...... ..........  [2800]
#L1MdFanc_II   .......... .......... ..C....... .......C.T .........C .......... ..AC...... ..........  [2800]
#L1MdMus_II    ...V...... .......... N.C....... .......C.T .........C .......... ..AC...... ........H.  [2800]
#L1MdV_II      .......... Y........R ..C....... ....W..C.T .........C .......... ..AC...... ..........  [2800]
#L1Lx_I        .......... .......... ..C.....W. ....W..C.T .........C .......... ..AC...... ..........  [2800]
#L1Lx_III      .......... .......... ..C.....AN ....A..CHT .........C .......... ..AC...... A..H......  [2800]
#L1Lx_II       .....N.... .......... ..C.....A. ....A..CTT YG..HN...C .......... ..AC...... A.YA...B..  [2800]
#L1Lx_IV       .......... .......... ..C.....A. ....A..CTT .........C .......... ..AC...... A..A......  [2800]
#L1MdV_III     .......... ...T...... ..C.....A. ....A..CTT .........C .......... ..AC...... D..A......  [2800]

#L1MdA_I       AACTCATTTT ATGAAGCCAC TATTACTCTG ATACCTAAAC CACAGAAAGA TCCAACAAAG ATAGAGAACT TCAGACCAAT  [2880]
#L1MdA_II      .......... .......... .......... .......... .......... C......... .......... ..........  [2880]
#L1MdA_III     .......... .......... .......... .......... .......... C......... .......... ..........  [2880]
#L1MdA_IV      .......... .......... A......... .......... .......... .......... .......... ..........  [2880]
#L1MdGf_I      .......... .......... .......... .......... .......... .......... .......... ..........  [2880]
#L1MdGf_II     .......... .......... .......... .......... .......... C......... .......... ..........  [2880]
#L1MdTf_I      .......... .......... .......... .......... .......... .......... .......... ..........  [2880]
#L1MdTf_II     .......... .......... .......... .......... .......... .......... .......... ..........  [2880]
#L1MdTf_III    .......... .......... .......... .......... .......... .......... .......... ..........  [2880]
#L1MdA_V       ........C. .......... A......... .......... .......... .......... .......... ..........  [2880]
#L1MdA_VI      ........C. .......... A......... .......... .......... .......... .......... ..........  [2880]
#L1MdF_V       ..T.....C. .......... A......... .......... .......... C......... .......... ..........  [2880]
#L1MdF_IV      ..Y.....C. .......... A......... .......... .......... C......... .......... ..........  [2880]
#L1MdF_I       ........C. .......... A......... .......... ....A..... C......... .......... ..........  [2880]
#L1MdF_II      ........C. .......... A......... .......... ....A..... C..C...... .......... ..........  [2880]
#L1MdF_III     ........C. .......... A......... .......... ....A..... C......... .......... ..........  [2880]
#L1MdN_I       ........C. .......... A......... .......... .......... .......... .......... ..........  [2880]
#L1MdA_VII     ........C. .......... A......... .......... .......... .......... .......... ..........  [2880]
#L1MdMus_I     ..T.....C. .......... A......... .......... ....C..... .......... .A........ ..........  [2880]
#L1MdFanc_I    ..T.....C. .......... A......... .......... ....C..... .......... .A........ ..........  [2880]
#L1MdV_I       ..T.....C. .......... A......... .......... ....T..... C......... .......... ..........  [2880]
#L1MdFanc_II   ..T.....C. .......... A......... .......... ....T..... C......... .A........ ..........  [2880]
#L1MdMus_II    ..T.....C. .......... A......... .......... ....T...D. N......... .W......H. ..........  [2880]
#L1MdV_II      ..T..G..C. .......... A......... .......... ....C..... C......... .A........ ..........  [2880]
#L1Lx_I        ..T..G..C. .......... A......... .......... ....C..... C......... .A........ ..........  [2880]
#L1Lx_III      ..T.....C. .......... A.....G... .....D...B ....C..... C....V.... .A........ ..........  [2880]
#L1Lx_II       ..T..G..C. .......... A......... .......... ....C..... CN........ .A........ ..........  [2880]
#L1Lx_IV       ..T..G..C. .......... AR....G... .....A.... ....C..... C......... .A........ ..........  [2880]
#L1MdV_III     ..T..G..C. .......... A.....G..T .......... ....C..... C.A....... .A........ ..........  [2880]
```

```
#L1MdA_I      TTCTCTTATG AATATCGATG CAAAAATCCT CAATAAAATT CTCGCTAACC GAATCCAAGA ACACATTAAA GCAATCATCC  [2960]
#L1MdA_II     .......... .......... .......... .......... .......... .......... .......... ..........  [2960]
#L1MdA_III    .......... .......... .......... .......... .......... .......... .......... ..........  [2960]
#L1MdA_IV     .......... .......... .......M.. .......... .......... .......... .......... ..........  [2960]
#L1MdGf_I     .......... .......... .......... T......... .......... .......... .......... .A........  [2960]
#L1MdGf_II    .......... .......... .......... .......... .......... .......... .......... ..........  [2960]
#L1MdTf_I     .......... ..C....... .......... T......... .......... .......... .......... ..........  [2960]
#L1MdTf_II    .......... ..C....... .......T.. T......... .......... .......... .......... ..........  [2960]
#L1MdTf_III   .......... .......... .......... .......... .......... .......... .......... ..........  [2960]
#L1MdA_V      ...C...... .......... .......... .......... .......... .......... .......... ..........  [2960]
#L1MdA_VI     ...C...... .......... .......A.. .......... .......W.. .......... .......... A.........  [2960]
#L1MdF_V      ...C...... .......... .......M.. .......... .........Y .......... ......C... A.........  [2960]
#L1MdF_IV     ...C...... .......... .......A.. .......... .......... .......... ......C... A.........  [2960]
#L1MdF_I      ...C...... .......... .......... .......G.. ..T....... .......... ......C... A.........  [2960]
#L1MdF_II     A..C...... .....T.... .......... .......G.. ..T....... .......... ......C... A.........  [2960]
#L1MdF_III    ...C...... .......... .......... .......G.. .......... .......... ......C... A.........  [2960]
#L1MdN_I      ...C...... .......... .......... .......... ..T....... .......... .......... ..........  [2960]
#L1MdA_VII    ...C...... .......... .......A.. .......... .......... .......... .......... ..........  [2960]
#L1MdMus_I    ...C...... .......... .......A.. .........C .....A.... .......... ......C... A.G.......  [2960]
#L1MdFanc_I   ...C...... .......... .......A.. .........C .....A.... .......... ......C... A.G.......  [2960]
#L1MdV_I      ...C...... .......... .......A.. .......... .......... .......... ......C... A.G.......  [2960]
#L1MdFanc_II  ...C...... .....Y.... .......A.. .......... .....A.... .......... ......C... A.G.......  [2960]
#L1MdMus_II   ...C...... .......... .......A.. .......... .......V.. .......... ......C... A.........  [2960]
#L1MdV_II     ...C...... .......... .......A.. .......... .....A.... .......... ......C... A.G.......  [2960]
#L1Lx_I       ...C...... .......... .......A.. .......... .....A.... .......... ......C... A.G.......  [2960]
#L1Lx_III     ...C...... .......... .......A.. .......... ..B..H.... .......... ......C... A.G.....Y.  [2960]
#L1Lx_II      ...C...... .......... ......HA.. .......... ..BA.A.... ....V..... ......H... A.G..Y....  [2960]
#L1Lx_IV      ...C...... .....T.... .......A.. .......... ..TD.A.... .......... ......C... ATG.....T.  [2960]
#L1MdV_III    C..C...... ..C..T.... .......A.. .......... ..T..C.... .......... ......C... ATG.....T.  [2960]

#L1MdA_I      ATCCTGACCA AGTAGGTTTT ATTCCAGGGA TGCAGGGATG GTTTAATATA CGAAAATCCA TCAATGTAAT CCATTATATA  [3040]
#L1MdA_II     .......... .......... .......... .......... .......... .......... .......... ..........  [3040]
#L1MdA_III    .......... .......... ........V. .......... .......... .......... .......... ..........  [3040]
#L1MdA_IV     .......... .......... .......... .......... .......... .......... .......... ..........  [3040]
#L1MdGf_I     .......... .......... .......... .......... .......... .......... .......... ..........  [3040]
#L1MdGf_II    .......... .......... .......... .......... .......... .......... .......... ..........  [3040]
#L1MdTf_I     .......... .......... .......... .......... .......... .......... .......... ..........  [3040]
#L1MdTf_II    .......... .......... .......... .......... .......... .......... .......... ..........  [3040]
#L1MdTf_III   .......... .......... ........R. .......... .......... .......... .......... ...C......  [3040]
#L1MdA_V      .......... .......... .......... .......... .......... .......... .......... ..........  [3040]
#L1MdA_VI     .......... .......... .......... .......... .......... ..G....... ....C..... ..........  [3040]
#L1MdF_V      .......... .........C .......... .......... .......... ..G....... ....C..... ...B......  [3040]
#L1MdF_IV     .......... .........C .......... .......... .......... ..G....... ....C..... ..........  [3040]
#L1MdF_I      .......... .........C ..C....... .......... ...Y...... ..G....... .......... ...G......  [3040]
#L1MdF_II     .......... .........C ..C....... .......... ...C...... ..G....... ....H..... ...G......  [3040]
#L1MdF_III    .......... .........C ..C....... .......... ...C...... ..G....... ....C..... ...G......  [3040]
#L1MdN_I      .......... .......... .......... .......... .......... .......... .......... ..........  [3040]
#L1MdA_VII    .......... .......... .......... .......... .......... ..G....... ....C..... ..........  [3040]
#L1MdMus_I    ...A...... ......C..C ..C....... .......... .......... ..G....... ....C..... ...C......  [3040]
#L1MdFanc_I   ...A...... ......C..C ..C....... .......... .......... T.G....... ....C..... ...C......  [3040]
#L1MdV_I      ...A...... ......Y..C ..B....... .......... .......... ..G....... ....C..... ...C......  [3040]
#L1MdFanc_II  ...A...T.. ......C..C ..C....... .......... .......... ..G....... .......... ...C......  [3040]
#L1MdMus_II   ...A...T.. ......C..C .......... .......... .......... ..G....... ....C..... ...C......  [3040]
#L1MdV_II     ...A...T.. ......C..C ..C....... ....R..... ...C...... ..G....... ....C..... Y..C......  [3040]
#L1Lx_I       ...A...T.. ......C..C ..C....... .......... ...C...... ..G....Y.. ....C..... ...C......  [3040]
#L1Lx_III     .H.A...T.. ......C..C ..C....... .......... ...C...... ..G....... ....C..... ...C..Y...  [3040]
#L1Lx_II      ...A...T.. .....VC..C ..C....... .......... ...C...... ..G.....H. .H........ .B.C..B...  [3040]
#L1Lx_IV      .C.A...T.. ......C..C ..C....... .......... ...C...... ..G....Y.. .......... ...C..C...  [3040]
#L1MdV_III    .C.AC..T.. ......C..C ..C....... .......... ...C...... ..G....... ....C..... .T.C..C...  [3040]

#L1MdA_I      AACAAACTCA AAGACAAAAA CCACATGATC ATCTCGTTAG ATGCAGAAAA AGCATTTGAC AAGATCCAAC ACCCATTCAT  [3120]
#L1MdA_II     .......... .......... .......... .......... .......... .......... .......... ..........  [3120]
#L1MdA_III    .......... .......... .......... .......... .......... .......... .....Y.... ..........  [3120]
#L1MdA_IV     .......... .......... .......... .......... .......... .......... .......... ..........  [3120]
#L1MdGf_I     .......... .......... .......... .......... .......... .......... .......... ..........  [3120]
#L1MdGf_II    .......... .......... .......... .......... .......... .......... .......... ..........  [3120]
#L1MdTf_I     .......... .......... .......... .......... .......... .......... .......... ..........  [3120]
#L1MdTf_II    .......... .......... .......... .......... .......... .......... .......... ..........  [3120]
#L1MdTf_III   .......... .......... .......... ........R. .......... .......... .......... ..........  [3120]
#L1MdA_V      .......... .......... .......... .......... ....G..... ......C... .......... ..........  [3120]
#L1MdA_VI     .......... .......... .......... .......... ....G..G.. .......... ..A....... ..........  [3120]
#L1MdF_V      .......... .......... .......... .......... ....G..G.. .......... ..A....... ..........  [3120]
#L1MdF_IV     .......... .......... .......... .......... ....G..G.. .......... ..A....... ..........  [3120]
#L1MdF_I      .......... .......... .......... .......... ....T..G.. .......... ..A....... ..........  [3120]
#L1MdF_II     .......... .......... .......... .......... ....T..G.. .......... ..A....... ..........  [3120]
#L1MdF_III    .......... .......... .......... .......... ....T..G.. .......... ..A....... ..........  [3120]
#L1MdN_I      .......... .......... .......... .......... ....G..... ......C... .......... ..........  [3120]
#L1MdA_VII    .......... .......... .......... .......... ....G..R.. .......... .......... ..........  [3120]
#L1MdMus_I    .......... .......... T......... .......... ....T..G.. .......... ..A....... ....C.....  [3120]
#L1MdFanc_I   .......... .......... .......... .......... ....T..G.. .......... ..A....... ....C.....  [3120]
#L1MdV_I      .......... .......... .......... .......... ....T..G.. .......... ..A....... ..........  [3120]
#L1MdFanc_II  .......... .......... .......... .......... ....T..G.. .......... ..A....... ..........  [3120]
#L1MdMus_II   .......... .......... .......... .......... ....T..G.. .......... ..A....... ..........  [3120]
#L1MdV_II     .......... .......... H......... .....A.... ....T..G.. .......... ..AR...... ....Y.....  [3120]
#L1Lx_I       .......... ..R.M..... .B........ ..H..A...N ....T..G.. ......C..H ..A....... ....G.....  [3120]
#L1Lx_III     .......... ....A..... H..Y...... ..T..A.... ....T..R.. .D........ ..A..Y.... .Y..C.....  [3120]
#L1Lx_II      ..N....... ...RA....N N....B..H. ..Y.YN.... ...HT..G.. .......N.. ..A..B.... ....N.....  [3120]
#L1Lx_IV      .......... ....A..... .......... ..T..A.... ....T..... .N........ ..A..T.... .TY.C.....  [3120]
#L1MdV_III    .......... ....A..... .......... ..T..A.... ....T..... .......D.. ..A..T..G. .T..C.....  [3120]
```

```
#L1MdA_I       GATAAAAGTT TTGGAAAGAT CAGGAATTCA AGGCCCATAC CTAAACATGA TAAAAGCAAT CTACAGCAAA CCAGTAGCCA  [3200]
#L1MdA_II      .......... .......... .......... .......... .......... .......... .......... ..........  [3200]
#L1MdA_III     .......... .......... .......... .......... .......... .......... .......... ..........  [3200]
#L1MdA_IV      .........C .......... .......... .......... .......... .......... .......... ..........  [3200]
#L1MdGf_I      .......... .......... .......... .......... ........A. .......... .......... ..........  [3200]
#L1MdGf_II     .........G .......... .......... .......... .......... .......... .......... ..........  [3200]
#L1MdTf_I      .......... C......... .......... .....A.... .......... .......... .......... ..........  [3200]
#L1MdTf_II     .......... C......... .......... .....A.... .......... .......... .......... ..........  [3200]
#L1MdTf_III    .......... C......... .......... .......... .......... .......... .......... ..........  [3200]
#L1MdA_V       .........C .......... .......... .......... .......... .......... .......... ..........  [3200]
#L1MdA_VI      .........C .......... .......... .......... .......... .......... .......... ..........  [3200]
#L1MdF_V       .........C .......... .......... .......... .......... .......... .......... ..........  [3200]
#L1MdF_IV      .........C .......... .......... .......... .......... .......... .......... ..........  [3200]
#L1MdF_I       .........C .......... .......... .......... .......... .......... .......... ...A......  [3200]
#L1MdF_II      .........C .......... .......... .......... .......... .......... .......... ..........  [3200]
#L1MdF_III     .........C .......... .......... .......... ....M..... .......... .......... ..........  [3200]
#L1MdN_I       .........C .......... .......... .......... .......... .......... .......... ..........  [3200]
#L1MdA_VII     .........C .......... .......... .......... .......... .......... .......... ..........  [3200]
#L1MdMus_I     .........C A......... .......... .......... ........A. .......... A......... ..........  [3200]
#L1MdFanc_I    .........C .......... .......... .......... ........A. .......... V......... ..........  [3200]
#L1MdV_I       .........C .......... .......... .......... ........A. .......... .......... ..........  [3200]
#L1MdFanc_II   .........C .......... .......... .......... ........A. .......... A......... ..........  [3200]
#L1MdMus_II    .........C .......... .......... .......... ........A. .......... H......... ..........  [3200]
#L1MdV_II      .........C .......... ....V..... .......... V.......A. .......... A......... ..........  [3200]
#L1Lx_I        .........C .......... .......... .......... ........A. .......... A......... ..........  [3200]
#L1Lx_III      .W.......C .......... .......... .......... ........AG .......... A......... ..........  [3200]
#L1Lx_II       .........C .......... .......... .......... ........AG .......... V......... ..........  [3200]
#L1Lx_IV       .T.......C .......... .......... .......... ........AG .......... A......... ..........  [3200]
#L1MdV_III     .T.......C .......... .......... .......... ........AG .......... A......... ..........  [3200]

#L1MdA_I       ACATCAAAGT AAATGGAGAG AAGCTGGAAG CAATCCCACT AAAATCAGGG ACTAGACAAG GCTGCCCACT TTCTCCCTAC  [3280]
#L1MdA_II      .......... .......... .......... .......... .......... .......... .......... ..........  [3280]
#L1MdA_III     .......... .......... .......... .......... .......... .......... .......... ..........  [3280]
#L1MdA_IV      .......... .......... .......... .......... .......... .......... .......... ..........  [3280]
#L1MdGf_I      .......... .......... .......... .......... .......... .......... .......... ..........  [3280]
#L1MdGf_II     .......... .......... .......... .......... .......... .......... .......... ..........  [3280]
#L1MdTf_I      .......... .......... .......... .......... .......... .......... .......... ..........  [3280]
#L1MdTf_II     .......... .......... .......... .......... .......... .......... .......... ..........  [3280]
#L1MdTf_III    .......... .......... .......... .......... .......... .......... .......... ..........  [3280]
#L1MdA_V       .......... ......T... .....T.... .......... .......... .......... .......... ..........  [3280]
#L1MdA_VI      .......... ......T... .......... .......... .......... .......... .......... ..........  [3280]
#L1MdF_V       .......... ......T... .......... ..W....... .......... .......... .......... ..........  [3280]
#L1MdF_IV      .......... ......T... .......... .......... .......... .......... ....Y..... ..........  [3280]
#L1MdF_I       .......... ......T... .......... .......... .......... .......... ....T..... C..G......  [3280]
#L1MdF_II      G......... ......T... .....T.... .......... .......... .......... ....T..... C.........  [3280]
#L1MdF_III     .......... ......T... .......... .......... .......... .......... ....T..... C.........  [3280]
#L1MdN_I       .......... ......T... .......... .......... .......... .......... .......... ..........  [3280]
#L1MdA_VII     .......... ......T... .......... .......... .......... .......... .......... ..........  [3280]
#L1MdMus_I     ........N. .......... .....C.... .......... .......... .......... .......... ..........  [3280]
#L1MdFanc_I    ........C. .......... ..A..C.... .......... .......... .......... .......... ..........  [3280]
#L1MdV_I       .......... .......... .......... .......... .......... .......... .......... ..........  [3280]
#L1MdFanc_II   ........C. .......... ..A..T.... .......... .......... .......... .......... ..........  [3280]
#L1MdMus_II    ........S. .......... ..R..NR... .......... .......... .......... .......... ......H...  [3280]
#L1MdV_II      ........C. ......W... ..A..B.... .......... .......... .......... ......H... H.........  [3280]
#L1Lx_I        ........C. .......... ..A..T.... .......... .......... .......... .......... Y.........  [3280]
#L1Lx_III      ........C. .......... ..A..T.... .......... .......... ......H... .N....W... C........H  [3280]
#L1Lx_II       ........C. .......... ..A..T.... .......... ........V. .B........ ....NNY... C........H  [3280]
#L1Lx_IV       ........C. .......... ..A..T.... .......... .......... .......... .......D.. C........T  [3280]
#L1MdV_III     ........C. V......... ..A..T.... .......... .......... .......... .......... C........T  [3280]

#L1MdA_I       CTTTTCAACA TAGTACTTGA AGTATTAGCC AGAGCAATTC GACAACAAAA GGAGATCAAG GGGATACAAA TTGGAAAAGA  [3360]
#L1MdA_II      .......... .......... .......... .......... .......... .......... .......... ..........  [3360]
#L1MdA_III     .......... .......... .......... .......... .......... .......... .......... ..........  [3360]
#L1MdA_IV      ..Y....... .......... ...V...... .......... .......... .......... .......... ..........  [3360]
#L1MdGf_I      .......... .......... .......... .......... .......... .......... .......... ..........  [3360]
#L1MdGf_II     .......... .......... .......... .......... .......... .......... .......... ..........  [3360]
#L1MdTf_I      .......... .......... .......... .......... .......... .......... .......... ..........  [3360]
#L1MdTf_II     .......... .......... .......... .......... .......... .......... .......... ..........  [3360]
#L1MdTf_III    .......... .......... .......... .......... .......... .......... .......... .......G..  [3360]
#L1MdA_V       ..C....... .......... ...CC..... .......... .......... .......... .......... ..........  [3360]
#L1MdA_VI      ..A....... .T........ ...CC..... .......... .......... .......... .......... ..........  [3360]
#L1MdF_V       ..A....... .W........ ...CC..... .......... .......... .......... .......... .......G..  [3360]
#L1MdF_IV      ..A....... .T........ ...CC..... .......... .......... .......... .......... .......G..  [3360]
#L1MdF_I       ..A....... .T........ ...CC..... .........A .......... .......... .......... .......G..  [3360]
#L1MdF_II      ..A....... .T........ ...CC..... .........A .......... .......... .......... .......G..  [3360]
#L1MdF_III     ..A....... .T........ ...CC..... .........A .......... .......... .......... .......G..  [3360]
#L1MdN_I       ..C....... .......... ...CC..... .......... .......... .......... .......... ..........  [3360]
#L1MdA_VII     ..H....... .......... ...CC..... .......... .......... .......... .......... ..........  [3360]
#L1MdMus_I     ..A.....T. .......... ...CC..... .......... .......... .......... .......... .......G..  [3360]
#L1MdFanc_I    ..A.....T. .......... ...HC..... .......... .......... ...B...... .......... .......G..  [3360]
#L1MdV_I       ..A.....T. .......... ...CC..... .......... .......... ....R..... .......... .......G..  [3360]
#L1MdFanc_II   ..A.....T. .......... ...CC..... .........A .......... .......... .......... .......G..  [3360]
#L1MdMus_II    ..A.....T. ........D. ...CC..... .........A .......... ....G....D .......... .......G..  [3360]
#L1MdV_II      ..A.....T. .......... ...CC..... .........A .......... .........D ..R....... .......G..  [3360]
#L1Lx_I        ..A.....T. .......... ...CC..... .........A .......... .........A .......... .......G..  [3360]
#L1Lx_III      ..A.....T. .......... ...TC....H ........HA .......... ....G....A N......... .......G..  [3360]
#L1Lx_II       ..R.....T. .......C.. .D.CC..... .........A .......... ....G....A DN........ .......G..  [3360]
#L1Lx_IV       ..A.....T. .......... ...TC....T .........A .......... ....G....V ......V... .......G..  [3360]
#L1MdV_III     ..A.....T. .......... ...TC....T ..R......A .......... ....G....A .......... .......G..  [3360]
```

```
#L1MdA_I       GGAAGTCAAA ATATCACTTT TTGCAGATGA TATGATAGTA TATATAAGTG ACCCTAAAAA TTCTACCAGA GAACTCCTAA  [3440]
#L1MdA_II      .......... .......... .......... .......... .......... .......... .......... ..........  [3440]
#L1MdA_III     .......... .......... .......... .......... .......... .......... .......... ..........  [3440]
#L1MdA_IV      .......... .......... .......... .......... .......... .......... ...C...... ..........  [3440]
#L1MdGf_I      .......... .......... .......... .......... .......... .......... ...C...... ..........  [3440]
#L1MdGf_II     .......... .......... .......... .......... .......... .......... .......... ..........  [3440]
#L1MdTf_I      .......... .......... .......... .......... .......... .......... ...C.A.... ..........  [3440]
#L1MdTf_II     .......... .......... .......... .......... .......... .......... ...C.A.... ..........  [3440]
#L1MdTf_III    .......... .......... .......... .......... .......... .......... ...C...... ..........  [3440]
#L1MdA_V       D......... .......... .......... .......... .......... .......... ...C...... ..........  [3440]
#L1MdA_VI      A......... .......... .......... .......... .......... .......... ...C...... ..........  [3440]
#L1MdF_V       A......... .......... .......... .......... .......... .......... ...C...... ..........  [3440]
#L1MdF_IV      A......... .......... .......... .......... .......... .......... ...C...... ..........  [3440]
#L1MdF_I       A......... .......... .......... .......... .......... .......... ...C...... ..........  [3440]
#L1MdF_II      A......... W......... .......... .......... ......C... .......... ...C...... ..........  [3440]
#L1MdF_III     A......... .......... .......... .......... .......... .......... ...C...... ..........  [3440]
#L1MdN_I       D......... .......... .......... .......... .......... .......... ...C...... ..........  [3440]
#L1MdA_VII     T......... .......... .......... .......... .......... .......... ...C...... ..........  [3440]
#L1MdMus_I     A......... ........D. .......... .......... .......... .......... ...C...... ..........  [3440]
#L1MdFanc_I    A......... ........A. .......... .......... .......... ....H..... ...C...... ..........  [3440]
#L1MdV_I       A......... ........A. .......... .......... .......... .......... ...C...... ..........  [3440]
#L1MdFanc_II   A......... ........A. .......... .......... .......... .......... ...C...... ..........  [3440]
#L1MdMus_II    A......... ........A. .......... .......... .......V.. .......... ...C...... ..........  [3440]
#L1MdV_II      A......... ........A. .......... .......... .......... ....Y..... ...C...... ..........  [3440]
#L1Lx_I        A......... ........A. .......... .......... ..B....... ....Y..... ...C...... ..........  [3440]
#L1Lx_III      A...R..... ........A. .......... .......V.. ..HT...R.. .Y..H..... H..C...... .........H  [3440]
#L1Lx_II       A......... ........A. .......... .......R.. ..YD...... ....CM.... ...H...... ..........  [3440]
#L1Lx_IV       A......... ........A. .......... .......... ..CT...... ....C..... C..C...... .........C  [3440]
#L1MdV_III     A......... ........A. .......... ......C... ..CT...... ....C..... ...C...... ..........  [3440]

#L1MdA_I       ACCTGATAAA CAGCTTCGGT GAAGTAGCTG GATATAAAAT AAACTCAAAC AAGTCAATGG CCTTTCTCTA TACAAAGAAT  [3520]
#L1MdA_II      .......... .......... .......... .......... .......... .......... .......... ..........  [3520]
#L1MdA_III     .......... .......... .......... .......... .......... .......... .......... ..........  [3520]
#L1MdA_IV      .......... .......... .......... .......... T......... .......... .......... C.........  [3520]
#L1MdGf_I      .......... ......T... .......... .......... T......... .......... .......... C.........  [3520]
#L1MdGf_II     .......... .......... .......... .......... .......... .......... .......... ..........  [3520]
#L1MdTf_I      .......... .......... .......... .......... T......... .......... .......... C.........  [3520]
#L1MdTf_II     .......... .......... .......... .......... T......... .......... .......... C.........  [3520]
#L1MdTf_III    .......... .......... .......... .......... T......... .......... .......... C.........  [3520]
#L1MdA_V       .......... .......... .......... .......... T......... .......... .......... C.........  [3520]
#L1MdA_VI      .......... .......... .......... .......... T......... .......... .......... C.........  [3520]
#L1MdF_V       .......... .......A.. .......... .......... T......... .......... .......N.. C......G..  [3520]
#L1MdF_IV      .......... .......A.. .......... .......... T......... .......... .......... C......G..  [3520]
#L1MdF_I       G......... .......AR. .......... .......... W......... .......... .......G.. C......V..  [3520]
#L1MdF_II      G......... .......A.. .......... .......... T......... .......... .......... C......G.C  [3520]
#L1MdF_III     G......... .......AA. .......... .......... T......... .......... .......G.. C......G..  [3520]
#L1MdN_I       .......... .......... .......... .......... T......... .......... .......... C.........  [3520]
#L1MdA_VII     .......... .......... .......... .......... T......... .......... .......... C.........  [3520]
#L1MdMus_I     .......... .......A.. .C........ .......... T......... ..A...G... .......... C......G..  [3520]
#L1MdFanc_I    .......... .......A.. .C........ .......... T......... ..A....... .......... C......G..  [3520]
#L1MdV_I       .......... .......A.. .H........ .......... T......... ..R....... .......... C......G..  [3520]
#L1MdFanc_II   .......... .......A.. .H........ .......... T......... ..A...G... .......... C......G..  [3520]
#L1MdMus_II    .......... .......A.. .......... .......... T......... ..A...G... .....Y.... C......G..  [3520]
#L1MdV_II      .......... ..A....A.. .H........ .......... T......... ..A...G... .......... C......G..  [3520]
#L1Lx_I        .......... ..A....A.. .......... .......... T......... ..A...G... .......... C......G..  [3520]
#L1Lx_III      .V........ ..A....A.C A....R.... .......... T......... ..A.H.G.R. ....C..V.. CT.....G..  [3520]
#L1Lx_II       .B........ N.A...NA.Y A....G.... .......... T......... ..A...D... ....C..... CW.....G..  [3520]
#L1Lx_IV       .G........ ..A....A.C A.D..G..C. .......... T......... ..A...G.A. ....C..A.. CT.....G..  [3520]
#L1MdV_III     .G........ ..A....A.C A....G.... .......... T......... ..A...G.A. ....CY.... CT.....G.B  [3520]

#L1MdA_I       AAACAGGCTG AGAAAGAAAT TAGGGAAACA ACACCCTTCT CAATAGTCAC AAATAATATA AAATATCTTG GCGTGACTCT  [3600]
#L1MdA_II      .......... .......... .......... .......... .......... .......... .......... ..........  [3600]
#L1MdA_III     .......... .......... .......... .......... .......... .......... .......... ..........  [3600]
#L1MdA_IV      .......... .......... .......... .......... .......... .......... .......... ..........  [3600]
#L1MdGf_I      .......... .......... .......... .....S.... .......... .......... ........C. ..........  [3600]
#L1MdGf_II     .......... .......... .......... .......... .......... .......... .......... ..........  [3600]
#L1MdTf_I      .......... .......... .......... .......... ......C... .......... ........C. ..........  [3600]
#L1MdTf_II     .......... .......... .......... .......... ......C... .......... ........C. ..........  [3600]
#L1MdTf_III    .......... .......... .......... .......... .......... .......... ........C. ..........  [3600]
#L1MdA_V       .......... .......... .......... .......... .......... .......... .......... ..........  [3600]
#L1MdA_VI      .......... .......... .......... .......... .......... .......... .....C.... ..........  [3600]
#L1MdF_V       .......... .......... .......... .......... .......... .......... .....C.... ..........  [3600]
#L1MdF_IV      .......... ...R...... .......... .......... .......... .......... .....C.... ..D.......  [3600]
#L1MdF_I       .......... .......... .......... .......... .......... .......... .....C.... ..........  [3600]
#L1MdF_II      .......... .......... .......... .......... ......C... .......... .....C..A. ..........  [3600]
#L1MdF_III     .......... .......... .......... .......... .......... .......... .....C.... ..........  [3600]
#L1MdN_I       .......... .......... .......... .......... .......... .......... .......... ..........  [3600]
#L1MdA_VII     .......... .......... .......... .......... .......... .......... .......... ..........  [3600]
#L1MdMus_I     .......A.. .......... .......... .........A .......... .......... .....C.... .T........  [3600]
#L1MdFanc_I    ....G.A... .......... .......... .........A .......... .......... .....C.... .T........  [3600]
#L1MdV_I       .......... .......... .......... .........A .......... .......... .....C.... .T........  [3600]
#L1MdFanc_II   .......... .......... .......... .........A .......... .......... .....C.... .T........  [3600]
#L1MdMus_II    .......... .......... .......... ...Y.....A .......... .......... .....C.... KT........  [3600]
#L1MdV_II      .......... .......... ........H. .........A M......... .......... .....C.... .T....B...  [3600]
#L1Lx_I        .......N.. .......... .......... .........A H......... .......... .....C.... .T........  [3600]
#L1Lx_III      .......... .......... .........G .........A .......... ...N...... ..N..B...N .T........  [3600]
#L1Lx_II       ......D... .R........ .......... .....Y...A .......... .......... .....Y.... .T........  [3600]
#L1Lx_IV       .......... .......... .........G .........A .......... ...C...... .......... .T........  [3600]
#L1MdV_III     .......... .......... .........G .........A .....N.... .........T .C...C.... .T........  [3600]
```

```
#L1MdA_I      AACTAAGGAG GTGAAAGATC TGTATGATAA AAACTTCAAA TCTCTGAAGA AAGAAATTAA AGAAGATCTC AGAAGATGGA  [3680]
#L1MdA_II     .........A .......... .......... .......... .......... .......... R......... ..........  [3680]
#L1MdA_III    .......... .......... .......... .......... .......... .......... .......... ..........  [3680]
#L1MdA_IV     .........A .......... .......... .........G .......... .......... .......... ..........  [3680]
#L1MdGf_I     .........A ..A....... .......... .........G .......... .......... .......... ..........  [3680]
#L1MdGf_II    .........A .......... .A........ .......... .......... .......... .......... ..........  [3680]
#L1MdTf_I     ...G.....A .......... .......... .........G ..C....... .......... .......... ..........  [3680]
#L1MdTf_II    ...G.....A .......... .......... .........G ..C....... .......... .......... ..........  [3680]
#L1MdTf_III   .........A .......... .......... .........G .......... .......... .......... ..........  [3680]
#L1MdA_V      .........A .......... .......... .........G ..C....... .......... .......... ..........  [3680]
#L1MdA_VI     .........A .......... .......... G........G .......... .......... .......... ..........  [3680]
#L1MdF_V      .........A .......... .......... G........G .......... .......... .......... ..........  [3680]
#L1MdF_IV     .........A .......... .......... G........G .......... .......... .......... ..........  [3680]
#L1MdF_I      .........A .......... .......... G........G .....A.... .......... .......... ..........  [3680]
#L1MdF_II     .........A .......... .......C.. G.......WG .......... .......... .......... ..........  [3680]
#L1MdF_III    .........A .......... .......... G........G .......... .......... .......... ..........  [3680]
#L1MdN_I      .........A .......... .......... .........G ..C....... .......... .......... ..........  [3680]
#L1MdA_VII    .........A .......... .......... G........G .......... .......... .......... ..........  [3680]
#L1MdMus_I    .........A .......... .......C.. G........G .......... ........D. .......... ..........  [3680]
#L1MdFanc_I   .........A .......... .......... G........G .......... ........G. .......... ..........  [3680]
#L1MdV_I      .........A .......... .......... G........G .......... .......C.. .......... ..........  [3680]
#L1MdFanc_II  .........A .......... .......... G........G .......... .......CG. .......... ..........  [3680]
#L1MdMus_II   .......R.A .......... .K........ G........G .......... .......CG. .......... ........N.  [3680]
#L1MdV_II     .......S.A .......... .......... G........G .......... .......CG. .......... ..........  [3680]
#L1Lx_I       .......S.A .......... .......... G........G .......... .......CG. .......... ..........  [3680]
#L1Lx_III     ...N..RV.A .......... .V.....C.. G........G .......... .......CG. ......H... .........D.  [3680]
#L1Lx_II      .......C.A ..V...D... .......H.. G..H.....G .......... .......CG. ....D..... ..........  [3680]
#L1Lx_IV      ...C..AC.A .......... .......C.. G........G .......... .......CG. ......C... ..........  [3680]
#L1MdV_III    ...C...C.A .......... .D.....Y.. G........G .......... .......C.. .......... ..........  [3680]

#L1MdA_I      AAGATCTCCC ATGCTCATGG ATTGGCAGGA TCAACATTGT AAAAATGGCT ATCTTGCCAA AAGCAATCTA CAGATTCAAT  [3760]
#L1MdA_II     .......... .......... .......... .......... .......... .......... .......... ..........  [3760]
#L1MdA_III    .......... .......... .......... .......... .......... .......... .......... ..........  [3760]
#L1MdA_IV     .......... .......... .......... .......... .......... .......... .......... ..........  [3760]
#L1MdGf_I     .......... .......... .......... .......... .......... .......... .......... ..........  [3760]
#L1MdGf_II    .......... .......... .......... .......... .......... .......... .......... ..........  [3760]
#L1MdTf_I     .......... .......... .......... C......... .......... .......... .......... ..........  [3760]
#L1MdTf_II    .......... .......... .......... C......... .......... .......... .......... ..........  [3760]
#L1MdTf_III   .......... .......... .......... ....Y..... .......... .......... .......... ..........  [3760]
#L1MdA_V      .......... .......... .......... .......... .......... .......... .......... ..........  [3760]
#L1MdA_VI     .......... .......... .......... .......... .......... .......... .......... ..........  [3760]
#L1MdF_V      .......... .......... .......... ....T..A.. .......... .......... .......... ..........  [3760]
#L1MdF_IV     .......... .......... .......... ....T..A.. .......... .......... .......... ..........  [3760]
#L1MdF_I      .......... .......... .......... .......... .........Y .......... .......... ..........  [3760]
#L1MdF_II     .......... .......... ..C....... ....T..A.. .......... .......... .......... ..........  [3760]
#L1MdF_III    .......... .......... .......... ....Y..W.. .......... .......... .......... ..........  [3760]
#L1MdN_I      .......... .......... .......... .......... .......... .......... .......... ..........  [3760]
#L1MdA_VII    .......... .......... .......... .......... .......... .......... .......... ..........  [3760]
#L1MdMus_I    .......... .......... .......... .T..T..A.. .......... ...C....G. .......... ..........  [3760]
#L1MdFanc_I   .......... .......... .......... .T..T..A.. H......... ...C....G. .......... ..........  [3760]
#L1MdV_I      .......... .......... .......... ....T..A.. .......... ........G. .......... ..........  [3760]
#L1MdFanc_II  .......... .......... .......... .T..T..A.. .......... ........G. .......... Y.........  [3760]
#L1MdMus_II   ..N....... .......... .......... .Y..T..H.. .......... ........G. .......... ..........  [3760]
#L1MdV_II     .......... .......... .......... .T..T..A.. M......... ........G. .......... ..........  [3760]
#L1Lx_I       .......... .......... .......... .T..T..A.. .......... ........G. .......... ..........  [3760]
#L1Lx_III     .......... .......... .H........ .T..T..A.. .......N.C ........G. .......... ..........  [3760]
#L1Lx_II      .......... .......... .....Y.... .T..T..A.. .........C ........G. .......... ..........  [3760]
#L1Lx_IV      .......... ...H...... .......... .T..T..A.. .........C .......TG. .......... ..........  [3760]
#L1MdV_III    .......... .......... .......... .T..T..A.. .........C ........G. .......... ..........  [3760]

#L1MdA_I      GCAATCCCCA TCAAAATTCC AACTCAATTC TTCAACGAAT TGGAAGGAGC AATTTGCAAA TTTGTCTGGA ATAACAAAAA  [3840]
#L1MdA_II     .......... .......... .......... .......... .......... .......... .......... ..........  [3840]
#L1MdA_III    .......... .......... .......... .......... .A........ .......... ..CA...... ..........  [3840]
#L1MdA_IV     .......... .......... .......... .......... .A...R.... ...C...... ..CA...... ..........  [3840]
#L1MdGf_I     .......... .......... .......... .......... .A........ .......... ..CA...... ..........  [3840]
#L1MdGf_II    .......... .......... .......... .......... .A........ .......... ..CA...... ..........  [3840]
#L1MdTf_I     .......... .T........ .......... .......... .A........ .......... ..CA...... ..........  [3840]
#L1MdTf_II    .......... .T........ .......... .......... .A........ .......... ..CA...... ..........  [3840]
#L1MdTf_III   .......... .......... .......... .......... .A........ .......... ..CA...... ..........  [3840]
#L1MdA_V      .......... .......... .......... .......... .A...A.... ...C...... ..CA...... ..........  [3840]
#L1MdA_VI     .......... .......... .......... .......... .A...A.... ...C...... ..CA...... ..........  [3840]
#L1MdF_V      .......... .......... .......... .......... .A...A.G.. ...C...... ..CA...... ..........  [3840]
#L1MdF_IV     .......... .......... .......... .......... .A...A.G.. ...C...... ..CA...... ..........  [3840]
#L1MdF_I      .......... .......... .......... .......... .A...A.G.. ...CG...G. ..CA...... ..........  [3840]
#L1MdF_II     .......... .......... .......... .......... .A...A.G.. ...CG...G. ..CA...... ..........  [3840]
#L1MdF_III    .......... .......... .......... ......N... .A...A.G.. ...CG...R. ..CA...... ..........  [3840]
#L1MdN_I      .......... .......... .......... .......... .A...A.... ...C...... ..CA...... ..........  [3840]
#L1MdA_VII    .......... .......... .......... .......... .A...A.... ...C...... ..CA...... ..........  [3840]
#L1MdMus_I    .......... .......... .......... ....CT..G. .A...A.G.. .......... ..CA...... ..........  [3840]
#L1MdFanc_I   .......... .......... .......... ....CT..GW .A...A.G.. .......... ..CA...... ....T.....  [3840]
#L1MdV_I      .......... .......... .......... ....N..... .A...A.G.. .......... ..CA...... ..........  [3840]
#L1MdFanc_II  .......... .......... .......... ....C...G. .A...A.G.. .......... ..CA...... ..........  [3840]
#L1MdMus_II   .......... .......... .......... ....M...R. .A...A.G.. .......... ..CA...... ..........  [3840]
#L1MdV_II     .......... .......... ....H..... ....CA..G. .A...A.R.H .....B.... ..CA...... ..........  [3840]
#L1Lx_I       .....Y.... .......... .......... ....CA..G. .A...A.G.. .......... ..CA...... ..........  [3840]
#L1Lx_III     .......... .......... .......... ....TA..G. .AD..A.... ....C..... ..CA.H.D.. ..........  [3840]
#L1Lx_II      .......... .......... .......... ....TA..G. .A...A...Y .....B.... ..CA.H.... ..........  [3840]
#L1Lx_IV      .......... .......... ..B....... ....TA..G. .A...A.... ....CT.... ..CA.T.... ..........  [3840]
#L1MdV_III    .......... .......... ..A....... ....TA..G. .A...A.... .......... ..CA.T.... ..........  [3840]
```

```
#L1MdA_I      ACCTAGGATA GCAAAAAGTC TTCTCAAGGA TAAAAGAACT TCTGGCGGAA TCACCATGCC AGACCTAAAG CTTTACTACA  [3920]
#L1MdA_II     .......... .......... .......... .......... .......... .......... .......... ..........  [3920]
#L1MdA_III    .......... .......... .......... .........C .....T.... .......... T......... ..........  [3920]
#L1MdA_IV     .......... .......C.. .......... .........C .....T.... .......... T......... ..........  [3920]
#L1MdGf_I     .......... .......C.. .......... .........C .....T.... .......... T......... ..........  [3920]
#L1MdGf_II    .......... .......... .......... .........C .....T.... .......... T......... ..........  [3920]
#L1MdTf_I     ...G...... .......C.. .......... .........C .....T.... .......... T......... ..........  [3920]
#L1MdTf_II    ...G...... .......C.. .......... .........C .....T.... .......... T......... ..........  [3920]
#L1MdTf_III   .......... .......C.. .......... .........C .....T.... .......... T......... ..........  [3920]
#L1MdA_V      .......... ......GC.. .......... ......T..C .....T.... .......... T......... ..........  [3920]
#L1MdA_VI     .......... .......C.. .......... .........C .....T.... .......... T......... ..........  [3920]
#L1MdF_V      .......... .......C.. .......K.. .........C .....T.... .......... T......... ..G.......  [3920]
#L1MdF_IV     .......... .......C.. .......... .........C .....T.... .......... T......... ..D.......  [3920]
#L1MdF_I      ...G...... .......C.. .......... .........C .....T.... .......... G........A ..G.......  [3920]
#L1MdF_II     .......... .......C.. .......... .........C .....T.... .......... C......... M.G.......  [3920]
#L1MdF_III    .......... .......C.. .......... .........C .....T.... .......... T......... ..G.......  [3920]
#L1MdN_I      .......... ......GC.. .......... ......T..C .....T.... .......... T......... ..........  [3920]
#L1MdA_VII    .......... .......C.. .......... .........C .....T.... .......... T......... ..........  [3920]
#L1MdMus_I    .......... .......C.A .......CA. .........C .....B.... .......... T.....C... ..G.......  [3920]
#L1MdFanc_I   .......... .......C.A .......CA. .........C .....T.... .......... T...H.H... ..G.......  [3920]
#L1MdV_I      .......... .......C.. .......T.. ........YC .....T.... .......... T......... ..G.......  [3920]
#L1MdFanc_II  .......... .......C.A .......CA. .........C .....T.... .......... T.....C... ..G.......  [3920]
#L1MdMus_II   .......... .......C.M .......C.. .........C ....DT.... .......... T.....H... ..G.......  [3920]
#L1MdV_II     N......... .......C.A .......CA. .........C .....T.... .......N.. TD....C... ..G.......  [3920]
#L1Lx_I       .......... .......C.A .......CA. .........C .....T.... .......B.. T.....C... ..G.......  [3920]
#L1Lx_III     .H.Y...... ..G....C.A .......CA. N.......NN .....B.... .......C.. T.....C... ..G.......  [3920]
#L1Lx_II      ...C...... ..G....C.A .......HA. .......NY. .......... .......N.. T.....C... ..G.......  [3920]
#L1Lx_IV      ...C...... ..G....C.A .......CA. ........H. .....G.... ...S...C.. T.....C... ..G.....N.  [3920]
#L1MdV_III    ...C...... ..G....C.A .......CA. .......... .....G.... .......C.. T.....C... ..G..T....  [3920]

#L1MdA_I      GAGCAATTGT GATAAAAACT GCATGGTACT GGTATAGAGA CAGACAAGTA GACCAATGGA ATAGAATTGA AGATCCAGAA  [4000]
#L1MdA_II     .......... .......... .......... .......... .......... .......... .......... ...C......  [4000]
#L1MdA_III    .......... .......... .......... .......... .......... .......... .......... ...C......  [4000]
#L1MdA_IV     .......... .......... .......... ......VT.. .......... .......... .......... ...C......  [4000]
#L1MdGf_I     .G........ .......... .......... .......... .......... .......... .......... ...C......  [4000]
#L1MdGf_II    .......... .......... .......... .......... .....C.... .......... .......... ...C......  [4000]
#L1MdTf_I     .......... .......... .......... .......... .........G .......... .......... ...C......  [4000]
#L1MdTf_II    .......... .......... .......... .......... .........G .......... .......... ...C......  [4000]
#L1MdTf_III   .......... .G........ .......... .......... .......... .......... .......... ...C......  [4000]
#L1MdA_V      .......... T......... .......... .......T.. .......... .......... .......... ...C......  [4000]
#L1MdA_VI     .......... .......M.. .......... .......C.. .......... .......... .......... ...C......  [4000]
#L1MdF_V      .......... .......... .......... .......C.. .......... .......... .......... ...C......  [4000]
#L1MdF_IV     .......... .......... .......... .......C.. .......... .......... .......... ...C......  [4000]
#L1MdF_I      .......... ...C...... .......... .......T.. .......... .......... .C........ ...C.....G  [4000]
#L1MdF_II     .......... .......... .......... .......C.. .......... .......... .C........ ...C.....G  [4000]
#L1MdF_III    .......... .......... .......... .......C.. .......... .......... .C........ ...C.....G  [4000]
#L1MdN_I      .......... .......... .......... .......T.. .......... .......... .......... ...C......  [4000]
#L1MdA_VII    .......... .......... .......... .......T.. .......... .......... .......... ...C......  [4000]
#L1MdMus_I    .......... .......... .......... ....C..C.. ......G... ..T....... .......... ...C......  [4000]
#L1MdFanc_I   .......... .......... .......... ....C..T.. ......G... ..T....... .......... ...C......  [4000]
#L1MdV_I      .......... .......... .......... .......C.. ......V... .......... .......... ...C......  [4000]
#L1MdFanc_II  .......... .......... .......... ....C..C.. ......G... ..T....... ......Y... ...C......  [4000]
#L1MdMus_II   .......... .......... ........Y. ....C..C.. ......G... ..Y....... .......... ...C......  [4000]
#L1MdV_II     .......... .......... .......... ....C..T.. ......G... ..T....... .Y........ ...C......  [4000]
#L1Lx_I       .......... .......... .......... ....C..C.. ...R..G... ..T....... .......... ...C......  [4000]
#L1Lx_III     .......D.. .......... ........T. ....H..T.. ...R..G... ..T....... .......... ...C......  [4000]
#L1Lx_II      ..R....H.. .......... ........Y. ....C..H.. N..DB.G... ..T....... ......B... ...C......  [4000]
#L1Lx_IV      .......D.. .......... ........T. ....C..T.. ...G..R... ..T....... .......... ...C......  [4000]
#L1MdV_III    .......A.. .......... ........Y. ....C..... ...V..G.A. ..T....... .......... ...C......  [4000]

#L1MdA_I      ATGAACCCAC ACACCTATGG TCACTTGATC TTCGACAAGG GAGCTAAAAC CATCCAGTGG AAGAAAGACA GCATTTTCAA  [4080]
#L1MdA_II     .......... .......... .......... .......... .......... .......... .......... ..........  [4080]
#L1MdA_III    .......... .......... .......... .......... .......... .......... .......... ..........  [4080]
#L1MdA_IV     .......... .......... .......... .......... .......... .......... .......... ..........  [4080]
#L1MdGf_I     .......... .......... .......... .......... .......... .......... .......... ..........  [4080]
#L1MdGf_II    .......... .......... .......... .......... .......... .......... .......... ..........  [4080]
#L1MdTf_I     .......... .......... .......... .......... ....C..... .......... .......... ..........  [4080]
#L1MdTf_II    .......... .......... .......... .......... ....C..... .......... .......... ..........  [4080]
#L1MdTf_III   .......... .......... .......... .......... .......... .......... .......... ..........  [4080]
#L1MdA_V      .......... .......... .......... .......... .......... .......... .......... ..........  [4080]
#L1MdA_VI     .......... .......... .......... .......... .......... .......... ..A....... ..........  [4080]
#L1MdF_V      .......... .......... .......... ..T....... .......... .......... ..A....... ..........  [4080]
#L1MdF_IV     .......... .......... .......... ..T....... .......... .......... ..A....... ..........  [4080]
#L1MdF_I      .....Y.... .......... .......... ..T....... .......... .......... ..A....... ..........  [4080]
#L1MdF_II     .......... .......... .......... ..T....... .......... .......... ..A....... ..........  [4080]
#L1MdF_III    .......... .......... .......... ..T....... .......... .......... ..A....... ..........  [4080]
#L1MdN_I      .......... .......... .......... .......... .......... .......... ..A....... ..........  [4080]
#L1MdA_VII    .......... .......... .......... .......... .......... .......... .......... ..........  [4080]
#L1MdMus_I    .......... .......... .......... ..T....... .......... .......... ..A....... ..........  [4080]
#L1MdFanc_I   .....T.... .......... .......... ..T....... .......... .......... ..A....... ..........  [4080]
#L1MdV_I      .......... .......... .......... ..T....... .......... .......... ..A....... ..........  [4080]
#L1MdFanc_II  .......... .......... .......... ..T.....A. .......... .......... ..A....... ..........  [4080]
#L1MdMus_II   .......... .......... ....H..... ..T....... .......... .......... ..A....... ..........  [4080]
#L1MdV_II     .......... ....H..... .......... ..T.....A. .......... .......... ..AR...... ....Y.....  [4080]
#L1Lx_I       .......... .H........ .......... ..T.....A. .......... H......... D.A...AC.. .N........  [4080]
#L1Lx_III     .....HN... .......... .......... ..T.....A. .......... .......... HRA.....S. ..........  [4080]
#L1Lx_II      .....N.... .......... .......... ..T.....D. .......... .......... ..A....... ..........  [4080]
#L1Lx_IV      .......... ........R. .......... ..T.....A. .......... .......... ..A.....N. ..........  [4080]
#L1MdV_III    .......... ....A..... .......... ..T.....A. .......... .......... ..A....... .......T..  [4080]
```

```
#L1MdA_I      CAATTGGTGC TGGCACAACT GGTTGTTATC GTGTAGAAGA ATGCGAATCG ATCCATACTT ATCTCCTTGT ACTAAGGTCA  [4160]
#L1MdA_II     .......... .......... .......... .......... .......... .......... .......... ..........  [4160]
#L1MdA_III    .......... .......... .......... A......... .......... .......... .......... ..........  [4160]
#L1MdA_IV     ...W...... .......... .......... A......... .......... ........C. .......... ..........  [4160]
#L1MdGf_I     .......... .......... .......... A......... .......... .......... .......... ..........  [4160]
#L1MdGf_II    .......... .......... .......... A......... .......... .......... .......... ..........  [4160]
#L1MdTf_I     .......... .......... .......... A......... .......... .......... .......... ..........  [4160]
#L1MdTf_II    .......... .......... .......... A......... .......... .......... .......... ..........  [4160]
#L1MdTf_III   ...A...... .......... .......... A......... .......... .......... .......... ..........  [4160]
#L1MdA_V      ...A...... .......... .......... A......... .......... .C....T.C. C......... ..........  [4160]
#L1MdA_VI     ...A...... .......... ...G...... A......... ........T. ......T.C. .......... ..........  [4160]
#L1MdF_V      ...A...... .......... ..CG...... A......... ........T. ......T.C. .......... ..........  [4160]
#L1MdF_IV     ...A...... .......... ..CG...... A......... ........T. ......T.C. .......... ..........  [4160]
#L1MdF_I      ...A...... .......... ..CG...... A......... ........T. ......TTC. .......... ..........  [4160]
#L1MdF_II     ...A...... .......... ..C....... A......... T.K.....T. .....KTAC. .......... ..........  [4160]
#L1MdF_III    ...A...... .......... ..CG...... A......... ...N....T. ......T.C. .......... ..........  [4160]
#L1MdN_I      ...A...... .......... .......... A......... .......... .C....T.C. C......... ..........  [4160]
#L1MdA_VII    ...A...... .......... ...G...... A......... ........T. ......T.C. .......... ..........  [4160]
#L1MdMus_I    ...A...... .......... ..CG...... A......... ........T. ......T... .......... ..A...C...  [4160]
#L1MdFanc_I   ...A...... .......... ..CG...... A......... ........T. ......T... .......... ..A...C...  [4160]
#L1MdV_I      ...A...... .......... ..CG...... A......... ........T. ......T... .......... ..........  [4160]
#L1MdFanc_II ...A...... ....T..... ..CG...... A......... ........T. ......T... .......... ..A...C...  [4160]
#L1MdMus_II   H..A...... .......... ..CG...... A......... ........T. ......W... .......... ..W...S...  [4160]
#L1MdV_II     ...A...... ....T..... ..CG....D. A......... ........T. ......T... .......... ..A...C...  [4160]
#L1Lx_I       ...A..R... ....T..... ..CG....B. A......... ........T. ......T... .......... ..A...C...  [4160]
#L1Lx_III     ...A...... ...YT..... ..CG..Y.G. A......... ..D.A..... ......T... .......... ..A...C...  [4160]
#L1Lx_II      ...A...... ...YT..... ..CG..Y.D. A......... ....A...NV ..B...T... .......... ..A...N...  [4160]
#L1Lx_IV      ...A..N... ...TT..... ..CA..C.A. A......... ....A...T. ......T... ......C... ..A...C...  [4160]
#L1MdV_III    ...A...... ...YT..... ..CG..CGG. A.......A. ....A...T. .C....T... .......... ..A...C...  [4160]

#L1MdA_I      AATCTAAGTG GATCAAGGAA CTTCACATAA AACCAGAGAC ACTGAAACTT ATAGAGGAGA AAGTGGGGAA AAGCCTTGAA  [4240]
#L1MdA_II     .......... .......... .......... .......... .......... .......... .......... ..........  [4240]
#L1MdA_III    .......... .......... .......... .......... .......... .......... .......... ..........  [4240]
#L1MdA_IV     .......... .......... .......... .......... .......... .......... .......... ..........  [4240]
#L1MdGf_I     .......... .......... .......... .......... .......... .......... .......... ..........  [4240]
#L1MdGf_II    .......... .......... .......... .......... .......... .......... .......... ..........  [4240]
#L1MdTf_I     .......... .......... .......... .......... .......... .......... .......... ..........  [4240]
#L1MdTf_II    .......... .......... .......... .......... .......... .......... .......... ..........  [4240]
#L1MdTf_III   .......A.. ......A... .......... .......... .......... .......... .......... ..........  [4240]
#L1MdA_V      .......... .......... .......... .......... .......... .......... .......... ...Y..C...  [4240]
#L1MdA_VI     .......... .........G ..C....... .......... .......... .......... .......... ......C...  [4240]
#L1MdF_V      .......... .......... ..C....... .......... .S........ .......... .......... ......C...  [4240]
#L1MdF_IV     .......... ...Y...... ..C....... .......... .S........ .......... .......... ......C...  [4240]
#L1MdF_I      .......... ...T...... ..C....... .......... .......... .......... ....A..... ......C...  [4240]
#L1MdF_II     .......... ...T.....W ..C....... .......... .......... .......... ....A..... ......C...  [4240]
#L1MdF_III    .......... ...T...... ..C....... .......... .......... .......... ....A..... ......C...  [4240]
#L1MdN_I      .......... .......... ..Y....... .......... .......... .......... .......... ...T..C...  [4240]
#L1MdA_VII    .......... .......... ..C....... .......... .......... .......... .......... ......C...  [4240]
#L1MdMus_I    .G........ ......A... ..C....... .......... .......T.. .......... .......... ..B...C...  [4240]
#L1MdFanc_I   .G........ ......A..C ..C....... .......... .......T.A .......... .......... ......C...  [4240]
#L1MdV_I      .R........ .......... ..C....... .......... .......... .......... .......... ......C...  [4240]
#L1MdFanc_II .G........ .......... ..C....... .......... .......... .......... .......... ......C...  [4240]
#L1MdMus_II   .D........ .......... ..C....... .......... .......... .......... ........R. ......C...  [4240]
#L1MdV_II     .G........ ......R... ..C....... .......... .......... .......... .......... G.....C...  [4240]
#L1Lx_I       .G........ .......... ..C....... .......... .......... .......... ....R..... G.....CV..  [4240]
#L1Lx_III     .G..C....D .........H ..C....... .......T.. .........W .....AR... ....D..... G.N...C..R  [4240]
#L1Lx_II      .R..C..... .........H ..C....... .......T.. ......H..N .....AR... .......... V.....C..G  [4240]
#L1Lx_IV      .G..C..... .........C ..C....... .......T.. ......T..A .....A.... .......... ...T..C...  [4240]
#L1MdV_III    .G..C..... .........C ..C....... .......D.. .......... .......... .....D.... G.....C...  [4240]

#L1MdA_I      GATATGGGCA CAGGGGAAAA ATTCCTGAAC AGAACAGCAA TGGCTTGTGC TGTAAGATCG AGAATCGACA AATGGGACCT  [4320]
#L1MdA_II     .......... .......... .......... .......... .......... .......... .....T.... ..........  [4320]
#L1MdA_III    .......... .......... .......... .......... .......... .......... .....T.... ..........  [4320]
#L1MdA_IV     .......... .......... .........Y .......... .......... .......... .....T.... ..........  [4320]
#L1MdGf_I     ........T. .........G .......... .......... .......CT. .......... .....T.... ..........  [4320]
#L1MdGf_II    .......... .......... .......... .......... .......... .......... .....T.... ..........  [4320]
#L1MdTf_I     .......... .......... .......... .......... .......... .......... .....T.... ..........  [4320]
#L1MdTf_II    .......... .......... .......... .......... .......... .......... .....T.... ..........  [4320]
#L1MdTf_III   .......... .......... .......... .......... .......... .......... .....T.... ..........  [4320]
#L1MdA_V      .......... .....A.... .........T .......... .......... .......... ..G..T.... ..........  [4320]
#L1MdA_VI     .....K.... .......... .........T .......... .......... .......... .....T.... ..........  [4320]
#L1MdF_V      .......... .......... .........T .......... .......... .......... .......... ..........  [4320]
#L1MdF_IV     .......... .......... .........T .......... .......... .......... .......... ..........  [4320]
#L1MdF_I      ........T. .......... .........T .......... .......... .........A ........T. ..........  [4320]
#L1MdF_II     ........T. .......... .........T .......... .......... .......... .....Y..T. ..........  [4320]
#L1MdF_III    ........T. .......... .........T .......... .......... .......... ........T. ..........  [4320]
#L1MdN_I      .......... .....A.... .........T .......... .......... .T........ ..G..T.... ..........  [4320]
#L1MdA_VII    .......... .......... .........T .......... .......... .......... .....T.... ..........  [4320]
#L1MdMus_I    .......... .......... ......A... .......... ....C..... .........A ......V... ..........  [4320]
#L1MdFanc_I   .......... .......... ......A... .......... .......... .........A .....T.... ..........  [4320]
#L1MdV_I      .......... .......... .........T .......... .......... .......... .....T.... ..........  [4320]
#L1MdFanc_II .......... .......... .......... .......... .....N.... .......... .......... ..........  [4320]
#L1MdMus_II   .......... ....V..... .......... .......... .......... .......... .......... ..........  [4320]
#L1MdV_II     .......... .........R .......... ......S... .......... .........A .......... ..........  [4320]
#L1Lx_I       B.Y....... .......... .......... ......C... .......... .........A ..D....... ..........  [4320]
#L1Lx_III     S.C....... .....D...W T........H ......C... .R....A... .C.......A .....T.... ..........  [4320]
#L1Lx_II      C.C....... .......... T......... ......C... .D....A... .N.......A .....T.... ..........  [4320]
#L1Lx_IV      C.C....... .......... G......... ......C... ......A... .C.......A .....T.... ..........  [4320]
#L1MdV_III    C.Y....... .......... .........B ......C... ......R... .........A ......A... ..........  [4320]
```

```
#L1MdA_I     AATGAAACTC CAAAGTTTCT GCAAGGCAAA AGACACCGTC AATAAGACAA AAAGACCACC AACAGATTGG GAAAGGATCT  [4400]
#L1MdA_II    .......... .......... .......... .......... .......... .......... .......... ..........  [4400]
#L1MdA_III   .......... .......... .......... .......... .......... .......... .......... ..........  [4400]
#L1MdA_IV    C......... .....C.... .......... .......... .......... .......... .......... ..........  [4400]
#L1MdGf_I    .......... .......... .......... .......... ...G...... .......... .......... ..........  [4400]
#L1MdGf_II   .......... .......... .......... .......... .......... .......... .......... ..........  [4400]
#L1MdTf_I    .......... .......... .......... ......T... T......... .......... ......C... ..........  [4400]
#L1MdTf_II   .......... .......... .......... ......T... T......... .......... ......C... ..........  [4400]
#L1MdTf_III  .......... .......... .......... .......... .......... .G........ .......... ..........  [4400]
#L1MdA_V     C........G .....C.... .......... .......... .......... .......... .......... ..........  [4400]
#L1MdA_VI    C........G .....C.... .T........ .......... .......... ....G..... .......... ..........  [4400]
#L1MdF_V     C..A...T.G .....C.... .T........ .......... .......... ....G..... .......... ..........  [4400]
#L1MdF_IV    C..A...T.G .....C.... .T........ .......... .......... ....G..... .......... ..........  [4400]
#L1MdF_I     C..A...T.G .....C.... ....A..... .......... .......... ....G..... .......... ..........  [4400]
#L1MdF_II    C..A...T.G .....C.... .......... .......... .......... .......... .......... ..........  [4400]
#L1MdF_III   C..A..GT.G .....C.... .......... .......... .......... .......... .......... ..........  [4400]
#L1MdN_I     C........G .....C.... .......... .......... .......... .......... .......... ..........  [4400]
#L1MdA_VII   C........G .....C.... .......... .......... .......... ....G..... .......... ..........  [4400]
#L1MdMus_I   C..A...T.G .....C.... .T.G...... ...T..T... .......... ....G..... .......... .......A..T.  [4400]
#L1MdFanc_I  C..A...T.G .....C.... .T........ ......T... .......... ....G..... .......... ........T.  [4400]
#L1MdV_I     C..A...T.G .....C.... .T........ ......T... .......... ....G..... .......... ..........  [4400]
#L1MdFanc_II C..A...T.G .....C.... .T........ ......T... .......... ....G..... .......... ..........  [4400]
#L1MdMus_II  C..A...T.G .....C.... .T........ .......... .......... ....G..... .......... ..........  [4400]
#L1MdV_II    C..A...T.G .....C.... .T........ G.....T... ........M. ....G.M.Y. .......... ....A.....  [4400]
#L1Lx_I      C..A...T.G .....C.... .T........ G.....T... .......... ....G.V... .......... ....A.....  [4400]
#L1Lx_III    C..A...T.G .....C.... .T........ G.....T... ....GR.... ..T.G.A... .......... ....A.....  [4400]
#L1Lx_II     C..A...T.G .....C.... NT........ G.....T... ....G..... ..C.G.A... .......... ....A.....  [4400]
#L1Lx_IV     C..A...T.G .....C.... .T........ G.....T... ....G..... ..C.G.A... .......... ....A.....  [4400]
#L1MdV_III   C..A...T.G .....C.... .T........ G.....T... ....G..... ....G.A... .......... ....A...H.  [4400]

#L1MdA_I     TTACCTATCC TAAATCAGAT AGGGGACTAA TATCCAACAT ATATAAAGAA CTCAAGAAGG TGGACTTCAG AAAATCAAAT  [4480]
#L1MdA_II    .......... .......... .......... .......... .......... .......... .......... ..........  [4480]
#L1MdA_III   .......... .......... .......... .......... .......... .......... .......... ..........  [4480]
#L1MdA_IV    .......... .......... .......... .......... .......... .......... .......... ..........  [4480]
#L1MdGf_I    .......... .......... .......... .......... .......... .......... .......... ..........  [4480]
#L1MdGf_II   .......... .......... .......... .......... .......... .......... .......... ..........  [4480]
#L1MdTf_I    .......... .......... .......... .......... .......... .......... .....C.... ..........  [4480]
#L1MdTf_II   .......... .......... .......... .......... .......... .......... .....C.... ..........  [4480]
#L1MdTf_III  .......... .......... .......... .......... .......... .......... .......... .........Y  [4480]
#L1MdA_V     .........T .......... .......... .......T.. .......... .......G.. ......C... ..........  [4480]
#L1MdA_VI    .......... .......... ..R....... .......T.. .......... .......... ......C... ..........  [4480]
#L1MdF_V     .......... .......... .......... .......T.. .......... .......... ......C... ..........  [4480]
#L1MdF_IV    .......... .......... .......... .......T.. .......... .......... ......C... ..........  [4480]
#L1MdF_I     .......... C.....G... .......... .......T.. .......... .......... ......C... ..........  [4480]
#L1MdF_II    .......... C.....G..C .......... .......T.. .......... .......... .A....C..T ..........  [4480]
#L1MdF_III   .......... .......... .......... .......T.. .......... .......... ......C... ..........  [4480]
#L1MdN_I     .........T .......... .......... .......T.. .......... .......G.. ......C... ..........  [4480]
#L1MdA_VII   .......... .......... .......... .......T.. .......... .......... ......C... ..........  [4480]
#L1MdMus_I   .....A.... ......T... .......... .......T.. ...C.....G .........C ..A...C... ...T......  [4480]
#L1MdFanc_I  .....A.... ......T... .......... .......T.. ...C.....G .........C ......C... ...T......  [4480]
#L1MdV_I     .....A.... .......... .......... .......T.. .......... .........V ......C... ....H.....  [4480]
#L1MdFanc_II .....A.... ......C... .......... .......T.. ...C...... .........C ......C... ..........  [4480]
#L1MdMus_II  .....A.... .......... .......... .......W.. .......... .........V .C....C... ..........  [4480]
#L1MdV_II    ....HA.... ......GN.. .....V.... .......T.. .......... .........T ......C... ....Y.....  [4480]
#L1Lx_I      .....A.... ......C... .....D.... .......T.. ...Y...... .........T .D....C... ....Y.....  [4480]
#L1Lx_III    .....A.C.. ..C...C... ..AV.G.... .......T.. ...C...... .........T .A....C... .G..H.....  [4480]
#L1Lx_II     .....A.Y.. ..C...C... ..A..G.... .......T.. ...C...... ......N..DT .A....C... .G..N.....  [4480]
#L1Lx_IV     .....A.... ..C...T... ..A..G.... .......T.. ...C...... .........T .A....C... .G..C.....  [4480]
#L1MdV_III   .....A.... ..C...C... ..A..G.... .......T.. ...C...... .........T .A....C... .G..C.....  [4480]

#L1MdA_I     AACCCCATTA AAAAATGGGG CTCAGAACTG AACAAAGAAT TCTCACCTGA GGAATACCGA ATGGCAGAGA AGCACTTGAA  [4560]
#L1MdA_II    .......... .......... .......... .......... .......... .......... .......... .....C....  [4560]
#L1MdA_III   .......... .......... .......... .......... .......... .......... .......... .....C....  [4560]
#L1MdA_IV    .......... .......... .......... .......... .......... .......... .......... .....C....  [4560]
#L1MdGf_I    .......... .......... .......... .......... .......... .......... .......... .....C....  [4560]
#L1MdGf_II   .......... .......... .......... .......... .......... .......... .......... .....C....  [4560]
#L1MdTf_I    ......C... .......... .......... .......... .......... .......... .......... .....C....  [4560]
#L1MdTf_II   ......C... .......... .......... .......... .......... .......... .......... .....C....  [4560]
#L1MdTf_III  .......... .......... .......... .......... .......... .......... .......... .....C....  [4560]
#L1MdA_V     ......C... ...G...... ......G... .......... .......C.. .......... .A...T.... .A...C....  [4560]
#L1MdA_VI    .......... .......... ......G..A .......... .......... .......... .....T.... .A...C....  [4560]
#L1MdF_V     .......... .......... ......G..A .......... .......... .......... .....T.... .....C....  [4560]
#L1MdF_IV    .......... .......... ......G..A .......... .......... .......... .....T.... .....C....  [4560]
#L1MdF_I     .......... .......... ......G... .......... .......... .......... .......... .....C....  [4560]
#L1MdF_II    .......... .......... .......... ........T. .......C.. .......... .......... .....C....  [4560]
#L1MdF_III   .......... .......... ......G... .......... .......... .......... .......... .....C....  [4560]
#L1MdN_I     .G....C... ...G...... ......G... .......... .......G.. .......... .A...T.... .A...C....  [4560]
#L1MdA_VII   .......... .......... ......G..A .......... .......... .......... .....T.... .....C....  [4560]
#L1MdMus_I   .......... .......... TA....G..A .......... .....A.... .......... .G...T.... .....C....  [4560]
#L1MdFanc_I  .......... ....WB.... TA....G..A .......... .....A.... .......... .....T.... .....C....  [4560]
#L1MdV_I     .......... .......... .A....G..A .......... .....A.... ......Y... .....T.... .....C....  [4560]
#L1MdFanc_II .......... .......... TA....G..A .......... .....A.... .......... .....T.... .....C....  [4560]
#L1MdMus_II  .....Y.... .......V.. TA....V..A ......D... .H...AV... .......... .....T.... ....YC....  [4560]
#L1MdV_II    .....T.... .......... TA....G..A .......... .....A.... .......... .....T.... .....C.R..  [4560]
#L1Lx_I      .....T.... .......... TA....G..A ..V....... .....A.... ......H... .....T.... .....C.A..  [4560]
#L1Lx_III    .....T.... ........R. TA....G..A .......... .....A.... ......H... .....T.... .....C.A..  [4560]
#L1Lx_II     .....T.... .......... TA....G..A .......... .....A.... ......Y... .....T.... .....C.A..  [4560]
#L1Lx_IV     .....T.... .......... AA....G..A ....G..... .....A.... ....ACT.A. .K...TV... .....C.A..  [4560]
#L1MdV_III   .....T.... .........R TA....G..A .......... .T...A.... .R....T.A. .....T.... .....C.A..  [4560]
```

```
#L1MdA_I      AAAATGTTCA ACATCCTTAA TCATCAGGGA AATGCAAATC AAAACAACCC TGAGATTCCA CCTCACACCA GTCAGAATGG  [4640]
#L1MdA_II     .......... .......... .......... .......... .......... .......... .......... ..........  [4640]
#L1MdA_III    .......... .......... .......... .......... .......... .......... .......... ..........  [4640]
#L1MdA_IV     .......... .......... .......... .......... .......... .......... .......... ..........  [4640]
#L1MdGf_I     ......C... .......... .......... .......... .......... .......... .......... ..........  [4640]
#L1MdGf_II    .......... .......... .......... .......... .......... .......... .......... ..........  [4640]
#L1MdTf_I     .......... .......... .......... .......... .......... .......... .......... ..G.......  [4640]
#L1MdTf_II    .......... .......... .......... .......... .......... .......... .......... ..G.......  [4640]
#L1MdTf_III   .......... .......... .......... .......... .......... .......... .......... .........T  [4640]
#L1MdA_V      .......... .......... .......A.. .......... .......... T......... .......... ..........  [4640]
#L1MdA_VI     .......... G......... .......... .......... .......... T......... .......... ..........  [4640]
#L1MdF_V      .......... G......... .......... .......... .......... Y......... .......... ..........  [4640]
#L1MdF_IV     .......... R......... .......... .......... .......... .......... .......... ..........  [4640]
#L1MdF_I      .......... .......... .......... .......... ........A. .......... .T....T... ..........  [4640]
#L1MdF_II     .......... .......... .......... .......... .......... .......... .......... ..........  [4640]
#L1MdF_III    .......... .......... .......... .......... .......... .......... .......... ..........  [4640]
#L1MdN_I      .......... .......... .......A.. .......... .......... T......... .......... ..........  [4640]
#L1MdA_VII    ......Y... G......... .......A.. .......... .......... T......... .......... ..........  [4640]
#L1MdMus_I    .......... .......... .......... .......... .......... .......... .......... ..........  [4640]
#L1MdFanc_I   .......... .......... .......... .......... .........T .......... .......... ..........  [4640]
#L1MdV_I      .......... .......... .......... .......... .......... .......... .......... ..........  [4640]
#L1MdFanc_II  .......... .......... .......... .......... .......... .......... .......... ..........  [4640]
#L1MdMus_II   .......... .......... .......... .......... .......... .......... ........H. ..........  [4640]
#L1MdV_II     .......... .......... .......... .......... .......... .......... .......... ..........  [4640]
#L1Lx_I       R......... ....Y....R .......... .......... .......... .......... .......B.. ..........  [4640]
#L1Lx_III     G......... .........G .......... .......... .......... .......... .......... ..........  [4640]
#L1Lx_II      G......... .........N .......... .......... .......... .......... .....H.... ..........  [4640]
#L1Lx_IV      G......... .........G .......... .......D.. .......... .......... .......... ..........  [4640]
#L1MdV_III    G......... .........G .......... .......... .......... .......... .......... ..........  [4640]

#L1MdA_I      CTAAGATCAA AAATTCAGGT GACAGCAGAT GCTGGCGAGG ATGTGGAGAA AGAGGAACAC TCCTCCATTG TTGGTGGGAG  [4720]
#L1MdA_II     .......... .......... .......... .......T.. .......... .......... .......... .........K  [4720]
#L1MdA_III    .......... .......... .......... .......T.. .......... .......... .......... .........T  [4720]
#L1MdA_IV     .......... .......... .......... .......... .......... .......... .......... .........T  [4720]
#L1MdGf_I     .......... .......... .......... .......T.. .......... .......... .......... .........T  [4720]
#L1MdGf_II    .......... .......... .......... .......T.. .......... .......... .......... .........T  [4720]
#L1MdTf_I     .......... .......... .......... .......... .......... .......... .......... .........T  [4720]
#L1MdTf_II    .......... .......... .......... .......... .......... .......... .......... .........T  [4720]
#L1MdTf_III   .......... .......... .......... .......... .......... .......... .......... .........T  [4720]
#L1MdA_V      .......... .......... .......... .......... .......... .......... .......... .........T  [4720]
#L1MdA_VI     .......... .......... .......... .......... .......... .......... .......... .........T  [4720]
#L1MdF_V      .......... .......... .......... .....H.... .......... .......... .......... .........T  [4720]
#L1MdF_IV     .......... .......... .......... .......... .......... .......... .......... .........T  [4720]
#L1MdF_I      .......... ...C...... .......... .......... .......... ..G....... .......... .........T  [4720]
#L1MdF_II     .......S.. .......... .......... .......... .......... .......... .......... .........T  [4720]
#L1MdF_III    .......... .......... .......... .......... .......... .......... .......... .........T  [4720]
#L1MdN_I      .......... .......... .......... .......... .......... .......... .......... .........T  [4720]
#L1MdA_VII    .......... .......... .......... .......... .......... .......... ....Y..... .........T  [4720]
#L1MdMus_I    ...G...... .......... .......... .......... .......... .......... .......... C........T  [4720]
#L1MdFanc_I   .......... .......... .......... .......... .......... .......... .......... C........T  [4720]
#L1MdV_I      .......... .......... .......... .......... .......... .......... .......... .........T  [4720]
#L1MdFanc_II  .......... .......... .......... .......... ......D... .......... .......... C........T  [4720]
#L1MdMus_II   .......... .......... .......... ......R... .......... .....R.... .......... Y........T  [4720]
#L1MdV_II     .......... ...C...... .......... .......... .......... .......... .......... C........Y  [4720]
#L1Lx_I       .......M.. ...C...... .......... .......... .......... .......... .......... C........T  [4720]
#L1Lx_III     .......... ...C...... .......... .......... .......... .......... .......... H........T  [4720]
#L1Lx_II      .......... ...C...... .......... .......... ....V..... .......... .......... Y.......DT  [4720]
#L1Lx_IV      .......... ...C...... .......... ......A... .......... .......... .......... H........T  [4720]
#L1MdV_III    .......... ...C....R. .......... .......... .......... .......... .......... Y........T  [4720]

#L1MdA_I      TGCAGGCTTG TACAACCACT CTGGAAATCA GTCTGGCGGT TCCTCAGAAA ACTGGACATA GTACTACCGG AGGATCCAGC  [4800]
#L1MdA_II     .......... .......... .......... .......... .......... .T........ .......... ..........  [4800]
#L1MdA_III    .......... .......... .......... .......... .......... .T........ .......... ..........  [4800]
#L1MdA_IV     .......... .......... .......... .......... .......... .T........ .......... ..........  [4800]
#L1MdGf_I     .......... .......... .....G.... .......... .......... ......T... .......... ..........  [4800]
#L1MdGf_II    .......... .......... .......... .......... .......... .T........ .......... ..........  [4800]
#L1MdTf_I     .......... .......... .......... .......... .......... .T........ .......... ..........  [4800]
#L1MdTf_II    .......... .......... .......... .......... .......... .T........ .......... ..........  [4800]
#L1MdTf_III   .......... .......... .........C .......... .......... .T........ .......... ..........  [4800]
#L1MdA_V      ....A..... .......... .......... .......... .......... .T........ .......... .......H..  [4800]
#L1MdA_VI     ....A..... .......... .......... .......... .......... .T.......R .......... .......C..  [4800]
#L1MdF_V      ....A..... .......... .......... .......... .......... .T........ .......... .......C..  [4800]
#L1MdF_IV     ....A..... .......... .......... .......... .......... .T........ .......... .......C..  [4800]
#L1MdF_I      ....A..... .......... .......... .......... .......... .T........ .......... .......C..  [4800]
#L1MdF_II     ....A..... .......... .......... .......... .......... .T........ .......... .R........  [4800]
#L1MdF_III    ....A..... .......... .......... .......... .......... .T........ .......... .......C..  [4800]
#L1MdN_I      ....A..... .......... .......... .......... .......... .T........ .......... .......C..  [4800]
#L1MdA_VII    ....A..... .......... .......... .......... .......... .T........ .......G.. .......C..  [4800]
#L1MdMus_I    ....A...K. .......... .......... .......... .......... .......... .........V TA........  [4800]
#L1MdFanc_I   ....A..... .......... ......G... .......... .......... .T........ A......... .A........  [4800]
#L1MdV_I      ....A..... .......... .......... .......... .......... .T........ .......... ..........  [4800]
#L1MdFanc_II  ....A...G. .......... .......... .......... .......... .T........ .......... .A........  [4800]
#L1MdMus_II   ....AD..K. .......... .......... .......... .......... .T........ ......Y... .R.......Y  [4800]
#L1MdV_II     ....A...G. .......... .......... ..T....... .......... .T........ .......... .A........  [4800]
#L1Lx_I       ....A...G. .......... .......... ..T....... .......... .T........ .......GD. .R........  [4800]
#L1Lx_III     ....A...G. .......... .......... ..T....... .......... .T.R...... .......YC. ....C.....  [4800]
#L1Lx_II      ....A...G. .......... .......... ..H....... .......... .T..D..... ........T. ....CH....  [4800]
#L1Lx_IV      ....A...G. .......... .......... ..T....... .......... .T........ ........T. ....C.....  [4800]
#L1MdV_III    ....A...G. .......... .......... ..T.V.T... .......... .T........ ........T. ....C.....  [4800]
```

```
#L1MdA_I      AATACCTCTC CTGGGCATAT ATCCAGAAGA TGCCCCAACA GGTAAGAAGG ACACATGCTC CACTATGTTC ATAGCAGCCT  [4880]
#L1MdA_II     .......... .......... .......... .........T .......... .......... .......... ..........  [4880]
#L1MdA_III    .......... .......... .......... .........T .......... .......... .......... ..........  [4880]
#L1MdA_IV     .......... .......... .......... ..T......C .......... .......... .......... ..........  [4880]
#L1MdGf_I     .......... .......... .......... .........T .......... .......... .......... ..........  [4880]
#L1MdGf_II    .......... .......... .......... .........T .......... .......... .......... ..........  [4880]
#L1MdTf_I     .......... .......... .......... A........T .......... .......... .......... ..........  [4880]
#L1MdTf_II    .......... .......... .......... A........T .......... .......... .......... ..........  [4880]
#L1MdTf_III   .......... .......... .......... .........T .......... .......... .......... ..........  [4880]
#L1MdA_V      .......... .......... .......... ..T......C .......... .......... .......... ..........  [4880]
#L1MdA_VI     .......... .......... .......... ..TT.....T A.......A. .M........ .......... ..........  [4880]
#L1MdF_V      .......... .......... .......... ..TT.....C .......... .......... .......... ..........  [4880]
#L1MdF_IV     .......... .......... .......... ..TT.....C .......... .......... .......... ..........  [4880]
#L1MdF_I      .......... .......... .......... ..T......C .........A .......... .......... ..........  [4880]
#L1MdF_II     .........T .......... .......... .........C .......... .......... .......... ..........  [4880]
#L1MdF_III    .......... .......... .......... ..TY.....C .......... .......... .......... ..........  [4880]
#L1MdN_I      .......... .......... .......... ..T......C .......... .......... .......... ..........  [4880]
#L1MdA_VII    .......... .......... .......... ..T......C .......... .......... .......... ..........  [4880]
#L1MdMus_I    .......... .......... .C........ ..TT.....T .....T.... .......... .......... .........C  [4880]
#L1MdFanc_I   .......... .......... .C........ A.TT.....T .....T...A .......... .......... ..........  [4880]
#L1MdV_I      .......... .......... .......... ..TT.....T .....T...V .......... .......... ..........  [4880]
#L1MdFanc_II  .......... .......... .C........ ..TT.....T T....T.... .......... .......... ..........  [4880]
#L1MdMus_II   ...W...... .......... .C........ .RTT.....T T....T.... .......... Y......... ..........  [4880]
#L1MdV_II     ......W... .......... .H........ ..T......H T....T.... .......... .......... ..........  [4880]
#L1Lx_I       ......A... .......... .Y........ ..YT.....H T....T.... .......... .......... ..........  [4880]
#L1Lx_III     H.....A... .......... .C........ ...T...... T....T.... ......NY.. .......... ..........  [4880]
#L1Lx_II      T.....A... .......... .C........ ...T...... TD...T.... .......... .......... ..........  [4880]
#L1Lx_IV      T.....A... .......... .C........ ...T...... T....T.... .......... .......... .........D  [4880]
#L1MdV_III    T.....A... .......... .C........ ...T...... T....T.... .......... .......... ..........  [4880]

#L1MdA_I      TATTTATAAT AGCCAGAAGC TGGAAAGAAC CTAGATGCCC CTCAACAGAG GAATGGATAC AGAAAATGTG GTACATCTAC  [4960]
#L1MdA_II     .......... .......... .......... .Y........ .......... .......... .......... ..........  [4960]
#L1MdA_III    .......... .......... .......... .C........ .......... .......... .......... ..........  [4960]
#L1MdA_IV     .......... .......... .......... .C........ .......... .......... .......... ......T...  [4960]
#L1MdGf_I     .......... .......... .....G.... .C........ .......... .......... ....G..... ..........  [4960]
#L1MdGf_II    .......... .......... .......... .C........ .......... .......... .......... ..........  [4960]
#L1MdTf_I     .......... ........A. .......... .C........ .......... .......... .......... ..........  [4960]
#L1MdTf_II    .......... ........A. .......... .C........ .......... .......... .......... ..........  [4960]
#L1MdTf_III   .......... .......... .......... .C........ .......... .......... .......... ..........  [4960]
#L1MdA_V      .......... .......... .......... .C........ .......... .......... .A........ ......T...  [4960]
#L1MdA_VI     .......... .......... .......... .C........ .......... .......... .A........ ......T...  [4960]
#L1MdF_V      .......... .......... .......... .C.....Y.. .......... .......... .......... ......T...  [4960]
#L1MdF_IV     .......... .......... .......... .C........ .......... .......... .......... ......T...  [4960]
#L1MdF_I      .G........ .......... .......... .C........ .......... .......... .......... ......T...  [4960]
#L1MdF_II     .......... .......... .......... .C........ .......... .......... .......... ......T...  [4960]
#L1MdF_III    .......... .......... .......... .C........ .......... .......... .......... ......T...  [4960]
#L1MdN_I      .......... .......... .......... .C.....T.. .......... .......... .A........ ......T...  [4960]
#L1MdA_VII    .......... .......... .......... .C........ .......... .......... .A........ ......T...  [4960]
#L1MdMus_I    .......... .......... .......... .C.....T.. .......... .......... .......... ......T...  [4960]
#L1MdFanc_I   .......... .......... .......... .C.....T.. .........R .......... .......... ......T...  [4960]
#L1MdV_I      .......... .......... .......... .C.....T.. .......... .......... .......... ......T...  [4960]
#L1MdFanc_II  .......... .......... .......... .C.....T.. .......... .......... .......... ......T...  [4960]
#L1MdMus_II   ........V. .......... .......... .C.....T.. .......... .......... .......... ......T..Y  [4960]
#L1MdV_II     .......... .......... .......... .C.....T.. .......... .......... .......D.. ......T..H  [4960]
#L1Lx_I       .......... .......... .......... .C.....T.. .......... .......... .......... ......T...  [4960]
#L1Lx_III     .......... .......... .R........ .C.....T.. .......... .......... .......... ......T...  [4960]
#L1Lx_II      .......... .V........ ......V... .C.....T.. Y......... .......... .......... ......T...  [4960]
#L1Lx_IV      .......... .......... ......B... .C.....T.. .......... .......... .......... ......T...  [4960]
#L1MdV_III    .......... ......G... .......... .C.....T.. T.....C... .......... .......... ......T...  [4960]

#L1MdA_I      ACAATGGAGT ACTACTCAGC TATTAAAAAG AATGAATTTA TGAAATTCCT AGCCAAATGG ATGGACCTGG AGGGCATCAT  [5040]
#L1MdA_II     .......... .......... .......... .......... .......... .......... .......... ..........  [5040]
#L1MdA_III    .......... .......... .......... .......... .......... .......... .......... ..........  [5040]
#L1MdA_IV     .......... .......... .......... .......... .......... .......... .......... ..........  [5040]
#L1MdGf_I     .......... .......... .......... .......... .......... G......... .......... ..........  [5040]
#L1MdGf_II    .......... .......... .......... .......... .......... .......... .......... ..........  [5040]
#L1MdTf_I     .......... .......... .......... .......... .......... .......... .......... ..A.......  [5040]
#L1MdTf_II    .......... .......... .......... .......... .......... .......... .......... ..A.......  [5040]
#L1MdTf_III   .......... .......... .......... .......... .......... .......... .......... ..........  [5040]
#L1MdA_V      .......... .......... .......... .......... C......... ..G....... T......... ..........  [5040]
#L1MdA_VI     .......... .......... .......... .......... .......... ..G....... T......... .......T..  [5040]
#L1MdF_V      .......... .......... .......... .......... .......... ..G....... .......... ..........  [5040]
#L1MdF_IV     .......... .......... .......... .......... .......... ..G....... .......... ..........  [5040]
#L1MdF_I      ........A. .......... .........A .......... .......... ..G....... .......... ....T.....  [5040]
#L1MdF_II     .......... .......... .......... .......... .......... .......... .......... ..........  [5040]
#L1MdF_III    .......... .......... .......... .......... .......... ..G....... .......... ..........  [5040]
#L1MdN_I      .......... .......... .......... ........C. C......... ..G....... T......... ..........  [5040]
#L1MdA_VII    .......... .......... .......... .......... .......... ..G....... T......... ..........  [5040]
#L1MdMus_I    .......... .......... .........C .......... ........T. G.G....... ...T.T.... ...AT.....  [5040]
#L1MdFanc_I   .......... .......... .........C .......... ........T. G.G....... ...T.T.... ...AT.....  [5040]
#L1MdV_I      .......... .......... .........B .......... .......... ..G....... .....T.... ....T.....  [5040]
#L1MdFanc_II  .......... .......... .........C .......... ........T. ..G....... .....T.... ...AT.....  [5040]
#L1MdMus_II   .......... .......... A........C .........C. ........T. ..G.G..... ...N.T.... ...AT.....  [5040]
#L1MdV_II     .......... ....Y..... .........C .......... ........T. ..D....... .....T.... ...AT.....  [5040]
#L1Lx_I       .B........ .....D.... .........C .......... ........T. ..R....... .....T.... ...AT.....  [5040]
#L1Lx_III     ........V. ....N.N... .........C .......Y. ........T. ..G....... .....A..A. .ADAT.....  [5040]
#L1Lx_II      .H...D..R. .........H .........C .....N..Y. ........T. ..G....... .....A..AD .AAAT.....  [5040]
#L1Lx_IV      ........R. .......... .........C G....M..C. ........GC ..G....... .....A..T. .AAAT.....  [5040]
#L1MdV_III    .......... ........R. .........C G.......C. ........T. ..G....... H.A..A..A. .AAAT.....  [5040]
```

```
#L1MdA_I      CCTGAGTGAG GTAACACATT CACAAAGAAA CTCACACAAT ATGTATTCAC TGATAAGTGG ATATTAGCCC C-AAACCTAG  [5120]
#L1MdA_II     .......... .......... .......D.. .......... .....C.... .......... .......... .-........  [5120]
#L1MdA_III    .......... .......... .......G.. .......... .....C.... .......... .......... .-........  [5120]
#L1MdA_IV     .......... .......... .......G.. .......... .....C.... .......... .......... A-........  [5120]
#L1MdGf_I     .......... .......... .......G.. .......... .....C.... .......... .......... A-.......A  [5120]
#L1MdGf_II    .......... .......... .......G.. .......... .....C.... .......... .......... A-........  [5120]
#L1MdTf_I     .......... ........A. .......G.. .......... .....C.... .......... ...C...... A-........  [5120]
#L1MdTf_II    .......... ........A. .......G.. .......... .....C.... .......... ...C...... A-........  [5120]
#L1MdTf_III   .......... .......... .......G.. .......... .....C.... .......... .......... A-........  [5120]
#L1MdA_V      .......... .....C..A. ......AG.. ....A.TG.A .....C.... .......... .......... AG....T...  [5120]
#L1MdA_VI     .......... .....C..A. ......AG.. ....A.TG.. .....C.... .......... .......... AG....T...  [5120]
#L1MdF_V      .......... .....C..A. ......AG.. ......TG.. .....C.... .......... .......... AG.W..T...  [5120]
#L1MdF_IV     .......... .....C..A. ......AG.. ......TG.. .....C.... .......... .......... AG....T...  [5120]
#L1MdF_I      .........A .....C..A. .......G.. ...G...... .....C.... .......... .......... AG....T...  [5120]
#L1MdF_II     .......... .....C..A. .......G.. ...T...... .....C.... .......... .......... AG....T...  [5120]
#L1MdF_III    .......... .....C..A. .......G.. .......... .....C.... .......... .......... AG....T...  [5120]
#L1MdN_I      .......... .....C..A. ......AG.. ....A.TG.A .....C.... .......... ......A... AG....T...  [5120]
#L1MdA_VII    .......... ........A. ......AG.. ....A.TG.. .....C.... .......... .......... AG....T...  [5120]
#L1MdMus_I    .......... .....C..A. ......AG.. G....TTG.. ...C.C.... .......... .......... AG........  [5120]
#L1MdFanc_I   ...T...... .....C..A. ......AG.. G...TTAG.. ...C.C.... .......... .......... AG....A...  [5120]
#L1MdV_I      .......... .....C..A. ......AG.. ......TG.. ...Y.C.... .......... .......... AG....T...  [5120]
#L1MdFanc_II  .......... .....C..A. ......AG.. .A....TG.. ...C.C.... .......... .......... AG....T...  [5120]
#L1MdMus_II   .......... .....C..A. ......AG.. .A....TG.. ...C.C.... ......D... .......... AG....T...  [5120]
#L1MdV_II     .Y........ .....H..A. ......AG.. .A....TG.. ...C.C.... .......... .......... AG..D.TB..  [5120]
#L1Lx_I       .......... .....C..A. ......AG.. .A....TG.. ...C.Y.... .......... ......D... AG..R.TC..  [5120]
#L1Lx_III     .......... .....C..A. ......AG.. .A....TGG. ..NC.C...N .......... .......... AG..G.TCG.  [5120]
#L1Lx_II      .......... N....C..A. ......AG.. .A....TGG. ...Y.C.... .......... ......N.H. AG..G.TCG.  [5120]
#L1Lx_IV      .........D .....T..D. ......AG.. .A....TGG. .....C.... .......... .......... AA..GTTCG.  [5120]
#L1MdV_III    .......... .....C..DK ......AG.. HA....TGG. ...C.C.... .........R .......... AA..G.TCCA  [5120]

#L1MdA_I      GATACCCAAG ATATAAGATA TAATTTGCTA AACACATGAA ACTCAAGGAG AATGAAGACT GAAGTGTGGA CACTATGCCC  [5200]
#L1MdA_II     .......... .......... .......... .......... .......A.. .......... .......... ..........  [5200]
#L1MdA_III    ...W...... .......... C......... .......... .......A.. .......... .......... ..........  [5200]
#L1MdA_IV     .......... .......... C......... .......... .......A.. .......... .......... ..........  [5200]
#L1MdGf_I     .......... .......... C....C.... .......... .......A.A .......... .......A.. ..........  [5200]
#L1MdGf_II    .......... .......... C....C.... .......... .......A.A .......... .......... ..........  [5200]
#L1MdTf_I     ........C. .......... C....C.... .......... .......A.A .......... .......... ..........  [5200]
#L1MdTf_II    ........C. .......... C....C.... .......... .......A.A .......... .......... ..........  [5200]
#L1MdTf_III   .......... .......... C....C.... .......... .......A.A .......... .......... ..........  [5200]
#L1MdA_V      T...G.G.-. .......G.. C..KA...A. .......... ...G...A.. ..C......C A......... ....T...Y.  [5200]
#L1MdA_VI     A......... .......W.. C.......A. .......... .......A.. ..C......C A......... ....T.....  [5200]
#L1MdF_V      A......... ...Y...... C......YR. ...W...... .......A.. ..C......C A......... ....T.....  [5200]
#L1MdF_IV     A......... ...Y...... C.......G. .......... .......A.. ..C......C A......... ....T.....  [5200]
#L1MdF_I      .......... .......... C..C....C. ...G...... .T.....A.. ..C......C A......... ....T.A...  [5200]
#L1MdF_II     .......... .......... Y......... ..H...T... .......A-. ..C......C A......... ....T.....  [5200]
#L1MdF_III    .......... .......... C......... ...G...... .......A.. ..Y......C A......... ....K.....  [5200]
#L1MdN_I      T...G.G-.. .......G.. C...A..TA. ....T..... ...G...A.. ..C......C A......... ....T...T.  [5200]
#L1MdA_VII    T......G.. ........K. C......YA. .......... ...G...A.. ..C......C A......... ....T.....  [5200]
#L1MdMus_I    A......--- ----...... C..C..C.A. ......A... .A.....A.. ---......C A.C.C..... T...TCATT.  [5200]
#L1MdFanc_I   A.Y....--- ----...... C.......A. ......A... .A.....A.. .NG......C A.C.D..... T...TCATT.  [5200]
#L1MdV_I      A......--- ----...... C..B....A. .......... .......A.. ..C......C A......... ....TC....  [5200]
#L1MdFanc_II  A......--- ----...... C.......A. .......... .......A.. ..G......C A......... T...TCDTT.  [5200]
#L1MdMus_II   A...Y..--- ----...... C.......A. .......... .......A.. ..G......C A......... T...TC.YT.  [5200]
#L1MdV_II     A......--- ----...... C.......A. .N.......W .MWMMH.A.. ..G......C A......... T...TYVWT.  [5200]
#L1Lx_I       A......--- ----...... C.......A. .......... .......A.. ..G......C A......... T...TC.AT.  [5200]
#L1Lx_III     A......--- ----...... C....CA.AG .C..M..... G......A.. ..G.....YC A....R..V. T...TH.GT.  [5200]
#L1Lx_II      A......--- ----...... C....CABAG .C........ G......A.. ..G......C A......V.. T...TC.GT.  [5200]
#L1Lx_IV      A......--- ----.....W C....CA.AG .C..T..... D.C....A.. ..G......C A......... TG..TCAGTG  [5200]
#L1MdV_III    A...M..--- ----...... C....CA.AG .C........ G.....DA.. ..G......C A........G TG..TC.GT.  [5200]

#L1MdA_I      CTCCTTAGAT TTGGGAACAA AACACCCATG GAAGGAGTTA CAGAGACGGA GTTTGGAGCT GAGATGAAAG GATGGACCAT  [5280]
#L1MdA_II     .......... .......... .......... .......... .......AA. .......... .......... ..........  [5280]
#L1MdA_III    .........A .......... .......... .......... .......AA. .......... .......... ..........  [5280]
#L1MdA_IV     .........A .......... .......... .......... .......AA. .......... ....C..... ..........  [5280]
#L1MdGf_I     .........A G.....G... .......T.. .......... .......AA. ........Y. .....A.... ..........  [5280]
#L1MdGf_II    .........A G......... .......T.. .......... .......AA. .......... .......... .....T....  [5280]
#L1MdTf_I     .........A G......... .......... .......... ....A..AA. .......... .......... ..........  [5280]
#L1MdTf_II    .........A G......... .......... .......... ....A..AA. ..A....... .......... ..........  [5280]
#L1MdTf_III   .........A G......... .......T.. .......... .......AA. .......... .......... ..........  [5280]
#L1MdA_V      ..T......A ....A..... T-........ .......... .......AA. .......... ....CA.... ..........  [5280]
#L1MdA_VI     ..T......A .......... .......... .......... .......AA. .......... ....CA.... ..........  [5280]
#L1MdF_V      ..T......A .......... .......... .......... .......AA. .......... ....C..... ..........  [5280]
#L1MdF_IV     ..T......A .......... .......... .......... .......AA. .......... ....C..... ..........  [5280]
#L1MdF_I      T.T......A A......... .........D .......... .......AA. A......... .T..C..... ..........  [5280]
#L1MdF_II     ..T......A .A..A..... .......... .......... .......AA. A......AG. .T..C..... ..........  [5280]
#L1MdF_III    ..T......A .......... .......... .......... .......AA. .......... .T..C..... ..........  [5280]
#L1MdN_I      A.T......A ....A..... T-........ .......... .......AA. .......... ....CA.... .....T....  [5280]
#L1MdA_VII    ..T......A ....A..... T-........ .......... .......AA. .......... ....CA.... ..........  [5280]
#L1MdMus_I    ....C....A .A.....T.. ..T.T..... .........G .......AA. .......... A...C..... ..........  [5280]
#L1MdFanc_I   .........A .A........ ..T....... A......... .......AA. .......... A...C..... .......T..  [5280]
#L1MdV_I      ..T......A .......... .......... .......... .......AA. .......... ....C..... ..........  [5280]
#L1MdFanc_II  ..Y......A .G........ ..T.Y..... .......... .......AA. ...C...... ....C.G... ..A.......  [5280]
#L1MdMus_II   Y.Y......A .G........ ..T..Y.... .......... .......AA. ...C...... ......G..R ..AC..Y..Y  [5280]
#L1MdV_II     ..T......A DG........ ..T....... .......... .......AA. ...C.....A ...NCTG... ..AC......  [5280]
#L1Lx_I       ..T......A GG........ ..T....... .......... .......AA. ..DC.....A ....CTG... ..AT......  [5280]
#L1Lx_III     ..T......A VG........ ..T......D .G.....W.. .......AA. ..G......A ....CTG... ..AA.R....  [5280]
#L1Lx_II      ..T......A GD........ ..T.....C. .GVR...A.. .G.....AA. B.D.....NA ....CTG... V.AA.G....  [5280]
#L1Lx_IV      ..TH.....A GN.D...... ..T..T..CA .G....AA.. TG.....AA. ..R......A ....CTG... ..AA.G....  [5280]
#L1MdV_III    ..T......A GG-A...... ..T..T..CA .G..C.AA.. TG....TAA. ..G.A....A ....CTG... ..AA.G...C  [5280]
```

```
#L1MdA_I     GTAGAGACTG CCATAGCCAG GGATCCACCC CATAATCAGC ATCCAAACGC TGACACCATT GCATACACTA GCAAGATTTT  [5360]
#L1MdA_II    .......... .....T.... .......... .......... .......... .......... .......... ..........  [5360]
#L1MdA_III   .......... .....T.... .......... .......... .......... .......... .......... ..........  [5360]
#L1MdA_IV    S......... .....T.... .......... .......... T......... .......... .......... ..........  [5360]
#L1MdGf_I    .....C...A .....T..G. .......T.. .......... T......T.. .......... .......... ........A.  [5360]
#L1MdGf_II   .........K .....T.... .......... .......... .......... .......... .......... ..........  [5360]
#L1MdTf_I    .......... .....T.... .......... .......... .......... .......... .....T.... ..........  [5360]
#L1MdTf_II   .......... .....T.... .......... .......... T......T.. .......... .......... ..........  [5360]
#L1MdTf_III  .......... ..T..T.... .......... .......... .......... .......... .......... ..........  [5360]
#L1MdA_V     C......... .....T.... .......T.. .......T.. C........A .......... .......... .....CC...  [5360]
#L1MdA_VI    C......... .....T.... .......T.. .......... C......... .......... .......... ..........  [5360]
#L1MdF_V     C......... .....Y..G. .......T.. .......... C......... .......... .......... ..........  [5360]
#L1MdF_IV    C......... .....C..G. .......T.. .......... C......... .......... .......... ..........  [5360]
#L1MdF_I     C...T..T.. .....TG... .......T.. .......... T......T.. .......... .......... .........Y  [5360]
#L1MdF_II    C...T..T.. .....T.... A......T.. ...G...... T......... .......... .......... ..........  [5360]
#L1MdF_III   C......... .....T..G. .......T.. .......... T......Y.. .......... .......... ..........  [5360]
#L1MdN_I     C......... .....T.... .......T.. .......T.. C.....G..A .......... .......... .....CC...  [5360]
#L1MdA_VII   C......... .....T.... .......T.. .......T.. C........A .......... .......... .....CG...  [5360]
#L1MdMus_I   CC........ ..CC.C..G. ..G....T.. .......... CA........ A.....T... .....TG.C. ..........  [5360]
#L1MdFanc_I  CC.......A ..CC.C..G. .......T.. .......... CA......C. A.....T... .....TG.C. ..........  [5360]
#L1MdV_I     CC........ ..GC.C..G. .......T.. .......... CA........ A.....T... .....TG.C. ..........  [5360]
#L1MdFanc_II CC........ ..CC.C..G. .......T.. .....A..D. CA......C. A.....T... .....TG.C. ..........  [5360]
#L1MdMus_II  CC.......D Y.YC.C..G. .B.....T.. ....TA..A. CA.....YN. A.....YT... .....TG.C. .A........  [5360]
#L1MdV_II    CC........ ..CB.CHTG. .......T.. .....A..A. CA......C. AR....T..D .H.G.TG.C. A.....GCH.  [5360]
#L1Lx_I      CC........ ..CC.C.TG. .......T.. .....A..A. CA......C. A.....T... .H.G.TD.C. A.....KY..  [5360]
#L1Lx_III    CC........ ..CC.C.TG. .......T.. ....TA...T CA......C. A....NT... .TGG.TG.C. A....TGC..  [5360]
#L1Lx_II     CC.......N Y.CC.CVTG. .......T.. Y...TA...T YA......C. A.....T... .TGG.TGHC. AS...TGC..  [5360]
#L1Lx_IV     CC........ Y.CC.C.TG. .......T.. ....TA...Y CA......C. G...GYT... .TGG.TG.C. .G...TGC..  [5360]
#L1MdV_III   CC........ T.CC.C.TG. ....T..T.. ....TA...T TA......C. A.....T... .TGG.TG.C. AG...TGCA.  [5360]

#L1MdA_I     ATTGAAAGGA CGCAGATGTA GCTGTCTCTT GTGAGACTAT GCCGGGGCCC AGCAAACACA GAAGTGGATG CTCACAGTCA  [5440]
#L1MdA_II    .......... .C........ .......... .......... .........T .......... .......... ..........  [5440]
#L1MdA_III   .......... .C........ .......... .......... .........T .......... .......... ..........  [5440]
#L1MdA_IV    ..C....... .C........ .......... .......... .........T .......... .......... ..........  [5440]
#L1MdGf_I    GC........ .C.T...A.. .......... ..C...G... ...T.....T ........T. .......... .........G  [5440]
#L1MdGf_II   ..C....... .C........ .......... .......... .........T .......... .......... ..........  [5440]
#L1MdTf_I    ..C....... .C........ .......... .......... .........T .......... .......... ..........  [5440]
#L1MdTf_II   .C........ .C........ .......... .......... .........T .......... .......... ..........  [5440]
#L1MdTf_III  ..C....... .C........ .......... .......... .........T .......... .......... .C........  [5440]
#L1MdA_V     G...C..... .CGT...A.. ......Y... .........G .........T .......... .......... ..........  [5440]
#L1MdA_VI    GC........ .C.T...A.. .......... .......... .........T .......... K......... ..........  [5440]
#L1MdF_V     GC........ .C.W...A.. .......... .....R.... .........T .......... .......... ..........  [5440]
#L1MdF_IV    GC........ .C.T...A.. .......... .....D.... .........T .......... .......... ..........  [5440]
#L1MdF_I     GC........ .C.....A.. ...C...... .......... .........T .......... .......... A.........  [5440]
#L1MdF_II    GC........ .C.....A.. .......... .......... ...A.....T .......... .......... ..........  [5440]
#L1MdF_III   GC........ .C.....A.. .......... .......... .........T .......... .......... ..........  [5440]
#L1MdN_I     G...C..... ..GT...A.. ......C... .........G .........T .......... .......... ..........  [5440]
#L1MdA_VII   GC........ .C.T...A.. .......... .........G .........T .......... T......... ..........  [5440]
#L1MdMus_I   GC........ .C.T...A.. ........C. .....G..T. ...A.T...T G.....T... .......... ..........  [5440]
#L1MdFanc_I  GC....G... .C.T.N.A.. ........G. A....G.... ...A.T...T G.....T... .......... ..........  [5440]
#L1MdV_I     GC........ .C.T...A.. .......... .....G.... ...A.T...T G......... .......... ..........  [5440]
#L1MdFanc_II GC...C.... .C.T...A.. .......... .....G.... ...A.T...T G.....T... .......... ..........  [5440]
#L1MdMus_II  GC...C.... .C.T..YA.. ...V...... .....G.... ...ART...T G.....T... .......... B.........  [5440]
#L1MdV_II    NC...C.... GC.T...A.. ........C. .A.R.V..C. D..A.T...T GR....T... .......... ......R...  [5440]
#L1Lx_I      GC...C.... GC.T...A.. ........C. .A...G..C. ...A.T...T GR....T... .......... ..........  [5440]
#L1Lx_III    GC...C.... GC.T...A.. ........C. .A.R.G..C. D..A.T...T GA....T... ..G....... ...T...C..  [5440]
#L1Lx_II     GC...C.D.. GC.T...A.. .......BC. .A...G..C. D..A.TR..T GA....T... ..D....... B..N...C..  [5440]
#L1Lx_IV     GC...T..R. GC.T...A.G ........C. .A...G..C. ...A.A...T GA....T... ..G.C..... .......C..  [5440]
#L1MdV_III   GC........ GC.T...A.G ........C. .A...D.CC. ...A.A...T TA....T... ..G.C..... Y..G...C..  [5440]

#L1MdA_I     GCTAATGGAT GGATCATAGG G-CTCCCAAT GGAGGAGCTA GAGAAAGTAG CCAAGGAGCT AAAGGGATCT GCAACCCTAT  [5520]
#L1MdA_II    .......... .......... .-........ .......... .......... .......... .......... ..........  [5520]
#L1MdA_III   .......... ......C... .-........ .......... .......W.C .......... .......... ..........  [5520]
#L1MdA_IV    ....D..... ......C... .-........ .......... .........C .......... .......... ..........  [5520]
#L1MdGf_I    ....T..... ......C.T. .-.C...... .A........ .........C .A...A.... .......... ..........  [5520]
#L1MdGf_II   .......... ......C... .-........ .......... .........C .......... .......... ..........  [5520]
#L1MdTf_I    .......... ......C... .-........ .......... .........C .......... .......... T.........  [5520]
#L1MdTf_II   .......... ......C... .-........ .......... .........C .......... .......... T.........  [5520]
#L1MdTf_III  .......... ......C... .-........ .......... .........C .......... .......... ..........  [5520]
#L1MdA_V     A...T..... ......C... .-.C...... .......... .........T .......... ....A..... ..........  [5520]
#L1MdA_VI    ....T..... ......C... .-.C...... .......... .........C .......... .......... ..........  [5520]
#L1MdF_V     ....T..... ......C... .-.C...... .......... .........C .......... ......R... ..........  [5520]
#L1MdF_IV    ....T..... ......C... .-.C...... .......... .........C .......... ......R... ..........  [5520]
#L1MdF_I     ....T..... ..G...C.C. .-.C..T... .......... .......T.C .......... .......A.. ..........  [5520]
#L1MdF_II    ....T..A.. ...C..C... .-.C...... ......A... .......C.C .......A.. .......A.. ..........  [5520]
#L1MdF_III   ....T..... ......C... .-.C...... .......... .........C .......... .......... ..........  [5520]
#L1MdN_I     A...T..... ......C... .-.C...... .......... .........T .......... ....A..... ..........  [5520]
#L1MdA_VII   ....T..... ......C... .-.C...... .......... .........T .......... ....A..... ........G.  [5520]
#L1MdMus_I   T...TA.... ...A..C... .-.C...... .T........ .........C .....VV... G.....G... ..........  [5520]
#L1MdFanc_I  T...TAV... ...A..C... .-.C...... ....A..... .......C.C .......... G.....G... ..........  [5520]
#L1MdV_I     ....T..... ...A..C... .-.C...... .......... .........C .......... G.....G... ..........  [5520]
#L1MdFanc_II T...T..... ...A..C... .-.C...... .D........ .........C .......... N.....G... ..........  [5520]
#L1MdMus_II  T...T..... .D.A..C... .-.C..Y... .D........ .........C ........R.. R.....D... ..........  [5520]
#L1MdV_II    T.C.T....C ...G..C.D. .-TC...... .A........ .........C .......... G.....G.Y. .M.G...C..  [5520]
#L1Lx_I      T.Y.T....C ...G..C... .-TC...... .A........ .........C .......... G.....G.T. ...G...YM.  [5520]
#L1Lx_III    A.C.T....C ...G..C.D. .-TC...... ......R... .......G.C NV........ G....RGYT. ...G...C..  [5520]
#L1Lx_II     D.C.T....C T..G..C..N .-TC...... .......B.. .......G.C .......... D.....G.T. ...G...C..  [5520]
#L1Lx_IV     A.C.T..B.C T..G.GC..V .GTC...... .......W.D .....G.G.C TG........ G.G...G.T. ...G...C..  [5520]
#L1MdV_III   A.C.T....C T..G..CG.. .-TC...... A......T.. .......G.C TG......T. G.....G.T. .......C..  [5520]
```

```
#L1MdA_I     AGGTGGAACA ACATTATGAG CTAACCAGTA CCCC------ ---------- ---------- ---------- ---------G  [5600]
#L1MdA_II    .......... .........A .......... ....------ ---------- ---------- ---------- ---------.  [5600]
#L1MdA_III   .......... .........A .......... ....------ ---------- ---------- ---------- ---------.  [5600]
#L1MdA_IV    .......... .........A .......... ....------ ---------- ---------- ---------- ---------.  [5600]
#L1MdGf_I    .......... .........A .......... ....------ ---------- ---------- ---------- ---------.  [5600]
#L1MdGf_II   ...C...... .........A .......... Y...------ ---------- ---------- ---------- ---------.  [5600]
#L1MdTf_I    .......... .........A .......... ....------ ---------- ---------- ---------- ---------T  [5600]
#L1MdTf_II   .......... .........A .......... ....------ ---------- ---------- ---------- ---------T  [5600]
#L1MdTf_III  .......T.. .........A .......... ....------ ---------- ---------- ---------- ---------.  [5600]
#L1MdA_V     H......... .........A .......... ....------ ---------- ---------- ---------- ---------A  [5600]
#L1MdA_VI    .......... .........A .......... ....------ ---------- ---------- ---------- ---------.  [5600]
#L1MdF_V     .......... ...A.....A .......... ....CC---- ---------- ---------- ---------- ---------.  [5600]
#L1MdF_IV    .......... ...A.....A .......... ....CC---- ---------- ---------- ---------- ---------.  [5600]
#L1MdF_I     .......... ...A.....A .......... ...G------ ---------- ---------- ---------- ---------.  [5600]
#L1MdF_II    .......... ...A.....A ....G..... ....------ ---------- ---------- ---------- ---------.  [5600]
#L1MdF_III   .......... .........A .......... ....------ ---------- ---------- ---------- ---------D  [5600]
#L1MdN_I     C..A...... .........A .......... ....------ ---------- ---------- ---------- ---------A  [5600]
#L1MdA_VII   .....C.... .........A .......... ....------ ---------- ---------- ---------- ---------.  [5600]
#L1MdMus_I   .......... ...A.....A ....Y..... ....C----- ---------- ---------- ---------- ---------A  [5600]
#L1MdFanc_I  .......... ...A.....A .......... ....C----- ---------- ---------- ---------- ---------A  [5600]
#L1MdV_I     .......... ...A.....A .......... ....C----- ---------- ---------- ---------- ---------A  [5600]
#L1MdFanc_II ...A...... ...A.....A .......... ....CHC--- ---------- ---------- ---------- --------CA  [5600]
#L1MdMus_II  ...A.D.... ...A.....A .......... H...CC---- ---------- ---------- ---------- ---------A  [5600]
#L1MdV_II    ...A...... N..A.....A .......... H...CAGAGC TCCTTGGAAC TAAACCACCA ATCAAAGAAA ACACATGGTR  [5600]
#L1Lx_I      .D.A...V.. ...A.....A .......... ....CAGAGC TCCCTGGGAC TAAACCACCA ATCAAAGAAA ACACATGGT.  [5600]
#L1Lx_III    ...A...... -..A.....A .C........ ....CAGAGC TCCCAGGGAC TAAACCACCA ACCAAAGAGT ACACATGGA.  [5600]
#L1Lx_II     ...A...... ...D.....A .C........ H...CAGAGC TCCCAGGDAC TAAACCACCA ACNAACGAGT ACACATGGA.  [5600]
#L1Lx_IV     D.AG...G.. ...G.G.C.A .AGG...DAH H...CAGAGC TCCCAGGGAC TGGRCCACCA ACCAAAGART ACACATGGA.  [5600]
#L1MdV_III   ...AA..... ...A...C.A .C......AC H...CAGAGC TCCCAGGGAC TAAGCCATCA ACAAAGGAGT ACACATGG--  [5600]

#L1MdA_I     GAGCTCTTGA CTCTAGCTGC ATATATATCA AAAGATGGCC TAGTCGGCCA TCACTGGAAA GAGAGGCCCA TTGGACTTGC  [5680]
#L1MdA_II    .......... .......... .......... .......... .......... .......... .......... ..........  [5680]
#L1MdA_III   .......... .......... ....G..... .......... .......... .......... .......... ......AC..  [5680]
#L1MdA_IV    .......... .......... ....G..... .......... .......... .......... .......... ......AC..  [5680]
#L1MdGf_I    .......... .......... ....GC.... .......... .......... .......... .......... ......AC..  [5680]
#L1MdGf_II   .......... .......... ....G..... .......... .......... .......... .......... ......AC..  [5680]
#L1MdTf_I    .......... .......... ....G..... .......... .......... .......... .......... ......AC..  [5680]
#L1MdTf_II   .......... .......... ....G..... .......... .......... .......... .......... ......AC..  [5680]
#L1MdTf_III  .......... .......... ....G..... .......... .......... .......... .......... ......ACA.  [5680]
#L1MdA_V     .......... .......... ....G..... .......... .......... .......... .......... ....T.AG..  [5680]
#L1MdA_VI    .......... .......... ....G..... .......... .......... .......... .......... ......W...  [5680]
#L1MdF_V     ......G..T .......... ....G..... G......... .......... ...K...... .......... ....T.G...  [5680]
#L1MdF_IV    ......G..T .......... ....G..... G......... .......... ...K...... .......... ....T.....  [5680]
#L1MdF_I     .........T ..T....... ....G..... .......... .......... ......C... .......... ..........  [5680]
#L1MdF_II    .........T .......... ....G..... .......... .......... ......C... .......... ..........  [5680]
#L1MdF_III   .......... .......... ....G..... .......... .......... .......... .......... ..........  [5680]
#L1MdN_I     .......... .......... ....G..... .......A.. .......... .......... .......... ....T.AG..  [5680]
#L1MdA_VII   .......... .......... ....G..... .......... .......... .......... .......... ......AC..  [5680]
#L1MdMus_I   ..R...R..T .......... ....G..G.. G......... .......... ...T...G.. .........C ....T..A..  [5680]
#L1MdFanc_I  ......G..T .......... ....G..G.. G......... .......... ...Y...G.. .........C ....TH....  [5680]
#L1MdV_I     ......G..T .......... ....G..G.. G......... .......... ...T...G.. .........C ....T.....  [5680]
#L1MdFanc_II ......G..T ......T... ....G..G.. G.G....... .......... ...D...G.G .........- ....T.....  [5680]
#L1MdMus_II  .....-G..T ......T... ....G..G.. G.G....... .......... ...D...G.G .........- ....TH...Y  [5680]
#L1MdV_II    .DA..TD..G .........Y ....G..G.. G.G....... ....NV.T.. ...A...G.G .........- .D..T.C..T  [5680]
#L1Lx_I      .GA...V..K .......... ....G..G.. G.G....... .........Y.. ...A...G.G .........- ....T.B..T  [5680]
#L1Lx_III    .GA.C.A..G ...C...... ....G..G.. G.G....... .T..T..A.. ...A...G.G ........H- ....T.H..W  [5680]
#L1Lx_II     .GA.C.A..G ...C...C.. ....G..G.. G.G....... .T.....D.. ...A..DG.G .........- ....T.C.RT  [5680]
#L1Lx_IV     NGA.C.A..G .D..G..G.. ....G.GG.. G.G....... .T..T..A.. ...G...G.G ......N..- ....G.C..A  [5680]
#L1MdV_III   ---------- ...C...... ....G..G.. G.G....... .T...ATG.. ...A...G.R ......T..- ....T.C.AT  [5680]

#L1MdA_I     AAACTTTATA T----GCCCC AGTACAGGGG AATACCAGGG CCAAAAAGGG GGAGTGGGTG GGCAGGGG-- --AGTG----  [5760]
#L1MdA_II    .......... .----..... .......... ..CD...... .......... .......... ........-- --....----  [5760]
#L1MdA_III   .......... .----..... .......... ..CG...... .......... .......... ..T.....-- --....----  [5760]
#L1MdA_IV    .....G.... .----..... .......... ..CG...... .......AAA AA.AAAT.G. AATG..T.-- --G..A----  [5760]
#L1MdGf_I    .......... .----..... .......... ..CG...... ...T...A.. .......... ..T.....-- --..A.----  [5760]
#L1MdGf_II   .......... .----..... .......... ..CG...... .......... .......... .KT.....-- --.K..----  [5760]
#L1MdTf_I    .G.....G.G .----..... G......... ..CG...... .....GG... .......... ..T.....-- --....----  [5760]
#L1MdTf_II   .G.....G.G .----..... G......... ..CG...... .....GG... .......... ..T.....-- --....----  [5760]
#L1MdTf_III  .......... .----..... ..A....... ..CG...... .......... .........C. ..T.....-- --....----  [5760]
#L1MdA_V     .......... .----..... .......... ..CG...... .......AT. ...A...... ..T.....-- --....----  [5760]
#L1MdA_VI    .......... .----..... .......... ..CG...... ........T. .......... ..T.....-- --....----  [5760]
#L1MdF_V     .......... .----...T. ....Y..... ..CG...... ....G...T. .......... ..T.....-- --T.G.----  [5760]
#L1MdF_IV    .......... .----...T. .......... ..CG...... ....G...T. ........K. ..TV....-- --R.KK----  [5760]
#L1MdF_I     .......... .----..... .......... ..CG...... .......... .S........ ..T.....-- --.T..----  [5760]
#L1MdF_II    .......... .----..... .......... ..CG...... .......... .......... ..T.....-- --....----  [5760]
#L1MdF_III   .......... .----..... .......... ..HG...... ........T. .......... ..T.....-- --....----  [5760]
#L1MdN_I     ....G..... .----..... .......... ..CG...... .......AT. ...A...... ..T.....-- --....----  [5760]
#L1MdA_VII   .......... .----..... .......... ..CG...... ........T. ...A...... ..T.....-- --....----  [5760]
#L1MdMus_I   .......... .----..... .......... ..CG...... ....G...T. ......A... ..T.....-- --..CA----  [5760]
#L1MdFanc_I  .......... .----..... .......... ...G...... ....G...T. .......... ..T.....-- --..CA----  [5760]
#L1MdV_I     .......... .----..... .......... ..C....... ....G...T. .......... ..T.....-- --..C.----  [5760]
#L1MdFanc_II G..GA..... .----..... .......... ...G...... ...GG...CA .......... ..TT....-- --..CA----  [5760]
#L1MdMus_II  G..GA.Y... .----..... .......... ...G.....D ...GG...C. .........D ..TTD...-- --..CA----  [5760]
#L1MdV_II    G..GGN.C.. .----..... ....T..... ...G.....V ..GGG..-T. .......... ..TT....-- --..CA----  [5760]
#L1Lx_I      G..GG..C.. .----.H... ....T..... ...G...... ...GG...C. .......... ..TT..K.-- --..CA----  [5760]
#L1Lx_III    G..GGC.CD. .----.H... .D.GT..R.. ...G.....R ..GGG...C. .......... ..TT..T.-- --..CA----  [5760]
#L1Lx_II     G..GGC.CG. .----K.... ...GT..D.. ...G....NA ..GGD...C. .......... ..TTV.T.-- --..CA----  [5760]
#L1Lx_IV     GGDDG..TR. .----..... ...GT..... ...GBB.... .AGG..GAC. .......... D.TG....-- --..CACCCT  [5760]
#L1MdV_III   G..GGC.CG. .AGAT..... ...GT..... ...-TG.... .GGGG.G.T. .......... ..TG..T.GA GG.ACACCCT  [5760]
```

```
#L1MdA_I      ---------- GGG------G TGGGTGGATA TGGGGGACT- ---TTTGGTA TAGCATTGGA AATGTAAATG AGTTAAATAC  [5840]
#L1MdA_II     ---------- ...------. .......... .........- ---....... .......... .......... ..C.......  [5840]
#L1MdA_III    ---------- ...------. .......G.. .........- ---....... .......... .......... ..C.......  [5840]
#L1MdA_IV     ---------- ...AAGTGG. G...A..G.. .........- ---.....G. .......... .......T.. ..GA.....T  [5840]
#L1MdGf_I     ---------- ...------. .......C.. .........- ---....... ........C. .......... ..CG......  [5840]
#L1MdGf_II    ---------- ...------. .......G.. .........- ---....... .......... .......... ..C.......  [5840]
#L1MdTf_I     ---------- ...------. .......G.. A........- ---....... .......... .......... ..C.......  [5840]
#L1MdTf_II    ---------- ...------. .......G.. A........- ---....... .......... .......... ..C.......  [5840]
#L1MdTf_III   ---------- ...------. .......G.. .........- ---....... .......... .......... ..C.......  [5840]
#L1MdA_V      ---------- ...------- G...A..G.G .........- ---.....G. .......... .......T.. ..GA......  [5840]
#L1MdA_VI     ---------- ...----GK. G...A..G.. .........- ---.....G. .......... .......... ..GA......  [5840]
#L1MdF_V      ---------- ...------- --..G..G.. .........- ---.....G. .....Y.... .......... .AGA......  [5840]
#L1MdF_IV     ---------- K.KGG----- G...G..K.T .........- ---.....G. .......... .......... .AGA......  [5840]
#L1MdF_I      ---------- ...G------ -......G.. ........C- ---.....G. ........A. ........C. ..GA......  [5840]
#L1MdF_II     ---------- ...GT----- -......G.. .........- ---....... .......... .......... ..C......-  [5840]
#L1MdF_III    ---------- ...G------ -...A..G.. .........- ---.....G. .......... .......... ..GA.....A  [5840]
#L1MdN_I      ---------- ...------- G...------ --.......- ---.....G. .......... .......T.. .AGA......  [5840]
#L1MdA_VII    ---------- ...------. G...A..G.. .........- ---.....G. .......... .......T.. ..GA......  [5840]
#L1MdMus_I    ---------- ...CG----. G...A..G.. .A.......- ---.....G. .......T.. .......... .AGA.....T  [5840]
#L1MdFanc_I   ---------- ...CG----. G...A..G.. .A...A...- ---..C..G. .......T.. ......T..A .AGA.....T  [5840]
#L1MdV_I      ---------- ...DG----. G...A..G.. .A.......- ---..C..G. .......T.. .......... .AGA......  [5840]
#L1MdFanc_II  ---------- ...CG----. G...A..G.. .A.......- ---..C..G. .......T.. .......... .AGA.....T  [5840]
#L1MdMus_II   ---------- ...CG----. G...A.NG.. .A.D..N..- ---..G..G. .......T.. .......... .AGA.....T  [5840]
#L1MdV_II     ---------- ...GGAGGN. GDA.G..... G...ATTT.- ---CGGA.GG G.AAC.A... ..G.GG.NAA CA..TG.A.T  [5840]
#L1Lx_I       ---------- ...GGAGCG. G.A.GD.... G...ATTT.- ---CGGA.DG G.AAC.A... ..G.GG.TAA CA..TG.A.T  [5840]
#L1Lx_III     ---------- ..DGDAGGG. DKABGR.... G....TTT.- ---CGGA.GG G.AACCA... ..G.GG.TAA YA..TG.A.T  [5840]
#L1Lx_II      ---------- ...GGAGGNN RNAKG..... GD...NTT.- ---CGGADGG G.AABBA... ..G.GG.TAA CA..TG.A.T  [5840]
#L1Lx_IV      CATAGARGCA ...GGACGG. C.ATG..... G....TTTC- ---YAAA.GG G..ACC.... ..G.GG..AA CA..TG.A.T  [5840]
#L1MdV_III    CATAGAANCA ...GGAGGRA G.ATGD.... G..D.TTTCT DGGNGG..GR G.AACNG... ..G.GG.TAA CA..TG.A.T  [5840]

#L1MdA_I      CTAATAAAAA ATGGAAAAAA A [5861]
#L1MdA_II     .......... .......... . [5861]
#L1MdA_III    .......... .......... . [5861]
#L1MdA_IV     G......... .WWWTW.... . [5861]
#L1MdGf_I     .......... .......... . [5861]
#L1MdGf_II    .......... .D.R...... . [5861]
#L1MdTf_I     .......... .......... . [5861]
#L1MdTf_II    .......... ...D...... . [5861]
#L1MdTf_III   .......... .......G.. . [5861]
#L1MdA_V      G......... .AAAW..W.. . [5861]
#L1MdA_VI     .....W.... .AWWWW.... . [5861]
#L1MdF_V      .....T.... .AAA...... . [5861]
#L1MdF_IV     .....T.... .AAA...... . [5861]
#L1MdF_I      .....W.... .AAA...... . [5861]
#L1MdF_II     .CT.AT.... .AT.GG.... . [5861]
#L1MdF_III    .CT.AT.... .AAA...... . [5861]
#L1MdN_I      G......... .AAA...... . [5861]
#L1MdA_VII    G......... .AAW.T.TTT . [5861]
#L1MdMus_I    .......... .--------- - [5861]
#L1MdFanc_I   .......... ---------- - [5861]
#L1MdV_I      .......... .WWDD..... . [5861]
#L1MdFanc_II  .......... .AANH.NH.. . [5861]
#L1MdMus_II   .......... WANAD.DDD. . [5861]
#L1MdV_II     G...AT...G .AAAT.TCT. . [5861]
#L1Lx_I       G...AT...G .AAAT.TCT. . [5861]
#L1Lx_III     G...AT...G .AAAT.TCT. . [5861]
#L1Lx_II      G...AT...G .AAAT.TCT. . [5861]
#L1Lx_IV      G...AT...G .AAAH.TVC. . [5861]
#L1MdV_III    B-..AT...G .AAAT.BCC. . [5861]
```
