# Supplementary material for: Revisiting the evolution of mouse LINE-1 in the genomic era
Source: Mob DNA. 2013 Jan 3;4:3. doi: 10.1186/1759-8753-4-3 (PMC3600994; doi:10.1186/1759-8753-4-3)
Supplement: Additional file 2 — Matrix of pairwise divergence based on the longest non-recombining fragment of ORF2 (from position 2,085 to 4,489 in Additional file 1). [file 1759-8753-4-3-S2.pdf]

Supplementary material 2. Matrix of pairwise divergence based on the longest non-recombining fragment of ORFII (from position 2085 to 4489 in supplementary material 1)

| | | [1] | [2] | [3] | [4] | [5] | [6] | [7] | [8] | [9] | [10] | [11] | [12] | [13] | [14] | [15] | [16] | [17] | [18] | [19] | [20] | [21] | [22] | [23] | [24] | [25] | [26] | [27] | [28] |
|---|---|---|---|---|---|---|---|---|---|---|---|---|---|---|---|---|---|---|---|---|---|---|---|---|---|---|---|---|---|
| [1] | L1MdA_I | | | | | | | | | | | | | | | | | | | | | | | | | | | | |
| [2] | L1MdA_II | 0.25 | | | | | | | | | | | | | | | | | | | | | | | | | | | |
| [3] | L1MdA_III | 0.71 | 0.50 | | | | | | | | | | | | | | | | | | | | | | | | | | |
| [4] | L1MdA_IV | 1.52 | 1.31 | 0.97 | | | | | | | | | | | | | | | | | | | | | | | | | |
| [5] | L1MdGf_I | 1.77 | 1.56 | 1.26 | 1.52 | | | | | | | | | | | | | | | | | | | | | | | | |
| [6] | L1MdGf_II | 0.88 | 0.59 | 0.25 | 0.97 | 1.43 | | | | | | | | | | | | | | | | | | | | | | | |
| [7] | L1MdTf_I | 2.12 | 1.90 | 1.48 | 1.78 | 1.77 | 1.65 | | | | | | | | | | | | | | | | | | | | | | |
| [8] | L1MdTf_II | 2.16 | 1.95 | 1.52 | 1.78 | 1.82 | 1.69 | 0.04 | | | | | | | | | | | | | | | | | | | | | |
| [9] | L1MdTf_III | 1.74 | 1.52 | 1.10 | 1.27 | 1.39 | 1.18 | 1.48 | 1.52 | | | | | | | | | | | | | | | | | | | | |
| [10] | L1MdA_V | 2.94 | 2.73 | 2.47 | 1.44 | 2.95 | 2.47 | 3.12 | 3.16 | 2.73 | | | | | | | | | | | | | | | | | | | |
| [11] | L1MdA_VI | 3.39 | 3.17 | 3.00 | 1.78 | 3.39 | 2.99 | 3.83 | 3.83 | 3.26 | 1.99 | | | | | | | | | | | | | | | | | | |
| [12] | L1MdF_V | 4.23 | 4.01 | 3.83 | 2.73 | 4.32 | 3.83 | 4.76 | 4.76 | 4.01 | 2.90 | 1.05 | | | | | | | | | | | | | | | | | |
| [13] | L1MdF_IV | 4.23 | 4.01 | 3.83 | 2.69 | 4.32 | 3.83 | 4.76 | 4.76 | 4.06 | 2.90 | 0.97 | 0.00 | | | | | | | | | | | | | | | | |
| [14] | L1MdF_I | 4.98 | 4.75 | 4.49 | 3.59 | 5.07 | 4.49 | 5.38 | 5.43 | 4.76 | 4.08 | 2.80 | 2.23 | 2.23 | | | | | | | | | | | | | | | |
| [15] | L1MdF_II | 6.51 | 6.23 | 6.10 | 5.05 | 6.46 | 6.10 | 6.92 | 6.97 | 6.24 | 5.09 | 3.62 | 2.70 | 2.66 | 2.50 | | | | | | | | | | | | | | |
| [16] | L1MdF_III | 5.31 | 5.09 | 4.91 | 3.79 | 5.31 | 4.91 | 5.85 | 5.90 | 5.14 | 3.92 | 2.34 | 1.31 | 1.31 | 1.41 | 1.57 | | | | | | | | | | | | | |
| [17] | L1MdN_I | 3.12 | 2.90 | 2.73 | 1.69 | 3.12 | 2.73 | 3.38 | 3.42 | 2.99 | 0.38 | 2.04 | 2.86 | 2.86 | 4.08 | 5.05 | 3.88 | | | | | | | | | | | | |
| [18] | L1MdA_VII | 2.77 | 2.56 | 2.30 | 1.10 | 2.77 | 2.30 | 3.12 | 3.12 | 2.56 | 1.27 | 0.67 | 1.61 | 1.56 | 2.93 | 4.19 | 2.82 | 1.48 | | | | | | | | | | | |
| [19] | L1MdMus_I | 7.60 | 7.60 | 7.47 | 6.38 | 7.79 | 7.46 | 8.21 | 8.21 | 7.38 | 6.79 | 4.93 | 3.98 | 4.12 | 5.70 | 6.36 | 5.20 | 6.80 | 5.42 | | | | | | | | | | |
| [20] | L1MdFanc_I | 7.70 | 7.61 | 7.43 | 6.34 | 7.75 | 7.33 | 8.22 | 8.22 | 7.34 | 6.76 | 4.98 | 4.08 | 4.21 | 5.66 | 6.46 | 5.26 | 6.76 | 5.38 | 1.27 | | | | | | | | | |
| [21] | L1MdV_I | 5.24 | 4.93 | 4.75 | 3.76 | 5.15 | 4.75 | 5.61 | 5.61 | 4.89 | 4.12 | 2.22 | 1.10 | 1.23 | 3.21 | 3.69 | 2.40 | 4.07 | 2.74 | 2.84 | 2.80 | | | | | | | | |
| [22] | L1MdFanc_II | 6.77 | 6.64 | 6.46 | 5.48 | 6.87 | 6.46 | 7.33 | 7.33 | 6.60 | 5.84 | 4.17 | 3.06 | 3.19 | 4.66 | 5.27 | 4.18 | 5.89 | 4.61 | 1.96 | 1.91 | 1.75 | | | | | | | |
| [23] | L1MdMus_II | 5.72 | 5.63 | 5.45 | 4.42 | 5.81 | 5.40 | 6.28 | 6.28 | 5.55 | 4.93 | 3.11 | 2.09 | 2.22 | 3.87 | 4.53 | 3.30 | 4.93 | 3.56 | 2.04 | 2.04 | 0.95 | 0.69 | | | | | | |
| [24] | L1MdV_II | 7.21 | 7.07 | 6.89 | 5.83 | 7.21 | 6.88 | 7.79 | 7.79 | 6.99 | 6.21 | 4.63 | 3.62 | 3.76 | 4.95 | 5.81 | 4.54 | 6.21 | 4.90 | 2.26 | 2.26 | 2.36 | 0.91 | 1.24 | | | | | |
| [25] | L1Lx_I | 7.49 | 7.35 | 7.17 | 6.31 | 7.54 | 7.17 | 8.07 | 8.07 | 7.32 | 6.45 | 4.97 | 3.92 | 4.05 | 5.29 | 5.91 | 4.79 | 6.49 | 5.42 | 2.60 | 2.69 | 2.66 | 1.07 | 1.36 | 0.26 | | | | |
| [26] | L1Lx_III | 10.12 | 9.87 | 9.77 | 8.64 | 9.96 | 9.72 | 10.62 | 10.62 | 9.83 | 8.83 | 7.41 | 6.57 | 6.72 | 7.82 | 8.43 | 7.37 | 8.78 | 7.84 | 5.05 | 4.91 | 5.09 | 3.65 | 3.88 | 2.41 | 2.53 | | | |
| [27] | L1Lx_II | 9.24 | 8.99 | 8.98 | 8.00 | 9.07 | 8.98 | 9.91 | 9.91 | 9.10 | 8.20 | 6.76 | 6.01 | 6.11 | 7.27 | 7.84 | 6.86 | 8.15 | 7.19 | 4.73 | 4.48 | 4.56 | 3.11 | 3.37 | 2.12 | 2.06 | 0.69 | | |
| [28] | L1Lx_IV | 12.04 | 11.79 | 11.70 | 10.72 | 11.98 | 11.70 | 12.60 | 12.60 | 11.75 | 10.96 | 9.72 | 8.84 | 9.04 | 10.01 | 10.57 | 9.73 | 10.81 | 10.01 | 7.39 | 7.06 | 7.44 | 5.84 | 6.26 | 4.63 | 4.69 | 0.98 | 1.87 | |
| [29] | L1MdV_III | 12.60 | 12.45 | 12.31 | 11.47 | 12.54 | 12.35 | 13.16 | 13.16 | 12.47 | 11.67 | 10.04 | 9.16 | 9.30 | 10.53 | 10.83 | 10.10 | 11.63 | 10.57 | 7.61 | 7.66 | 7.71 | 6.20 | 6.47 | 4.86 | 4.87 | 2.47 | 2.76 | 3.22 |
