# Supplementary material for: Revisiting the evolution of mouse LINE-1 in the genomic era
Source: Mob DNA. 2013 Jan 3;4:3. doi: 10.1186/1759-8753-4-3 (PMC3600994; doi:10.1186/1759-8753-4-3)
Supplement: Additional file 3 — Alignments showing recombination break-points among L1 families. Only the parsimony-informative sites are shown. [file 1759-8753-4-3-S3.pdf]

Alignment of parsimony informative sites showing the recombinant origin of the Tf families. The arrow indicates the position of the recombination breakpoint.

```
             1111111111 1111111111 1222222222 2222222222 2222
             7777778888 9999999999 9000000111 1111222222 2223
             3345791377 0011235677 8011249034 4789000033 4551
             5600850769 0669927978 4212915411 3366038913 2187
L1MdTf_I     ACTACGTCTT CATGGTTTCA GACAACAAGC TGATGAGGTC TTAT
L1MdTf_II    .......T.. .......... .......... .......... ....
L1MdTf_III   .......T.. ..A....... ....G..... ..CC.G.... ....
L1MdF_II     ....GK.T.. ..A..WC... ..T.G.G.AT AACCAGTACT C.MC
L1MdF_I      ....G.CT.. .TAATACYG. ..T.G.GGAT AACCAGTACT C.G.
L1MdF_III    ....GT.T.. .TAATAC.G. ..T.G.GGAT AACCAGTACT C.CY
L1MdA_I      TTAGGTCTCG TTA..A.CGC ACTGGT.... A.CC.G.... .C..
L1MdA_II     TTDGGTCTCG TTA..A.CGC ACTGGT.... A.CC.G.... .C..
L1MdA_III    .T.GGTCTCG TTA..A.CGC ACTGG..... ..CC.G.... ....
```

Alignment of parsimony informative sites showing the recombinant origin of the Gf_I family. The arrow indicates the position of the recombination breakpoint.

```
                      ORFI                           ORFII
                    111 1111111111 1111111111 1111111111 1111111111 11111
             7999999001 1111111222 2222222333 3344444455 5555555666 66667
             9014469351 2237789001 1223378336 8912348811 1222469023 35683
             7182468793 2510381474 5454617397 4643283401 9058675970 79355
L1MdA_I      TTGAGCGGGC AATGGATCCA GATCACAATG GCCGCGACTT GGACTACCCA GGCTT
L1MdA_II     .......... .......... .......... .......... .......... .....
L1MdA_III    .......... ........G. .CC....... ......T..C .........T .A.CA
L1MdA_IV     ...C...... .T.AA..AG. .CC....... ......T..C .A......TC .A.CA
L1MdGf_I     ACACAT..AT CTCAMGCAAG ACM.TTC... ......T..C .........T .A.CA
L1MdGf_II    ACACAT.TAT CTCACGCAAG ACCATTCTC. ......T... .........T .A.CA
L1MdF_I      ACACAT.TAT CTCACGCAAG ACCATTCTCA ATTATATAYC T.GTHG..TT AAACA
L1MdF_II     ACACAT.TA. .TCACGCAAG .CC.TTCTCA ATTATATACC T.GTCG.MTT AAACA
L1MdF_III    ACACAT.TA. .T.AC.CAGG .CC.TTY.C. .TTATATA.C TA..CG.ATT AAACA
```

Alignment of parsimony informative sites showing the recombinant origin of the Gf_II family. The arrow indicates the position of the recombination breakpoint.

```
                      ORFI                           ORFII
                    111 1111111111 1111111111 1111111111 1111111111 11111
             7999999001 1111111222 2222222333 3344444455 5555555666 66667
             9014469351 2237789001 1223378336 8912348811 1222469023 35683
             7182468793 2510381474 5454617397 4643283401 9058675970 79355
L1MdA_I      TTGAGCGGGC AATGGATCCA GATCACAATG GCCGCGACTT GGACTACCCA GGCTT
L1MdA_II     .......... .......... .......... .......... .......... .....
L1MdA_III    .......... ........G. .CC....... ......T..C .........T .A.CA
L1MdA_IV     ...C...... .T.AA..AG. .CC....... ......T..C .A......TC .A.CA
L1MdGf_I     ACACAT..AT CTCAMGCAAG ACM.TTC... ......T..C .........T .A.CA
L1MdGf_II    ACACAT.TAT CTCACGCAAG ACCATTCTC. ......T... .........T .A.CA
L1MdF_I      ACACAT.TAT CTCACGCAAG ACCATTCTCA ATTATATAYC T.GTHG..TT AAACA
L1MdF_II     ACACAT.TA. .TCACGCAAG .CC.TTCTCA ATTATATACC T.GTCG.MTT AAACA
L1MdF_III    ACACAT.TA. .T.AC.CAGG .CC.TTY.C. .TTATATA.C TA..CG.ATT AAACA
```

Alignment of parsimony informative sites showing recombination between recent L1 families at the 3'end of ORF2 and in the 3'UTR. The arrows indicate the position of the recombination breakpoints.

```
                              ORFII                             3'UTR
            4444444444 4444444444 4444444444 5555555555 5555555555 5555555555 5555555
            3333444444 4455555556 6778888999 0000000000 0111111111 1111111222 2222222
            3347111356 8901244582 7293345145 0123556777 8122234444 5678899001 1256668
            0625017887 7470826281 8583400827 1315697578 0715781459 4430159130 5112561
L1MdA_I     CTCACTACAT AAAGTTAGGC AGACCAGCGC TCACATGCCA TCGCCATTTT ACTTGACCCT GAGATGG
L1MdA_II    .......... .......... T....T.... .......... .......... .......... .......
L1MdA_III   .......... .......... T....T.... .......... ......C... .........A .......
L1MdA_IV    .C........ .......... ...T.C...T .......... ......C... .........A ....C.S
L1MdA_V     GC..T..TGC CGG.CATAAT .AHT.C..AT CGT.CAAATG ATTGGGCKAA .GCCATY.TA AT..CAC
L1MdN_I     GC..T..TGC CGG.GATAAT .ACT.C.TAT CGT.CAAATG ATTGGGC.AA .GCCATTATA AT..CAC
L1MdA_VI    GCTG...T.C ..GA..TA.T .ACTTT..AT .GT.CAAATG .TA..WC..A ..CCAT..TA ....CAC
L1MdF_I     GC.G.CGT.C ..G....... .ACT.CA..T .G.TCA.... .T....CC.C G.CCAT.TTA ..ATC.C
L1MdF_II    GC...CGT.C ....C..... .A...C...T ....CA.... .T....Y... ..CCAT..TA A.ATC.C
L1MdF_III   GC.....T.C ..G....... .ACTYC...T .G..CA.... .T....C... G.YCAK..TA ...TC.C
L1MdF_IV    GCTG...T.C ..GA..T... .ACTTC...T .G..CAA.TG .TA...C..G ..CCAT..TA ....C.C
L1MdV_I     GCTG...T.C ..GA..T... .A.TTTVT.T .G.TCAA.TG .TA...CB.A ..CCAT..TA ....C.C
                            ↑                                      ↑
```

Alignment of parsimony informative sites showing the transfer of an ancestral coiled-coil domain (framed in blue) to the L1MdA_VI, L1MdA_VII, L1MdGf_II and L1MdF_III families. The arrows indicate the position of the recombination breakpoints.

```
                          ORFI
                                                      11
                     1 2222222444 4446666777 7777789999 00
            1236778890 1145667112 2781129223 3578890146 35
            2695891406 4769013276 8106729392 8643641846 79
L1MdA_IV    CCTGCCATTA GTGAAAAACA TCGACTTTGA TACAGTTGGC GG
L1MdA_V     ....G..... .......... .....C...G ...TCG.... ..
L1MdF_II    TTCCTTGCAC .......... .TAGG.GCA. CTT...CAAT TA
L1MdF_III   TTCCTTGCAC CGATTG.TTC AT.GV.ACAG ......CAAT TA
L1MdGf_II   TTCCTTGCAC CGATT..TTC ATAGG.GCA. CTT...CAAT TA
L1MdA_VII   .........C CGATTGGTTC AT...C.... ...TCG.... ..
L1MdA_VI    .TC....C.C CGATTGGTTC AT....A... .......A.. .A
                      ↑           ↑
```
